# Simultaneous Analysis of 504 Pesticide Multiresidues in Crops Using UHPLC-QTOF at MS^1^ and MS^2^ Levels

**DOI:** 10.3390/foods13213503

**Published:** 2024-10-31

**Authors:** Mun-Ju Jeong, Su-Min Kim, Ye-Jin Lee, Yoon-Hee Lee, Hye-Ran Eun, Miok Eom, Gui-Hyun Jang, JuHee Lee, Hyeong-Wook Jo, Joon-Kwan Moon, Yongho Shin

**Affiliations:** 1 Pesticide and Veterinary Drug Residues Division, Food Safety Evaluation Department, National Institute of Food and Drug Safety Evaluation, Ministry of Food and Drug Safety, Cheongju 28159, Republic of Korea; mjjeong98@korea.kr (M.-J.J.); miokeom@korea.kr (M.E.); arion@korea.kr (G.-H.J.); jhfuture@korea.kr (J.L.); 2 Department of Applied Bioscience, Dong-A University, Busan 49315, Republic of Korea; 3 Hansalim Agro-Food Analysis Center, Hankyong National University Academic Cooperation Foundation, Suwon 16500, Republic of Korea; hyeongwook.jo@hknu.ac.kr; 4 Department of Plant Resources and Landscape Architecture, Hankyong National University, Anseong 17579, Republic of Korea

**Keywords:** crop, HRMS, library, pesticide multiresidues, UHPLC-QTOF, food safety

## Abstract

A robust analytical method was developed for the simultaneous detection of 504 pesticide multiresidues in various crops using ultra-high-performance liquid chromatography coupled with quadrupole time-of-flight mass spectrometry (UHPLC-QTOF). The method integrates both MS^1^ and MS^2^ levels through sequential window acquisition of all theoretical mass spectra (SWATH) analysis, allowing for accurate mass measurements and the construction of a spectral library to enhance pesticide residue identification. An evaluation of the method was carried out according to international standards, including the FAO guidelines and SANTE/11312/2021. Validation across five representative crops—potato, cabbage, mandarin, brown rice, and soybean—demonstrated exceptional sensitivity, with over 80% of the analytes detected at trace levels (≤2.5 μg/kg). Moreover, an impressive 96.8% to 98.8% of the compounds demonstrated LOQs of ≤10 μg/kg. Most compounds exhibited excellent linearity (*r*^2^ ≥ 0.980) and satisfactory recovery rates at spiking levels of 0.01 and 0.1 mg/kg. Among 42 crop samples analyzed, pesticides were detected in 1 cabbage, 3 mandarin, and 6 rice samples, with a mass accuracy within ±5 ppm and a Fit score ≥ 70.8, confirming the method’s practical applicability and reliability. The detected residues ranged from 12.3 to 339.3 μg/kg, all below the established maximum residue limits (MRLs). This comprehensive approach offers an efficient, reliable, and scalable solution for pesticide multiresidue monitoring, supporting food safety programs and regulatory compliance.

## 1. Introduction

The widespread use of pesticides in agriculture has undeniably increased crop yields and protected plants from pests and diseases [1,2]. However, the residual presence of these chemicals in agricultural products poses significant concerns for human health and environmental safety, particularly regarding chronic health risks to consumers [3]. Modern agricultural practices often involve the simultaneous or sequential application of multiple pesticides to the same crop to control a variety of pests, weeds, and diseases. This leads to the presence of multiple pesticide residues in a single crop, underscoring the necessity of multiresidue analysis rather than testing individual compounds separately [4]. The globalization of the food supply chain further intensifies the need for the development of robust multiresidue pesticide analysis, as internationally traded crops must meet diverse regulatory requirements across different countries and regions [5]. Simultaneous multiresidue analysis is more efficient and cost-effective, allowing for the high-speed scanning, detection, and quantification of a wide spectrum of pesticides within a single analytical run [6]. This comprehensive approach is essential for rapidly and accurately assessing the overall exposure risk to consumers and for ensuring compliance with regulatory standards that establish maximum residue limits (MRLs) for a wide range of pesticides.

Ultra-high-performance liquid chromatography (UHPLC) is adept at analyzing a broad spectrum of substances, encompassing both polar and non-polar analytes. It is particularly effective for the separation and detection of organic compounds, including pesticides and their metabolites [7]. For highly selective trace analysis, UHPLC can be coupled with mass spectrometry (MS). Among the various MS techniques, high-resolution mass spectrometry (HRMS) is the preferred method for both targeted and non-targeted screening of pesticide residues and their metabolites in food samples [8]. It offers several significant advantages that enhance the analysis of target compounds in food samples. HRMS provides exceptional mass accuracy and resolving power, enabling precise identification and differentiation of compounds with very similar mass-to-charge (*m*/*z*) ratios [9,10]. This high level of specificity minimizes the likelihood of false positives and ensures reliable detection of target analytes amidst complex sample matrices [11]. Additionally, recent advancements in HRMS have resulted in improved detecting sensitivity, allowing for the detection of trace levels of pesticide residues [12].

A commonly utilized MS technique for the simultaneous determination of hundreds of pesticides is the triple quadrupole mass spectrometer (TQ MS) [13]. When operated in the multiple reaction monitoring (MRM) mode, TQ MS selectively detects specific precursor and product ions, thereby enhancing both the selectivity and sensitivity for targeted compounds [14]. This makes TQ MS highly effective for quantitative analysis of known pesticides within complex sample matrices [15]. However, the MRM approach requires time-consuming establishment of individual MRM conditions for each analyte, and is inherently limited to predefined target analytes, which restricts its ability to identify unexpected or unknown compounds present in the samples [16]. In contrast, HRMS offers full-spectrum data acquisition, enabling target, suspect, and non-target screening of pesticide residues [8]. This comprehensive data collection allows for retrospective data analysis, meaning that analysts can revisit the acquired data to search for additional or emerging contaminants without the need to re-analyze the samples [17]. Such capability is particularly valuable for identifying previously unmonitored pesticide residues or novel contaminants that may arise over time. Overall, HRMS enhances the versatility and efficiency of pesticide residue analysis, making it a powerful tool for comprehensive food safety monitoring.

Quadrupole time-of-flight mass spectrometry (QTOF MS) delivers high resolution (>30,000 full width at half maximum, FWHM) and exceptional mass accuracy (within ±5 ppm), both of which are essential for isotope analysis in complex organic mixtures [18]. For the rapid and precise identification of pesticide residues, spectral library searching serves as a key tool. The characteristics of compounds in monitoring samples were meticulously verified against those in an in-house TOF library by evaluating parameters such as mass accuracy, isotope patterns, MS/MS fragmentation patterns, and library purity scores, thereby validating the reliability of library matching rates and ensuring the dependable identification of pesticide residues [19]. QTOF MS offers a cost-effective solution without compromising analytical performance, enhancing its practicality for both targeted and non-targeted pesticide residue analysis and supporting robust and scalable food safety programs.

The primary objective of this study was to develop a comprehensive method for the multiresidue analysis of pesticides in various agricultural products using UHPLC-QTOF. The method was evaluated by assessing the mass accuracy of isotopic species, distinguishing isomeric pesticides, and constructing a spectral library to enhance the reliability of pesticide identification. The validated method was applied to five representative crops, demonstrating its suitability for routine pesticide monitoring at trace levels. By expanding the number of target analytes to over 500, this study aims to broaden the scope of pesticide residue analysis, providing valuable insights for regulatory adherence and enhancing food safety.

## 2. Materials and Methods

### 2.1. Chemicals and Reagents

Individual pesticide standards (analytical grade) and stock solutions (100–1000 µg/mL) were obtained from Accustandard (New Haven, CT, USA), Sigma-Aldrich (St. Louis, MO, USA), FUJIFILM Wako Pure Chemical Corporation (Osaka, Japan), LGC Standards (Wesel, Germany), HPC Standards (Cunnersdorf, Germany), and Toronto Research Chemicals (Toronto, ON, Canada). Ammonium formate (10 M, BioUltra grade) was purchased from Sigma-Aldrich (St. Louis, MO, USA), while formic acid (≥99.0%) and methanol (LC-MS grade) were sourced from Thermo Fisher Scientific (Waltham, MA, USA). Acetonitrile (HPLC grade) was acquired from Duksan Pure Chemical (Seoul, Republic of Korea), and LC-MS-grade water was purchased from Merck (Darmstadt, Germany). The QuEChERS extraction pouch (EN 15662), containing 4 g MgSO_4_, 1 g NaCl, 1 g sodium citrate dihydrate (Na_3_Citrate·2H_2_O), and 0.5 g sodium hydrogen citrate sesquihydrate (Na_2_HCitrate·1.5H_2_O), as well as the Fruits and Veg EN d-SPE tube (150 mg MgSO_4_ and 25 mg primary secondary amine; PSA), were obtained from Agilent Technologies (Santa Clara, CA, USA).

### 2.2. Preparation of Multiresidue Working Solution and Matrix-Matched Standard Solution

Individual pesticide standards were dissolved in acetone, acetonitrile, or methanol using a volumetric flask to prepare stock solutions at a concentration of 1000 μg/mL, which were then transferred to 20 mL amber glass vials. Indaziflam (purity: 99.6%), lepimectin (98.0%), pyribencarb metabolite (pyribencarb Z; 97.7%), and tefuryltrione (99.7%) were each prepared at a concentration of 100 µg/mL. These solutions and commercial stock solutions were combined to create an intermediate mixed solution at a concentration of 10 µg/mL, containing approximately 100 analytes per group. By diluting these mixed solutions, a mixed working solution of 2 µg/mL was prepared. Subsequent dilutions were made using acetonitrile to achieve final concentrations of 200, 125, 100, 50, 25, 20, 12.5, 10, 5, 2.5, and 1.25 ng/mL. Matrix-matched standard solutions were prepared by mixing the extract from pesticide-free samples with the standard solution. All solutions prepared were stored at −20 °C until use.

### 2.3. Representative Crop Samples and Sample Preparation

For method validation, five representative crops—potato, Korean cabbage, mandarin, brown rice, and soybean—were purchased from local markets and online marketplaces in the Republic of Korea. To obtain control samples without pesticide residues, eco-friendly or pesticide-free labeled crops were prioritized. For the application of the multiresidue method, a total of 42 samples from various crops were collected from distribution channels, comprising 7 potato samples, 4 cabbage samples, 4 mandarin samples, 26 rice samples, and 1 bean sample.

All samples were finely chopped, thoroughly homogenized using dry ice in a blender, and stored at −20 °C until further use. The homogenized samples (10 g) were weighed and placed into 50 mL centrifuge tubes. For the brown rice, an additional 10 mL of distilled water was added, and the sample was moistened for 1 h (for beans, 5 g of sample was used and moistened with 10 mL of water). Afterward, 10 mL of acetonitrile was added to the tube, and the mixture was vortexed and extracted for 2 min at 1300 rpm using a high-speed shaker (Mini-G 1600, SPEX SamplePrep, Metuchen, NJ, USA). A QuEChERS EN 15662 pouch containing MgSO_4_, Na_3_Citrate·2H_2_O, and Na_2_HCitrate·1.5H_2_O was then added, followed by vortex extraction for another 2 min at 1300 rpm. The sample was centrifuged at 3500 rpm for 5 min using a LABOGENE 1248 centrifuge (LABOGENE, Lillerød, Denmark). Subsequently, 1 mL of the acetonitrile layer was transferred into a d-SPE tube containing 150 mg of MgSO_4_ and 25 mg of PSA, mixed for 1 min and centrifuged at 13,000 rpm for 5 min using a microcentrifuge (X15R, Hanil Science, Incheon, Republic of Korea). After the cleanup, matrix-matching was performed by mixing 300 µL of the sample extract with 300 µL of acetonitrile in an autosampler vial (for beans, matrix-matching was adjusted by mixing 500 µL of sample extract with 125 µL of acetonitrile).

### 2.4. UHPLC-QTOF Instrumental Conditions

Qualitative and quantitative analyses of multiresidues were performed using a Nexera X3 UHPLC system (Shimadzu, Kyoto, Japan) coupled with an X500R QTOF mass spectrometer (AB SCIEX Co., Framingham, MA, USA). The UHPLC device included a solvent delivery pump (LC-40B), column oven (CTO-40C), system controller (SCL-40), and autosampler (SIL-40C). A Halo C18 column (2.1 × 150 mm, 2.7 μm particle size; Advanced Materials Technology, Wilmington, DE, USA) was used for compound separation, with the column oven set to 40 °C. The mobile phase consisted of the following two solutions: (A) 0.1% formic acid and 5 mM ammonium formate in water, and (B) 0.1% formic acid and 5 mM ammonium formate in methanol. Multiresidue separation was further facilitated by gradient elution, which dynamically adjusted the mobile phase composition. The gradient started at the 5% mobile phase B for 0.2 min, ramped to 50% B over 0.3 min, increased to 98% B over 13.5 min, and was maintained at 98% B for 3 min. The mobile phase rapidly decreased to 5% B within 0.1 min and held at 5% for 3.4 min to equilibrate the column. Each sample had a total run time of 20.5 min. The mobile phase flow rate was 0.2 mL/min, and the injection volume was 5 μL.

Ionization of analytes was performed using electrospray ionization (ESI) in both the positive and negative modes, depending on the molecular weight of the pesticide under analysis. The ion spray voltage (IS) was set to +5500 V in the positive mode and –4500 V in the negative mode. The ion source temperature was maintained at 550 °C, and gas pressures for curtain gas (CUR), collision-activated dissociation (CAD), and ion source gases 1 and 2 (GS1 and GS2) were set to 25, 10, 50, and 50 psi, respectively. The sequential window acquisition of all theoretical mass spectra (SWATH) mode was selected using the following parameters. For TOF MS (MS^1^), the mass-to-charge ratio (*m*/*z*) range was set from 100 to 1000, with a declustering potential (DP) of 80 V and a collision energy (CE) of 5 V. The accumulation time for each scan was 0.25 s. Time bins were summed across four channels (Channel 1 to 4). For the TOF MS/MS (MS^2^), the *m*/*z* range was from 50 to 1000, with an accumulation time of 0.25 s and a charge state of 1. A detailed breakdown of the mass table is provided in Table S1.

### 2.5. Verification of MS^1^ and MS^2^ Spectra and Construction of Pesticide Library Database

The MS^1^ spectra of target pesticides were acquired and compared using the X500R and LCMS-9030 (Shimadzu, Kyoto, Japan) QTOF mass spectrometer. The X500R achieved a resolution of 42,000 FWHM at *m*/*z* 956, while the LCMS-9030 provided a resolution of 30,000 FWHM at *m*/*z* 1972 and 1626. To acquire MS^2^ spectra, the precursor ion at the monoisotopic mass was subjected to a product ion scan using the X500R, with a CE spread ranging from 5 to 55 V. The resulting library included detailed information on the compounds, such as their common name, CAS number, molecular formula, molecular weight, and monoisotopic mass.

### 2.6. Analytical Method Validation

The established method was validated by assessing parameters such as the limit of quantification (LOQ), linearity of calibration curve, accuracy and precision, and matrix effect. The LOQ was determined by confirming the minimum concentration at which the signal-to-noise ratio (S/N) exceeded 10. The calibration curve was constructed using matrix-matched standard solutions and fitted using a linear regression with a weighting factor of 1/*x*. Linearity was assessed by calculating the correlation coefficient (*r*^2^). Accuracy and precision were evaluated by spiking samples with each analyte at concentrations of 10 and 100 μg/kg. The samples were then subjected to preparation using the established method, and the target analytes were analyzed to determine the recovery, which was calculated as the ratio of the measured value to the treated concentration (*n* = 3).

## 3. Results and Discussion

### 3.1. Accurate Mass Measurement with the Major Isotopic Species in the MS^1^ Analysis

Mass spectra at the MS^1^ stage were obtained for the target set of 504 pesticides. All monoisotopic species and singly substituted carbon-13 (^13^C) isotopic species exhibited a mass accuracy within ±5 ppm, which is well within the acceptable range specified by the SANTE guidelines (SANTE/11312/2021) [20]. These results confirm that the HRMS performance meets the required precision and accuracy standards, ensuring reliable analysis of pesticide residues.

**Figure 1:**
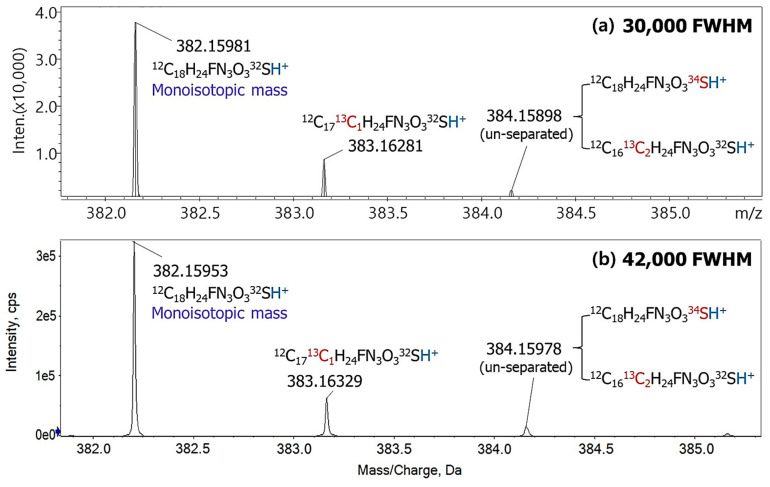
Spectra of benthiavalicarb-isopropyl in two resolutions at (**a**) 30,000 and (**b**) 42,000 FWHM. Red-highlighted atoms represent substituted atoms from monoisotopic species, and the blue-labeled proton (H^+^) represents an ionization adduct.

Some analytes containing heteroatoms, such as sulfur, showed mass accuracies exceeding ±5 ppm in heavier isotope species. For instance, benthiavalicarb-isopropyl (C_18_H_24_FN_3_O_3_S) has four major isotopic species (Table 1). Among these, the mass accuracies for the monoisotopic species and the singly substituted ^13^C species were between –0.16 ppm and +1.10 ppm, respectively, under 30,000 and 42,000 FWHM conditions, which are within acceptable limits. However, the mass accuracies for the singly substituted sulfur-34 (^34^S) species and double-substituted ^13^C species exceeded ±5 ppm, with values between –18.87 ppm and +11.61 ppm, respectively. This discrepancy arose because the theoretical *m*/*z* values of these two isotopes, 384.15532 and 384.16623, differed by only 0.01091, making them indistinguishable and resulting in their recognition as a single peak (Figure 1). Factors such as peak broadening, minor fluctuations in instrument calibration and stability further limit the effective resolution. Therefore, in the MS^1^ analysis using TOF instruments, special attention must be given to distinguishing isotopes of organic compounds containing heteroatoms, such as sulfur, chlorine, and bromine [21]. Higher-resolution instruments, such as an Orbitrap MS with a resolution of 240,000 or greater, can resolve these peaks, enabling a more precise molecular weight analysis [22].

**Figure 2:**
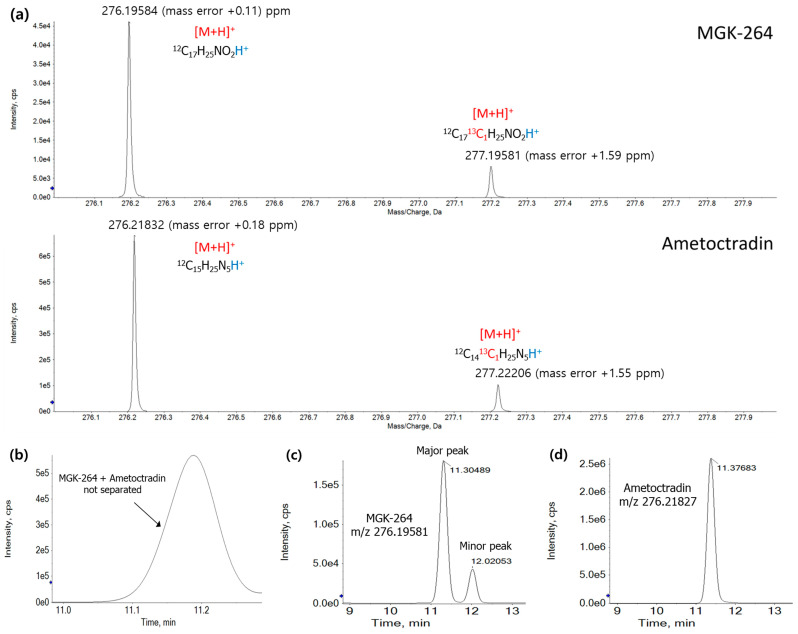
Differentiation of MGK-264 and ametoctradin using extracted ion chromatography (EIC) at high-mass resolution (42,000 FWHM): (**a**) spectra of MGK-264 and ametoctradin, showing ionization of the precursor ions ([M+H]^+^); (**b**) EIC showing the co-elution of these pesticides with low-mass accuracy (±500 ppm), resulting in no separation. The EICs of (**c**) MGK-264 and (**d**) ametoctradin at higher mass accuracies (±5 ppm), demonstrating clear separation between these analytes.

Since the monoisotopic mass is defined as the sum of the atomic weights of the lightest isotopic atoms found in nature, it is unaffected by the overlap of other isotopic mass peaks, thereby avoiding *m*/*z* distortion. Therefore, the mass error for the monoisotopic species for the target analytes was within ±5 ppm. Consequently, precise quantitative analysis could be performed at the MS^1^ level using extracted ion chromatography (EIC) of the monoisotopic masses of target pesticides. At a resolution of 42,000 using X500R QTOF, it was sufficient to differentiate between the precursor ions ([M+H]^+^) of MGK-264 (theoretical *m*/*z* 276.19581) and ametoctradin (*m*/*z* 276.21827), which had a mass difference of only 0.02246 and a retention time (t_R_) difference of 0.07 min (Figure 2). When the low-mass accuracy was set to ±500 ppm, the two mixtures could not be distinguished on the chromatogram (Figure 2b). However, improving the mass accuracy to ±5 ppm enabled individual detection of each compound (Figure 2c,d).

Hernandez et al. (2012) demonstrated that compounds with similar exact masses and t_R_ values could be successfully separated by the precise mass measurements at the decimal level [23]. Similarly, Roy-Lachapelle et al. (2015) successfully separated analytes with an *m*/*z* difference of 0.0364 Da using the Orbitrap MS at a resolution of 17,500 FWHM [9]. Therefore, the QTOF MS utilized in this study proved well-suited for accurately identifying analytes with minute differences in *m*/*z* and t_R_. Details of the individual pesticide analysis in the MS^1^ are presented in Table S1.

### 3.2. Reliable Differentiation of Isomeric Pesticides Using MS^2^ Analysis and Spectral Library Database Construction for the Target Pesticides

**Figure 3:**
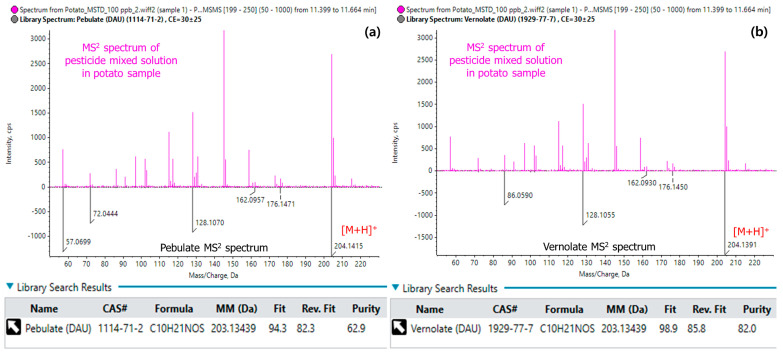
MS^2^ spectra of the isomeric pesticides (**a**) pebulate and (**b**) vernolate (black-colored), those of the mixed-pesticide solution in the potato sample (red-colored), and the Fit score results showing reliable differentiation using the spectral library database.

Additional MS^2^ analyses provide a more reliable approach for chemical identification based on the monoisotopic mass [24]. Mass spectral interference occurs when two species with identical molecular formulas coelute in the column simultaneously. These isomeric compounds can be distinguished by obtaining a structure-specific ion spectrum at the MS^2^ level, using either product ion scans or MRM [25,26]. In this study, pebulate and vernolate were distinguished using this strategy. The theoretical *m*/*z* values for the precursor ions ([M+H]^+^) of both structural isomers (C_10_H_21_NOSH^+^) were identical at *m*/*z* 204.14166, with the same t_R_ (11.25 min). The MS^1^ spectrum was insufficient for differentiation due to the overlap of the isotopic peaks. In contrast, distinct product ions were observed in the *m*/*z* range of 50–90 for the two analytes at the MS^2^ level (Figure 3). Nuñez et al. (2021) also utilized differences in the *m*/*z* values of 57, 72, and 86 in the *m*/*z* values for these two pesticides using a Q-Orbitrap, achieving successful differentiation [10].

After registering this spectral information in the library database, SWATH analysis of the pesticide multiresidue mixture in crop samples produced high Fit scores of 94.3 and 98.9 (out of 100) for pebulate and vernolate, respectively, confirming successful qualitative differentiation. Using the MS^2^ data, additional MRM transitions can be constructed and used for individual quantitative analysis [26,27]. For each of the 504 pesticides, the library database was constructed by acquiring MS^2^ spectra with a CE spread between 5 to 55 V. It allows for the acquisition of a more comprehensive set of fragment and precursor ions for each compound compared to using a single CE. By capturing both high- and low-energy fragments, different fragmentation patterns are produced simultaneously. This ensures that the library database contains a rich and diverse spectral profile, which enhances the reliability of the compound matching and differentiation during subsequent analyses [28]. The constructed in-house library database is expected to be applicable for suspect and non-target analyses.

### 3.3. Method Validation in the Representative Crops

The established analytical method using UHPLC-QTOF was validated for the multiresidue analysis of 504 pesticides across the following five representative crops: potato, cabbage, mandarin, brown rice, and soybean. These crops represent diverse matrices—starch-rich (potato), high in dietary fiber and pigments (cabbage), rich in organic acids (mandarin), carbohydrate-dense with low moisture (brown rice), and high in proteins and fats (soybean)—which pose analytical challenges. This diversity necessitates a robust analytical method capable of handling various matrices. Validation parameters included the limit of quantification (LOQ), linear range and linearity (*r*^2^) of calibration curves, and recovery at fortification levels of 0.01 and 0.1 µg/kg. The validation results for individual pesticides are summarized in Table 2 and Table S2.

#### 3.3.1. LOQ

The LOQ was determined as the minimum concentration of the matrix-matched standard that achieved an S/N of ≥10. The observed LOQ range for the target analytes in the five crops was 2.5–25 µg/kg (Table 2 and Table 3). Among these, 404–440 compounds had an LOQ of 2.5 µg/kg, indicating that over 80% of the analytes demonstrated the highest sensitivity in the analytical method. The sensitivity verified in our method is comparable to or greater than those achieved in recent studies using TQ MS in the MRM mode for the multiresidue analysis of over 100 compounds [15,29,30,31]. Unlike TQ MS, which requires the time-consuming establishment of individual MRM conditions for each pesticide, QTOF MS enables high-selectivity analysis at the full-spectrum data acquisition (MS^1^) level, thereby saving time.

Additionally, 488–498 (96.8–98.8%) compounds showed an LOQ of ≤10 µg/kg (Table 3), meeting the LOQ requirement of 10 µg/kg (0.01 mg/kg) for the study of pesticide residue, as stipulated by the FAO guidelines [32]. Thus, QTOF MS was confirmed to be suitable for trace-level analysis. Sivaperumal et al. (2015) achieved LOQs below 11.8 µg/kg for 60 pesticides in fruits and vegetables using UPLC-QTOF [33]. In our study, the target analytes were expanded to over 500 pesticides. This comprehensive approach allows for the simultaneous monitoring of a much wider range of pesticides, which improves the detection of potential contaminants in agricultural products. The inclusion of a larger number of compounds also increases the applicability of the analytical method, making it more valuable for regulatory compliance and food safety assessments.

#### 3.3.2. Linearity of Calibration Curve

The linearity of the calibration curve was assessed using the correlation coefficient (*r*^2^). In the representative crops, the target pesticides exhibited *r*^2^ greater than 0.940 (Table S2 and Table 4). Among these, 392 to 429 compounds (77.8% to 85.1%) demonstrated excellent linearity with *r*^2^ ≥ 0.990 (Table 4). Furthermore, 501 to 504 compounds achieved linearity, with *r*^2^ ≥ 0.980, meeting the linearity requirements for multiresidue analytical methods [34,35]. Good linearity in the QTOF MS indicates that the instrument provides consistent and reliable quantification across the concentration range for a large number of analytes. This is particularly important in multiresidue analysis because it ensures accurate quantification of trace-level pesticides in various matrices [36,37]. The high linearity reflects the ability of QTOF MS to produce reproducible ionization and detection of compounds over a wide dynamic range.

Certain compounds such as carbendazim, carbofuran-3-hydroxy, GPTC (isofetamid metabolite), NC 20645 (ethofumesate metabolite), imazapic, and phorate oxon sulfoxide exhibited slightly lower linearity, with *r*^2^ values ranging from 0.94 to 0.98 in some crops (Table S2). These compounds are relatively polar pesticides, making them more susceptible to matrix effects, such as ion suppression caused by co-eluting matrix components [38,39]. These factors can contribute to deviations from linearity in the calibration curve. To address these issues, further optimization of chromatographic conditions or sample preparation methods may be necessary for these polar pesticides. Additionally, incorporating appropriate internal standards can help compensate for linearities and improve the quantification accuracy for these challenging compounds [40].

#### 3.3.3. Recovery

The accuracy and precision of the analytical method were evaluated through recovery tests (Table 2). In multiresidue methods for crops, individual pesticide recoveries are typically confirmed at fortification levels of 10 and 100 µg/kg [4,41,42]. Among the five representative crops, four—potato, cabbage, mandarin, and brown rice—had 437 to 476 analytes achieving recovery rates between 70% and 120%, with relative standard deviations (RSDs) below 20%, thereby meeting the acceptable accuracy and precision ranges specified in the SANTE guidelines (Table 5) [20]. In soybean, 394 to 434 components (78.2–86.1%) met these criteria. These results demonstrate that the developed analytical method is robust and reliable across different crop matrices, ensuring accurate quantification of a wide range of pesticides. The high percentage of pesticides meeting the acceptable recovery range in most crops underscores the method’s effectiveness.

**Figure 4:**
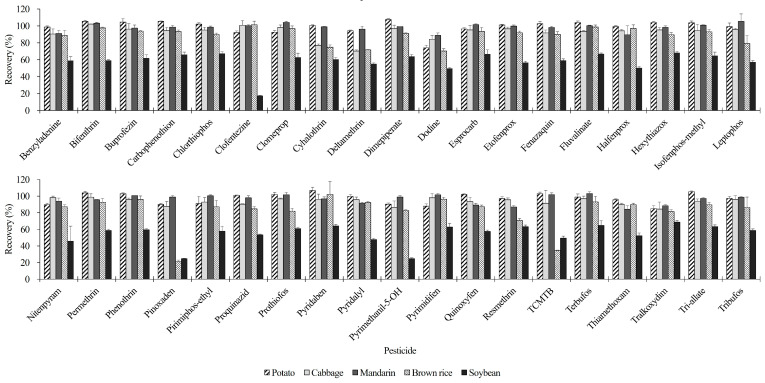
Recovery rates (100 µg/kg) of different pesticides across various crop matrices, showing those with recoveries below 70% in soybean, whereas generally acceptable in other crops.

A slightly lower number of satisfactory analytes were observed in soybean. Pesticides with recoveries below 70% in soybean—while generally acceptable in other crops—were predominantly nonpolar pesticides with high log *p*-values, such as pyrethroids and aliphatic organophosphates (Figure 4). This result may be attributed to the strong affinity between these pesticides and the lipid-rich matrix of soybean. Non-polar pesticides tend to adsorb into the fat components of the crop, resulting in reduced extraction efficiency [43,44]. These findings highlight the need for further method optimization or additional clean-up steps when analyzing high-fat samples like soybean to ensure accurate quantification of non-polar pesticides [44,45].

Pesticides containing acidic moieties, such as carboxylic acids or sulfonamides, as well as those belonging to the triketone class, exhibited recovery rates below 70% across all crops (Table S3). This reduction in recovery is attributed to the use of PSA during the sample clean-up step. PSA is known for effectively removing organic fatty acids and sugars, which can lead to the adsorption of these acidic pesticides onto the sorbent material, thereby decreasing their recovery rates [46,47]. Therefore, when analyzing pesticides from these categories, it is advisable to omit PSA from the clean-up process [45,48]. The results indicate that the observed low recoveries are due to the sample preparation procedure rather than the TOF MS system.

The SANTE guidelines allow for a broader recovery range of 30–140% for screening purposes, provided that the reproducibility is consistent with RSDs below 20% [20]. Applying these criteria, 452–494 pesticides—accounting for over 90% of the analytes—met these expanded recovery and precision standards across all representative crops. These findings reinforce the applicability of the analytical method for comprehensive and rapid pesticide screening, making it suitable for on-site use.

### 3.4. Application of the QTOF Method Using SWATH Analysis

The established analytical method was successfully applied to 42 crop samples obtained from distribution channels, including 7 potatoes, 4 cabbages, 4 mandarins, 26 rice, and 1 bean. Utilizing SWATH analysis, the MS^1^ and MS^2^ analyses were conducted simultaneously at HRMS levels, thereby enhancing qualitative performance [49]. Selectivity in MS^1^ was improved by maintaining mass accuracy within ±5 ppm. In MS^2^, the Fit score was evaluated through library searching, which enhances compound identification accuracy by ensuring that fragmentation patterns closely match those in the spectral library.

Pesticides were determined in one cabbage, three mandarins, and six rice samples (Table 6). The Fit scores ranged from 70.8 to 100, surpassing the acceptable threshold of 70 points [50,51]. Notably, up to seven pesticides were identified in a single sample, highlighting the value of multiresidue analysis for its ability to detect multiple contaminants in a single run. The residual amounts were significantly lower than the MRLs for all detected pesticides in crops. In conclusion, the QTOF multiresidue method utilizing SWATH analysis rapidly detected and identified hundreds of pesticide residues in food samples with high sensitivity and reliability. This method provides a reliable tool for ensuring food safety and monitoring pesticide residues in various agricultural products.

## 4. Conclusions

This study successfully developed a comprehensive multiresidue pesticide analysis method for 504 target analytes in five representative crops using UHPLC-QTOF. The high-mass accuracy achieved at the MS^1^ level confirmed the method’s suitability for pesticide analysis at trace levels, while the spectral library built from the MS^2^ data further enhanced the reliability of the compound identification. The validation results show excellent sensitivity, linearity, and recovery rates, meeting international standards for pesticide residue analysis. The application of this method to real agricultural samples underscored its effectiveness in detecting pesticide multiresidues simultaneously, making it a valuable tool for food safety monitoring and regulatory compliance.

## Figures and Tables

**Table 1:** Comparison of the theoretical and measured *m*/*z* values for benthiavalicarb-isopropyl with different isotopic substitutions and resolution details.

No.	Isotopic Species	Molecular Formula	Theoretical *m*/*z* [M+H]^+^	Resolution (FWHM ^1^)	Measured *m*/*z* [M+H]^+^	Mass Accuracy ^2^ (ppm)
1	Monoisotopic species	^12^C_18_H_24_FN_3_O_3_^32^S	382.15952	30,000 ^3^	382.15981	+0.76
				42,000 ^3^	382.15953	+0.03
2	Carbon-13, singly substituted	^12^C_17_^13^C_1_H_24_FN_3_O_3_^32^S	383.16287	30,000	383.16281	–0.16
				42,000	383.16329	+1.10
3	Sulfur-34, singly substituted	^12^C_18_H_24_FN_3_O_3_^34^S	384.15532	30,000	384.15898 ^4^	–18.87
				42,000	384.15978 ^4^	–16.79
4	Carbon-13, doubly substituted	^12^C_16_^13^C_2_H_24_FN_3_O_3_^32^S	384.16623	30,000	384.15898 ^4^	+9.53
				42,000	384.15978 ^4^	+11.61

^1^ Full width at half maximum. ^2^ (Mass accuracy) = [(theoretical *m*/*z*) − (measured *m*/*z*)]/(theoretical *m*/*z*) × 10^6^. ^3^ 30,000 FWHM was measured using an LCMS-9030 and 42,000 FWHM using an X500R. ^4^ The peaks of isotopes No. 3 and 4 in the mass spectra were not separated in both resolutions.

**Table 2:** Limits of quantification (LOQs) and recovery rates at 10 µg/kg and 100 µg/kg for 504 pesticide multiresidues in five representative crops using UHPLC-QTOF.

No.	Compound Name	Potato			Cabbage			Mandarin			Brown Rice			Soybean		
		LOQ (μg/kg)	Recovery, % (RSD ^1^, %)		LOQ (μg/kg)	Recovery, % (RSD, %)		LOQ (μg/kg)	Recovery, % (RSD, %)		LOQ (μg/kg)	Recovery, % (RSD, %)		LOQ (μg/kg)	Recovery, % (RSD, %)	
			10 μg/kg	100 μg/kg		10 μg/kg	100 μg/kg		10 μg/kg	100 μg/kg		10 μg/kg	100 μg/kg		10 μg/kg	100 μg/kg
1	2,3,5-Trimethacarb	2.5	93.2 (7.8)	105.3 (1.0)	2.5	102.7 (2.5)	97.6 (5.9)	10.0	87.1 (7.1)	99.7 (0.2)	2.5	101.6 (4.0)	105.1 (3.5)	2.5	100.3 (1.6)	103.9 (2.6)
2	3,4,5-Trimethacarb	2.5	98.7 (1.3)	109.2 (3.0)	2.5	100.7 (1.0)	87.2 (4.5)	5.0	100.0 (5.3)	106.0 (1.4)	2.5	106.5 (4.0)	106.5 (0.9)	2.5	98.4 (2.3)	91.1 (1.1)
3	Acetamiprid	5.0	83.7 (3.9)	96.3 (0.9)	2.5	107.4 (2.2)	87.2 (6.7)	2.5	104.3 (2.8)	90.6 (12.5)	2.5	106.2 (5.5)	96.0 (1.7)	2.5	96.9 (1.5)	87.1 (3.9)
4	Acetochlor	2.5	115.2 (3.9)	109.6 (2.3)	2.5	88.2 (2.2)	99.9 (2.6)	2.5	107.8 (6.7)	99.3 (1.1)	2.5	94.2 (4.7)	101.8 (1.7)	2.5	110.3 (1.8)	96.7 (1.0)
5	Acibenzolar acid	2.5	41.6 (50.1)	36.6 (32.3)	2.5	19.7 (6.3)	41.5 (76.0)	2.5	17.0 (13.2)	36.4 (19.5)	2.5	19.5 (47.2)	30.7 (25.8)	5.0	7.4 (116.8)	21.2 (42.8)
6	Acibenzolar-S-methyl	2.5	100.5 (4.7)	107.5 (0.8)	2.5	96.8 (3.8)	95.4 (2.8)	2.5	99.3 (2.0)	101.1 (0.7)	2.5	95.3 (2.3)	99.1 (1.3)	2.5	77.7 (2.0)	83.2 (3.9)
7	Acrinathrin	2.5	82.9 (1.5)	108.5 (3.7)	2.5	77.0 (3.4)	93.3 (3.7)	2.5	74.7 (0.3)	97.4 (1.7)	2.5	85.3 (17.2)	84.5 (1.6)	2.5	62.3 (0.7)	83.3 (2.2)
8	AD-67 (MON-4660)	2.5	94.5 (4.2)	110.4 (2.2)	2.5	108.1 (4.2)	98.8 (7.1)	2.5	97.7 (5.0)	95.3 (3.9)	2.5	107.3 (3.4)	104.4 (1.8)	2.5	97.9 (4.1)	98.3 (2.1)
9	Alachlor	2.5	110.1 (4.0)	109.6 (2.3)	2.5	97.6 (1.9)	99.9 (2.5)	2.5	96.0 (6.6)	99.1 (1.1)	2.5	92.1 (4.9)	99.1 (5.9)	2.5	110.3 (1.7)	96.7 (1.0)
10	Aldoxycarb (aldicarb sulfone)	2.5	76.5 (9.2)	93.3 (1.3)	5.0	92.4 (3.8)	95.9 (3.8)	5.0	95.4 (6.9)	87.9 (2.1)	5.0	120.9 (3.0)	101.1 (4.2)	2.5	100.8 (4.2)	94.4 (2.0)
11	Allethrin	2.5	102.7 (10.8)	104.4 (1.1)	2.5	97.7 (3.7)	97.1 (1.8)	2.5	104.6 (1.1)	101.7 (0.5)	2.5	95.7 (8.5)	100.6 (4.0)	2.5	71.2 (5.5)	88.1 (4.5)
12	Allidochlor	2.5	98.0 (7.5)	104.5 (2.6)	2.5	115.2 (4.1)	101.7 (3.6)	2.5	95.9 (0.8)	86.8 (4.8)	2.5	95.7 (3.6)	102.5 (2.0)	2.5	104.4 (7.1)	97.5 (3.5)
13	Ametoctradin	2.5	80.2 (4.3)	108.3 (1.9)	2.5	103.4 (3.9)	96.9 (5.8)	2.5	103.7 (2.7)	87.0 (0.4)	2.5	101.6 (1.1)	99.6 (5.4)	2.5	91.3 (2.2)	97.9 (1.1)
14	Ametryn	2.5	110.8 (1.8)	106.8 (2.9)	2.5	87.9 (3.8)	92.6 (19.7)	2.5	102.7 (1.7)	103.0 (0.9)	2.5	88.5 (2.8)	100.3 (0.8)	2.5	109.0 (1.4)	97.0 (2.8)
15	Amisulbrom	2.5	103.9 (3.3)	105.8 (1.7)	2.5	99.8 (1.4)	95.8 (4.7)	2.5	97.3 (3.8)	100.4 (1.3)	2.5	97.8 (5.0)	101.5 (2.5)	2.5	92.6 (3.8)	95.6 (2.2)
16	Anilofos	2.5	96.9 (2.5)	110.8 (1.0)	2.5	106.9 (1.6)	99.6 (5.7)	2.5	90.0 (1.4)	101.6 (1.3)	2.5	108.0 (2.1)	95.0 (1.3)	2.5	90.3 (2.7)	96.0 (2.3)
17	Aramite	2.5	109.8 (20.2)	97.9 (6.7)	5.0	77.3 (4.1)	95.0 (4.8)	2.5	109.6 (0.8)	103.0 (0.6)	2.5	104.2 (2.0)	94.3 (5.2)	2.5	90.3 (2.0)	71.2 (6.1)
18	Aspon	2.5	109.2 (2.2)	108.0 (0.8)	2.5	84.4 (4.5)	96.3 (3.5)	2.5	91.3 (0.6)	101.7 (0.5)	2.5	84.3 (3.3)	98.7 (1.9)	2.5	74.6 (1.2)	75.8 (2.5)
19	Asulam	2.5	19.5 (81.3)	42.1 (25.6)	10.0	50.1 (7.3)	52.6 (61.9)	10.0	N.D. ^2^	48.6 (10.0)	5.0	N.D.	49.1 (13.7)	2.5	39.4 (12.4)	49.0 (25.1)
20	Atrazine	2.5	110.7 (16.2)	94.9 (1.8)	5.0	92.6 (1.8)	90.0 (3.7)	2.5	102.9 (3.4)	96.1 (0.8)	5.0	89.2 (5.3)	92.2 (1.3)	2.5	111.1 (1.3)	84.9 (3.1)
21	Avermectin B1a	2.5	98.6 (2.2)	103.6 (1.0)	2.5	94.0 (3.6)	90.1 (4.5)	5.0	94.8 (2.5)	100.6 (3.6)	2.5	95.1 (12.9)	99.3 (1.5)	10.0	112.7 (6.1)	99.1 (2.7)
22	Azaconazole	2.5	105.8 (11.5)	99.1 (0.3)	2.5	108.0 (1.9)	100.8 (2.6)	2.5	84.1 (1.5)	89.7 (2.9)	2.5	109.5 (3.7)	80.1 (1.9)	2.5	102.9 (1.4)	100.4 (1.1)
23	Azamethiphos	2.5	97.2 (2.6)	106.7 (2.0)	2.5	101.9 (2.3)	98.8 (4.6)	2.5	102.8 (0.4)	92.3 (1.2)	2.5	95.0 (3.0)	105.8 (0.5)	2.5	86.7 (0.9)	102.1 (3.2)
24	Azimsulfuron	2.5	86.3 (10.4)	84.3 (9.2)	2.5	76.2 (4.0)	83.3 (13.3)	2.5	73.3 (1.2)	80.8 (6.7)	2.5	86.8 (4.5)	84.5 (3.1)	2.5	71.3 (2.8)	85.4 (8.3)
25	Azinphos-ethyl	2.5	102.2 (4.0)	97.3 (1.4)	2.5	86.8 (3.5)	87.7 (3.7)	2.5	91.0 (0.5)	92.1 (1.0)	5.0	97.4 (4.9)	94.0 (1.7)	2.5	107.2 (3.1)	85.9 (0.1)
26	Azoxystrobin	2.5	93.0 (6.4)	109.5 (0.7)	2.5	102.1 (0.9)	96.7 (2.0)	2.5	102.2 (3.3)	104.0 (1.6)	2.5	104.7 (3.1)	98.1 (2.3)	2.5	100.1 (1.1)	94.6 (0.7)
27	Bendiocarb	2.5	92.8 (4.8)	94.8 (1.5)	20.0	N.D.	86.1 (4.8)	2.5	106.5 (1.8)	93.1 (0.8)	2.5	104.0 (5.7)	96.1 (0.7)	2.5	102.1 (2.4)	93.0 (2.4)
28	Benfuresate	2.5	113.3 (2.1)	99.4 (1.1)	2.5	106.2 (3.8)	91.2 (2.7)	2.5	108.9 (2.8)	92.5 (2.8)	2.5	109.0 (4.4)	96.5 (1.6)	2.5	107.7 (0.9)	105.0 (1.5)
29	Benodanil	5.0	89.6 (2.2)	97.6 (0.7)	2.5	90.9 (2.7)	89.2 (4.0)	2.5	91.5 (0.2)	90.3 (2.0)	5.0	105.9 (4.0)	91.8 (3.0)	2.5	104.0 (1.1)	90.2 (1.9)
30	Bensulide	2.5	96.7 (2.0)	110.0 (1.7)	2.5	99.6 (1.7)	99.4 (3.5)	2.5	104.1 (1.4)	100.1 (1.1)	2.5	108.9 (2.7)	103.7 (3.2)	5.0	108.5 (0.1)	104.1 (2.3)
31	Benthiavalicarb-isopropyl	2.5	97.1 (4.6)	112.8 (1.1)	2.5	99.9 (0.6)	97.1 (0.9)	2.5	102.7 (1.2)	101.7 (0.9)	2.5	102.4 (3.4)	99.9 (3.5)	2.5	101.7 (1.3)	96.0 (0.7)
32	Benzobicyclon	2.5	95.5 (7.3)	99.9 (5.8)	2.5	77.7 (3.1)	87.7 (11.2)	2.5	91.7 (1.3)	92.3 (4.2)	2.5	86.9 (11.1)	81.4 (12.8)	2.5	89.7 (1.6)	99.2 (2.2)
33	Benzoximate	2.5	97.9 (6.6)	101.6 (1.6)	2.5	102.6 (1.7)	98.3 (5.4)	2.5	88.7 (2.4)	101.1 (0.8)	2.5	102.3 (3.1)	95.8 (1.4)	5.0	55.2 (4.2)	74.3 (7.2)
34	Benzoylprop-ethyl	2.5	107.8 (11.3)	98.0 (1.8)	2.5	94.2 (2.1)	100.6 (5.2)	20.0	N.D.	105.3 (1.0)	2.5	100.6 (4.5)	101.1 (3.0)	2.5	108.6 (3.1)	88.4 (2.1)
35	Benzpyrimoxan	2.5	102.1 (4.7)	92.6 (2.7)	5.0	102.6 (3.9)	89.5 (6.1)	2.5	91.9 (1.4)	89.0 (1.7)	2.5	76.4 (4.9)	104.0 (1.2)	2.5	102.2 (2.6)	85.7 (1.0)
36	Benzyladenine	2.5	103.7 (6.3)	98.7 (1.2)	2.5	95.7 (7.6)	90.4 (6.5)	2.5	96.0 (5.8)	90.9 (4.1)	2.5	90.6 (5.9)	88.6 (6.8)	5.0	50.5 (7.3)	59.0 (7.9)
37	Bifenox	2.5	111.0 (2.7)	96.0 (2.2)	2.5	110.3 (3.7)	89.3 (2.8)	10.0	70.7 (24.0)	106.7 (3.6)	2.5	96.6 (12.1)	98.7 (0.3)	5.0	74.8 (8.1)	86.7 (6.2)
38	Bifenthrin	5.0	97.4 (3.4)	105.3 (1.2)	2.5	108.6 (8.4)	102.1 (0.2)	2.5	101.2 (4.4)	103.2 (1.1)	2.5	108.1 (9.9)	97.6 (1.0)	2.5	38.6 (5.0)	59.1 (1.9)
39	Bispyribac	2.5	68.8 (83.5)	46.4 (42.8)	2.5	5.1 (116.9)	87.2 (138.3)	10.0	N.D.	31.8 (29.9)	2.5	36.0 (40.0)	33.8 (47.3)	5.0	N.D.	34.4 (59.1)
40	Bistrifluron	2.5	110.6 (1.0)	105.9 (0.5)	2.5	108.6 (2.0)	103.3 (1.5)	2.5	94.9 (1.6)	89.8 (2.3)	2.5	97.7 (13.6)	104.7 (3.4)	2.5	96.9 (0.8)	103.9 (3.0)
41	Bitertanol	2.5	105.1 (3.9)	107.0 (1.8)	2.5	93.6 (1.2)	97.2 (1.4)	2.5	98.5 (2.1)	98.3 (0.5)	2.5	101.2 (2.3)	105.1 (2.7)	5.0	113.7 (0.6)	117.4 (3.5)
42	Bixafen	5.0	121.0 (2.2)	95.8 (1.0)	2.5	107.1 (2.5)	86.8 (3.6)	2.5	110.7 (4.7)	90.2 (2.4)	5.0	113.7 (2.2)	97.9 (2.4)	5.0	114.1 (1.5)	99.7 (1.8)
43	Boscalid	2.5	102.7 (3.4)	98.9 (1.7)	5.0	92.2 (3.3)	93.1 (1.6)	2.5	84.1 (2.8)	94.6 (1.6)	5.0	97.1 (2.9)	93.1 (0.9)	5.0	98.5 (1.4)	95.3 (1.6)
44	Bromacil	2.5	109.6 (3.5)	101.7 (2.9)	2.5	104.6 (3.2)	100.2 (3.7)	2.5	102.6 (4.2)	104.0 (3.0)	2.5	100.1 (2.6)	105.5 (4.2)	2.5	103.0 (11.3)	99.1 (5.1)
45	Bromobutide	5.0	101.5 (12.9)	97.3 (2.2)	2.5	121.5 (2.7)	87.8 (3.5)	2.5	102.2 (2.7)	84.3 (2.6)	2.5	96.5 (4.9)	92.6 (1.4)	2.5	109.3 (1.9)	85.2 (1.7)
46	Bupirimate	2.5	116.1 (3.9)	95.1 (2.8)	2.5	98.4 (2.5)	86.1 (1.9)	2.5	107.6 (3.3)	99.0 (0.8)	2.5	108.1 (2.5)	96.4 (0.6)	5.0	113.5 (1.4)	88.4 (3.9)
47	Buprofezin	2.5	115.1 (5.3)	104.5 (3.7)	2.5	84.0 (6.8)	96.2 (6.5)	2.5	71.8 (12.0)	97.4 (3.8)	2.5	110.4 (3.3)	93.9 (0.9)	2.5	67.0 (2.9)	62.1 (6.2)
48	Butachlor	2.5	93.4 (14.3)	104.3 (2.9)	2.5	102.4 (3.8)	97.1 (4.4)	5.0	94.1 (0.7)	100.9 (1.3)	2.5	92.2 (3.5)	94.3 (3.8)	2.5	72.7 (0.5)	72.2 (5.0)
49	Butafenacil	2.5	92.8 (3.8)	99.6 (3.5)	10.0	74.9 (7.6)	98.2 (4.5)	2.5	113.5 (1.5)	104.4 (0.9)	2.5	111.2 (3.8)	99.9 (2.0)	2.5	110.9 (0.9)	99.3 (1.3)
50	Butocarboxim	2.5	84.3 (3.4)	108.4 (1.0)	20.0	N.D.	96.4 (13.0)	2.5	102.0 (2.7)	85.0 (1.7)	20.0	N.D.	111.8 (4.2)	2.5	99.4 (1.1)	97.3 (4.5)
51	Butralin	2.5	107.0 (2.1)	108.5 (0.5)	2.5	93.3 (1.3)	97.7 (1.0)	2.5	85.2 (3.4)	96.0 (1.5)	2.5	110.7 (2.3)	91.4 (2.1)	2.5	84.8 (1.5)	77.9 (2.8)
52	Butylate	2.5	109.8 (1.2)	105.2 (1.2)	2.5	82.2 (1.2)	95.7 (2.1)	2.5	106.7 (1.8)	99.1 (0.4)	2.5	110.4 (1.4)	92.3 (2.1)	2.5	81.6 (3.3)	74.1 (2.7)
53	Cadusafos	2.5	116.1 (3.2)	97.3 (1.2)	2.5	85.2 (5.7)	89.5 (5.1)	2.5	94.4 (0.6)	103.6 (1.3)	2.5	95.1 (2.2)	104.2 (2.2)	2.5	107.5 (1.0)	95.2 (2.7)
54	Cafenstrole	2.5	103.3 (16.4)	108.5 (2.2)	2.5	106.1 (1.1)	99.9 (4.0)	2.5	92.4 (1.7)	95.2 (0.3)	2.5	74.8 (2.6)	98.9 (1.4)	5.0	110.2 (3.5)	94.8 (1.4)
55	Carbaryl	20.0	N.D.	108.7 (1.9)	2.5	95.3 (3.6)	99.8 (6.9)	20.0	N.D.	99.5 (4.5)	2.5	98.1 (5.1)	106.2 (4.8)	2.5	96.9 (1.7)	97.8 (3.2)
56	Carbendazim	2.5	110.5 (4.4)	109.6 (2.0)	2.5	97.1 (0.8)	99.8 (3.7)	20.0	N.D.	111.8 (6.6)	2.5	99.5 (4.9)	107.1 (3.1)	2.5	76.1 (6.8)	92.0 (4.8)
57	Carbetamide	2.5	107.2 (2.3)	108.7 (2.2)	2.5	97.2 (2.5)	97.7 (1.6)	2.5	109.0 (1.1)	94.3 (5.5)	2.5	100.6 (2.2)	105.9 (2.5)	2.5	85.7 (3.1)	92.5 (3.5)
58	Carbofuran	5.0	88.2 (5.3)	97.1 (1.9)	2.5	107.7 (0.9)	86.4 (6.4)	2.5	94.2 (3.9)	95.4 (0.3)	5.0	113.0 (3.4)	106.5 (0.9)	2.5	104.8 (1.5)	93.8 (2.2)
59	Carbofuran-3-hydroxy	5.0	103.6 (2.4)	96.8 (0.9)	2.5	102.1 (4.4)	101.8 (6.1)	5.0	90.2 (7.2)	106.7 (5.9)	2.5	102.7 (2.3)	99.3 (2.0)	2.5	99.3 (1.7)	92.8 (2.9)
60	Carbophenothion	2.5	94.3 (1.8)	105.3 (0.6)	2.5	100.1 (5.8)	94.4 (4.1)	2.5	98.5 (0.7)	98.5 (2.0)	2.5	79.1 (5.9)	93.3 (1.9)	5.0	59.7 (2.4)	66.3 (4.3)
61	Carboxin	2.5	73.2 (4.3)	90.5 (2.1)	2.5	86.1 (0.9)	100.2 (5.0)	2.5	76.7 (1.6)	98.9 (3.1)	2.5	78.2 (2.8)	98.8 (1.5)	25.0	N.D.	82.6 (3.9)
62	Carfentrazone-ethyl	2.5	84.7 (0.7)	93.3 (0.9)	2.5	106.2 (3.3)	100.9 (4.3)	2.5	87.2 (1.2)	103.8 (1.5)	2.5	85.2 (3.4)	87.9 (4.0)	2.5	97.3 (5.0)	104.2 (3.3)
63	Carpropamide	5.0	104.2 (3.9)	94.8 (1.2)	2.5	105.3 (0.7)	87.0 (4.9)	2.5	91.5 (0.2)	103.6 (1.2)	5.0	98.2 (2.4)	95.1 (3.3)	5.0	82.9 (1.6)	83.2 (1.9)
64	Chlorantraniliprole	2.5	88.0 (1.9)	94.3 (1.3)	2.5	110.2 (7.0)	93.2 (1.6)	2.5	87.7 (3.4)	99.1 (2.0)	2.5	107.1 (4.1)	105.3 (1.0)	2.5	106.1 (0.6)	106.8 (1.3)
65	Chlorbufam	2.5	109.9 (6.7)	96.1 (1.6)	5.0	85.2 (5.0)	83.4 (27.9)	20.0	N.D.	97.5 (6.9)	5.0	113.3 (18.6)	96.3 (1.0)	10.0	89.4 (7.6)	94.4 (5.1)
66	Chlorfenapyr	10.0	104.0 (10.7)	103.4 (4.0)	5.0	125.3 (6.3)	80.5 (21.0)	5.0	77.7 (5.6)	80.3 (2.8)	5.0	118.6 (9.5)	96.5 (3.9)	2.5	74.0 (10.8)	76.2 (5.5)
67	Chlorfenvinphos	2.5	86.9 (4.4)	93.4 (1.7)	2.5	101.8 (1.2)	99.0 (4.2)	2.5	94.2 (1.7)	103.1 (0.6)	2.5	101.6 (2.0)	96.7 (3.8)	2.5	96.0 (1.9)	99.7 (2.6)
68	Chlorfluazuron	2.5	94.1 (0.5)	76.9 (1.3)	2.5	94.7 (1.5)	96.8 (2.1)	2.5	92.9 (0.8)	96.9 (1.7)	5.0	89.1 (5.2)	98.2 (2.0)	2.5	73.6 (2.6)	80.3 (2.7)
69	Chlorflurenol-methyl	5.0	131.7 (4.2)	99.7 (2.0)	10.0	113.5 (2.5)	91.6 (16.6)	5.0	64.9 (4.4)	78.7 (1.2)	2.5	110.0 (2.8)	97.9 (1.7)	5.0	121.6 (1.8)	96.2 (3.6)
70	Chloridazon	5.0	100.2 (1.2)	94.7 (1.0)	2.5	107.1 (1.9)	85.0 (8.2)	2.5	91.7 (2.0)	86.1 (4.0)	5.0	91.6 (3.2)	92.9 (1.2)	5.0	71.2 (2.2)	78.0 (4.4)
71	Chlorimuron-ethyl	2.5	94.5 (20.2)	80.0 (13.0)	2.5	73.8 (3.9)	77.0 (29.4)	2.5	78.4 (0.7)	74.6 (4.2)	2.5	88.2 (11.3)	77.1 (7.2)	2.5	78.1 (3.7)	82.3 (16.0)
72	Chlorobenzuron	5.0	105.9 (0.9)	94.3 (2.1)	2.5	114.3 (2.7)	101.1 (4.3)	2.5	95.2 (12.0)	96.0 (3.3)	2.5	95.0 (2.5)	97.3 (3.7)	2.5	73.4 (5.0)	75.9 (2.8)
73	Chlorotoluron	2.5	95.1 (5.1)	107.9 (2.6)	2.5	107.8 (2.1)	95.4 (6.2)	2.5	95.1 (2.4)	107.0 (2.4)	2.5	105.5 (2.8)	94.9 (4.1)	2.5	104.5 (1.7)	109.4 (3.3)
74	Chloroxuron	2.5	107.1 (2.0)	93.5 (1.3)	2.5	103.6 (2.8)	91.9 (2.8)	2.5	89.6 (2.1)	105.4 (0.8)	2.5	105.3 (2.3)	96.0 (2.8)	5.0	90.6 (0.9)	87.5 (2.9)
75	Chlorpyrifos	2.5	111.7 (2.4)	100.7 (2.5)	2.5	108.6 (2.3)	93.4 (2.3)	2.5	82.7 (0.8)	98.4 (1.4)	2.5	103.5 (4.4)	89.0 (1.4)	2.5	73.6 (2.8)	66.9 (3.0)
76	Chlorpyrifos-methyl	2.5	110.6 (1.2)	103.0 (0.8)	2.5	93.7 (3.7)	93.7 (2.0)	2.5	87.3 (3.0)	98.8 (1.7)	2.5	106.3 (4.3)	92.7 (3.2)	2.5	83.1 (1.1)	76.4 (2.6)
77	Chlorthiophos	2.5	81.3 (1.3)	102.0 (1.5)	2.5	87.8 (3.2)	95.2 (3.4)	2.5	82.5 (2.6)	98.5 (1.4)	2.5	76.4 (6.9)	89.7 (2.1)	2.5	55.2 (0.7)	67.5 (2.8)
78	Chromafenozide	2.5	90.9 (4.2)	96.1 (0.4)	2.5	103.3 (2.5)	95.0 (3.6)	2.5	89.8 (3.7)	104.3 (0.9)	2.5	105.4 (4.0)	98.5 (1.6)	5.0	94.9 (1.2)	92.4 (1.4)
79	Cinmethylin	2.5	100.7 (2.2)	103.0 (1.7)	2.5	84.0 (14.9)	96.6 (5.7)	10.0	77.3 (8.7)	97.8 (1.2)	2.5	118.3 (5.9)	90.1 (2.4)	5.0	53.9 (28.1)	70.5 (5.3)
80	Clethodim	2.5	76.6 (10.0)	83.0 (6.1)	2.5	77.5 (1.1)	86.5 (13.1)	2.5	78.8 (1.5)	86.1 (1.5)	2.5	74.5 (3.1)	81.3 (4.4)	2.5	67.1 (1.0)	75.8 (4.8)
81	Clethodim sulfone	2.5	88.7 (17.2)	79.3 (11.6)	5.0	64.8 (6.9)	75.4 (28.9)	2.5	73.0 (1.9)	78.6 (2.8)	2.5	76.4 (1.8)	80.8 (6.7)	2.5	80.1 (2.5)	83.3 (9.4)
82	Clethodim sulfoxide	2.5	64.4 (30.5)	57.7 (16.8)	2.5	41.5 (5.4)	42.5 (9.4)	2.5	42.0 (12.8)	51.1 (7.0)	2.5	52.3 (6.5)	63.7 (7.0)	2.5	49.5 (6.6)	61.4 (22.0)
83	Clofentezine	2.5	98.3 (2.3)	92.0 (2.3)	2.5	100.1 (2.3)	100.6 (5.6)	2.5	102.5 (3.3)	100.1 (1.3)	2.5	96.4 (3.4)	101.3 (4.1)	2.5	14.6 (3.9)	17.5 (2.1)
84	Clomazone	2.5	110.1 (3.9)	97.3 (0.9)	2.5	104.1 (1.2)	87.1 (4.8)	2.5	86.5 (2.0)	97.7 (1.3)	2.5	109.4 (3.9)	94.6 (1.4)	2.5	100.0 (0.4)	89.9 (0.7)
85	Clomeprop	5.0	92.8 (7.2)	92.6 (2.2)	2.5	109.3 (3.3)	98.0 (3.2)	2.5	83.0 (0.3)	104.0 (1.3)	2.5	91.1 (0.9)	96.7 (3.4)	2.5	59.4 (2.2)	62.9 (7.8)
86	Coumaphos	2.5	105.3 (0.7)	94.2 (3.3)	5.0	92.0 (2.3)	91.4 (3.5)	2.5	99.1 (2.1)	101.5 (1.2)	5.0	105.0 (4.9)	93.1 (3.0)	5.0	108.2 (1.3)	89.6 (2.2)
87	Crufomate	5.0	103.4 (2.0)	93.7 (1.4)	2.5	102.1 (1.5)	86.5 (2.3)	2.5	95.6 (2.6)	105.2 (2.1)	2.5	107.4 (4.4)	92.2 (0.9)	2.5	103.2 (2.4)	92.4 (1.7)
88	Cyanazine	2.5	99.6 (4.0)	108.0 (5.5)	2.5	94.8 (1.6)	97.0 (1.9)	2.5	95.7 (3.1)	109.0 (3.2)	2.5	97.9 (7.4)	96.4 (3.3)	2.5	78.4 (8.3)	84.9 (2.3)
89	Cyanophos	2.5	137.5 (6.3)	92.7 (4.0)	2.5	134.7 (4.8)	101.7 (3.8)	10.0	86.5 (11.6)	107.8 (4.2)	2.5	115.1 (12.1)	104.9 (5.9)	5.0	111.4 (6.0)	90.1 (3.3)
90	Cyantraniliprole	2.5	103.7 (3.3)	104.9 (0.4)	2.5	99.1 (0.6)	98.3 (2.3)	2.5	102.3 (1.1)	97.3 (0.7)	2.5	99.8 (4.0)	99.6 (2.1)	2.5	100.3 (2.5)	103.3 (1.4)
91	Cyazofamid	2.5	91.6 (3.0)	93.4 (1.8)	2.5	106.8 (1.8)	85.2 (3.3)	2.5	95.2 (1.7)	102.4 (1.0)	5.0	104.4 (3.7)	92.5 (2.2)	2.5	107.2 (1.8)	91.6 (0.6)
92	Cyclaniliprole	2.5	90.8 (1.4)	108.3 (2.1)	2.5	102.7 (1.2)	99.0 (3.1)	2.5	89.6 (0.4)	104.6 (1.8)	5.0	106.7 (3.5)	98.7 (0.9)	2.5	102.4 (2.3)	97.7 (1.9)
93	Cycloate	2.5	97.9 (3.1)	106.9 (1.0)	2.5	97.7 (1.4)	95.2 (3.6)	2.5	100.7 (1.1)	99.5 (1.7)	2.5	93.4 (1.5)	92.6 (2.6)	5.0	66.0 (2.0)	71.0 (1.6)
94	Cycloprothrin	2.5	98.6 (0.6)	106.0 (1.1)	2.5	94.0 (2.5)	95.7 (2.2)	2.5	98.0 (3.5)	99.1 (0.9)	2.5	98.9 (4.1)	98.4 (1.0)	2.5	74.3 (2.5)	83.5 (1.5)
95	Cyclosulfamuron	2.5	96.1 (24.6)	89.4 (14.3)	2.5	70.5 (3.2)	77.8 (17.2)	2.5	71.7 (0.9)	81.4 (3.9)	2.5	90.1 (11.0)	83.6 (5.5)	2.5	72.2 (5.9)	84.9 (9.3)
96	Cyenopyrafen	2.5	95.8 (0.7)	106.6 (0.6)	2.5	93.0 (2.8)	94.6 (2.7)	2.5	103.4 (1.3)	101.1 (0.9)	2.5	96.8 (1.3)	90.3 (1.7)	2.5	82.1 (2.7)	93.1 (2.3)
97	Cyflufenamid	2.5	93.1 (3.5)	90.9 (2.0)	5.0	88.8 (3.0)	90.3 (2.8)	2.5	94.7 (0.6)	91.2 (1.4)	5.0	116.9 (4.3)	95.3 (2.8)	2.5	104.0 (1.3)	84.8 (5.1)
98	Cyflumetofen	2.5	104.8 (6.5)	105.3 (4.2)	5.0	99.0 (6.4)	91.2 (4.8)	2.5	99.4 (1.0)	100.9 (2.6)	5.0	76.7 (27.2)	78.4 (17.3)	10.0	72.2 (1.3)	94.6 (6.8)
99	Cyhalofop-butyl	20.0	N.D.	104.1 (0.6)	2.5	106.8 (5.0)	93.0 (0.7)	5.0	97.7 (2.5)	102.2 (1.7)	2.5	100.9 (10.8)	101.3 (4.6)	5.0	78.8 (5.8)	92.0 (2.0)
100	Cyhalothrin	2.5	60.6 (0.5)	100.5 (0.7)	2.5	62.4 (2.5)	76.9 (2.3)	2.5	61.9 (3.9)	98.9 (0.5)	5.0	63.9 (6.1)	74.8 (3.6)	2.5	44.6 (6.6)	60.6 (3.3)
101	Cymoxanil	2.5	96.7 (22.5)	102.8 (5.5)	2.5	102.0 (0.8)	97.0 (3.9)	10.0	93.4 (2.0)	91.1 (2.1)	2.5	97.9 (5.3)	100.7 (3.3)	5.0	86.8 (2.6)	97.2 (3.7)
102	Cyprazine	2.5	102.6 (8.4)	90.9 (2.0)	5.0	114.4 (2.8)	87.3 (4.7)	2.5	94.3 (3.3)	90.8 (2.3)	2.5	90.9 (5.9)	91.9 (0.6)	2.5	104.0 (0.9)	83.5 (2.7)
103	Cyproconazole	2.5	97.5 (3.2)	95.4 (1.0)	2.5	102.4 (2.8)	94.0 (2.7)	2.5	89.0 (3.2)	102.7 (0.8)	2.5	104.1 (4.9)	93.2 (1.6)	2.5	98.1 (2.4)	89.7 (2.1)
104	Cyprodinil	2.5	101.8 (2.6)	102.3 (2.3)	2.5	113.5 (2.5)	96.9 (5.9)	2.5	81.4 (2.8)	99.7 (0.8)	2.5	105.3 (1.8)	93.5 (2.4)	2.5	79.8 (2.0)	79.4 (3.6)
105	Cyromazine	2.5	36.9 (18.1)	50.0 (2.9)	5.0	52.5 (24.1)	60.3 (7.0)	10.0	37.8 (20.4)	39.3 (1.9)	2.5	26.1 (15.6)	34.1 (10.6)	5.0	N.D.	20.4 (11.0)
106	Daimuron (dymron)	2.5	100.3 (13.0)	85.4 (4.3)	2.5	105.7 (1.7)	85.2 (4.1)	2.5	88.0 (1.3)	103.2 (1.8)	2.5	113.0 (3.7)	92.1 (2.4)	5.0	90.2 (2.3)	86.5 (0.5)
107	Deltamethrin	2.5	66.6 (1.6)	94.2 (0.9)	2.5	86.9 (4.5)	70.3 (1.9)	2.5	92.6 (5.5)	96.2 (3.3)	10.0	94.2 (7.5)	71.6 (0.7)	2.5	60.9 (10.0)	55.2 (1.8)
108	Demeton-S	2.5	103.7 (3.5)	106.8 (1.0)	2.5	103.2 (2.7)	93.4 (5.1)	2.5	105.9 (3.8)	103.5 (3.2)	2.5	99.2 (3.7)	101.9 (2.8)	2.5	96.5 (2.1)	101.6 (2.0)
109	Demeton-S sulfone	2.5	106.8 (2.5)	96.3 (2.2)	5.0	94.7 (2.3)	102.0 (6.8)	2.5	91.5 (2.1)	106.0 (1.6)	5.0	106.1 (2.7)	100.2 (0.4)	2.5	104.0 (0.9)	99.3 (2.2)
110	Demeton-S sulfoxide	2.5	102.7 (13.9)	82.7 (0.4)	5.0	81.8 (2.0)	112.7 (26.2)	2.5	105.9 (6.1)	82.2 (4.4)	5.0	106.2 (6.0)	99.3 (5.0)	2.5	100.1 (1.7)	97.0 (3.1)
111	Demeton-S-methyl	20.0	N.D.	109.7 (1.2)	20.0	N.D.	98.8 (5.9)	20.0	N.D.	97.2 (2.8)	20.0	N.D.	106.3 (1.6)	25.0	N.D.	86.6 (2.8)
112	Demeton-S-methylsulfone	2.5	105.1 (4.4)	95.0 (1.1)	2.5	105.8 (0.4)	88.6 (5.2)	2.5	90.5 (3.7)	100.8 (3.0)	2.5	108.1 (3.3)	94.8 (2.9)	2.5	85.8 (1.0)	95.0 (2.6)
113	Desmetryn	2.5	103.3 (2.7)	104.1 (0.8)	2.5	116.9 (1.3)	97.8 (5.4)	2.5	85.6 (1.1)	99.6 (3.0)	2.5	87.2 (5.4)	99.9 (0.3)	2.5	104.9 (1.8)	96.9 (2.0)
114	Dialifor	2.5	93.5 (3.8)	92.6 (0.9)	5.0	77.9 (5.4)	85.3 (4.5)	2.5	97.4 (2.5)	89.4 (1.0)	2.5	114.9 (1.0)	83.5 (2.1)	2.5	106.3 (0.8)	89.5 (1.5)
115	Di-allate	2.5	99.1 (2.7)	102.6 (2.1)	2.5	105.7 (4.7)	94.9 (3.2)	2.5	101.8 (2.2)	97.0 (1.1)	2.5	100.2 (1.8)	89.9 (2.2)	2.5	70.3 (1.8)	71.8 (3.0)
116	Diazinon	2.5	114.9 (3.3)	102.7 (0.4)	2.5	109.6 (2.5)	95.9 (6.2)	2.5	88.2 (2.3)	101.2 (0.7)	2.5	110.9 (3.3)	95.1 (2.8)	2.5	89.8 (0.9)	86.0 (2.8)
117	Dichlobenil	5.0	109.9 (3.5)	97.4 (2.5)	10.0	103.4 (1.3)	98.9 (5.7)	10.0	86.8 (12.9)	102.1 (0.9)	10.0	75.5 (3.7)	91.7 (7.2)	5.0	89.9 (1.3)	86.7 (3.3)
118	Dichlofenthion	2.5	114.5 (1.5)	100.3 (4.8)	2.5	93.5 (8.5)	97.2 (1.5)	2.5	90.2 (6.9)	100.2 (3.4)	2.5	106.7 (7.1)	92.2 (3.9)	2.5	71.8 (5.3)	73.6 (3.1)
119	Dichlormid	2.5	104.6 (2.2)	106.1 (1.8)	2.5	82.5 (0.8)	98.0 (4.2)	2.5	96.5 (4.6)	103.5 (0.8)	2.5	118.2 (5.9)	104.3 (3.1)	2.5	104.7 (2.2)	96.6 (2.8)
120	Dichlorvos	20.0	N.D.	109.0 (0.4)	2.5	80.9 (3.4)	99.1 (5.6)	2.5	93.7 (1.3)	103.2 (3.1)	20.0	N.D.	84.6 (1.2)	25.0	N.D.	86.1 (2.2)
121	Diclobutrazol	2.5	121.5 (3.3)	102.1 (1.7)	5.0	78.9 (2.8)	92.1 (3.1)	2.5	100.8 (1.6)	100.8 (1.2)	5.0	104.0 (2.8)	97.4 (4.7)	2.5	103.4 (3.6)	93.4 (4.3)
122	Diclocymet	5.0	104.0 (2.5)	95.0 (1.5)	5.0	88.2 (2.3)	89.2 (3.6)	5.0	99.2 (1.6)	93.9 (0.7)	5.0	84.5 (3.5)	94.4 (2.4)	2.5	105.9 (1.0)	87.9 (1.4)
123	Diclofop-methyl	20.0	N.D.	92.8 (1.6)	5.0	113.4 (12.8)	101.1 (4.2)	10.0	71.2 (13.7)	108.2 (0.8)	5.0	86.1 (34.9)	101.7 (8.5)	2.5	60.4 (3.2)	70.6 (7.9)
124	Diclosulam	2.5	72.6 (24.3)	77.8 (17.1)	2.5	55.9 (6.0)	71.5 (27.2)	2.5	66.1 (5.6)	78.5 (6.8)	5.0	64.2 (19.0)	73.6 (4.6)	2.5	61.0 (5.4)	76.0 (11.8)
125	Dicrotophos	2.5	94.9 (4.5)	86.6 (2.8)	5.0	82.3 (1.6)	89.7 (7.2)	2.5	102.6 (8.0)	96.0 (1.9)	5.0	117.5 (4.5)	97.8 (7.5)	2.5	102.4 (0.6)	92.0 (3.6)
126	Dicyclanil	2.5	119.5 (11.2)	103.1 (12.9)	2.5	112.2 (1.0)	93.8 (8.0)	2.5	105.4 (22.7)	84.2 (8.4)	2.5	90.2 (9.1)	86.6 (18.3)	2.5	74.4 (1.1)	62.0 (4.4)
127	Diethatyl-ethyl	2.5	105.1 (1.9)	92.6 (2.0)	2.5	76.5 (4.9)	89.1 (4.5)	2.5	88.2 (3.8)	72.5 (2.0)	2.5	81.5 (3.5)	93.4 (2.3)	2.5	113.0 (1.6)	90.7 (2.9)
128	Diethofencarb	20.0	N.D.	93.6 (0.9)	5.0	104.8 (4.9)	92.4 (5.5)	2.5	93.3 (0.8)	93.0 (0.9)	2.5	89.0 (2.1)	91.9 (1.3)	5.0	112.1 (2.5)	93.6 (0.8)
129	Difenoconazole	2.5	110.7 (5.2)	94.1 (0.8)	5.0	75.2 (4.1)	92.5 (3.2)	2.5	90.3 (3.9)	100.8 (1.4)	5.0	111.3 (2.3)	92.1 (3.2)	2.5	109.4 (0.5)	98.2 (1.7)
130	Diflubenzuron	2.5	93.2 (3.2)	81.9 (1.6)	5.0	98.4 (3.1)	91.4 (3.6)	2.5	87.2 (8.5)	104.8 (1.2)	2.5	105.2 (2.1)	93.5 (2.9)	2.5	103.1 (1.7)	89.5 (1.9)
131	Diflufenican	2.5	100.9 (1.8)	96.9 (2.1)	5.0	109.5 (1.6)	93.6 (3.0)	2.5	99.4 (0.6)	100.7 (0.8)	2.5	106.6 (3.3)	89.9 (1.4)	2.5	95.0 (2.1)	89.1 (2.2)
132	Dimepiperate	2.5	99.1 (2.7)	107.6 (1.0)	2.5	100.3 (3.6)	96.7 (4.2)	2.5	101.2 (1.1)	99.0 (0.2)	2.5	93.4 (3.9)	90.9 (1.1)	5.0	59.7 (2.5)	63.9 (3.4)
133	Dimethametryn	2.5	109.2 (4.9)	104.1 (2.0)	2.5	102.1 (2.1)	94.3 (5.3)	2.5	104.6 (1.4)	98.0 (2.5)	2.5	99.3 (3.8)	96.1 (1.9)	2.5	82.6 (1.0)	85.3 (3.9)
134	Dimethenamid	2.5	100.6 (7.6)	90.8 (3.0)	2.5	88.4 (1.6)	85.4 (8.2)	2.5	97.3 (6.5)	91.7 (1.6)	2.5	95.6 (3.6)	92.8 (3.1)	2.5	110.6 (1.7)	86.2 (3.4)
135	Dimethoate	2.5	87.0 (6.2)	98.8 (1.4)	5.0	106.5 (3.0)	91.6 (8.8)	2.5	91.5 (2.0)	87.5 (5.6)	5.0	112.2 (3.7)	101.2 (3.0)	5.0	96.2 (1.4)	93.8 (3.0)
136	Dimethylvinphos	5.0	80.8 (4.3)	95.6 (1.8)	2.5	77.3 (4.0)	87.0 (3.5)	2.5	77.5 (4.2)	101.9 (2.5)	2.5	111.7 (2.7)	94.4 (1.1)	2.5	103.9 (1.9)	89.0 (1.6)
137	Dinitramine	2.5	85.9 (3.8)	101.5 (0.7)	2.5	88.0 (4.5)	99.8 (2.9)	2.5	88.8 (1.3)	102.1 (1.4)	2.5	87.3 (8.1)	97.0 (2.7)	2.5	94.7 (1.3)	83.6 (3.8)
138	Dioxathion	2.5	110.6 (11.1)	90.4 (1.3)	5.0	99.3 (8.2)	83.9 (2.7)	2.5	110.3 (4.8)	85.8 (3.8)	5.0	113.1 (7.1)	99.8 (3.5)	25.0	N.D.	78.5 (4.1)
139	Diphenamid	2.5	99.1 (2.4)	94.3 (2.1)	2.5	108.3 (3.0)	78.8 (22.8)	2.5	91.3 (2.1)	103.6 (0.5)	2.5	112.8 (2.2)	94.9 (0.4)	2.5	105.3 (1.1)	91.5 (2.8)
140	Disulfoton sulfone	2.5	89.7 (1.4)	97.4 (1.3)	2.5	105.4 (1.8)	90.1 (4.6)	2.5	90.9 (0.5)	110.0 (1.9)	5.0	103.9 (3.1)	97.6 (2.8)	5.0	106.6 (0.6)	103.0 (0.6)
141	Disulfoton sulfoxide	2.5	111.0 (4.8)	99.9 (2.0)	2.5	106.5 (2.7)	89.6 (3.7)	2.5	94.3 (3.4)	93.3 (0.2)	5.0	105.0 (4.8)	100.0 (1.2)	2.5	113.0 (1.5)	103.7 (1.8)
142	Dithiopyr	2.5	103.8 (2.3)	104.5 (2.6)	2.5	89.8 (6.1)	86.0 (15.8)	2.5	90.3 (2.3)	101.6 (0.9)	2.5	112.5 (5.5)	98.2 (2.4)	2.5	103.1 (2.2)	93.8 (2.8)
143	Diuron	5.0	104.4 (4.3)	94.4 (1.8)	2.5	80.2 (1.7)	85.4 (4.1)	2.5	93.1 (1.3)	91.0 (2.0)	5.0	101.9 (4.2)	90.8 (1.9)	2.5	104.1 (1.0)	87.2 (1.1)
144	DNOC	2.5	88.8 (10.5)	89.8 (6.3)	2.5	84.1 (0.8)	92.3 (7.8)	2.5	90.7 (1.0)	96.3 (1.7)	2.5	81.5 (4.6)	85.0 (3.4)	2.5	80.5 (1.8)	89.0 (3.9)
145	Dodine	2.5	113.6 (1.8)	74.1 (4.1)	5.0	126.2 (1.7)	84.1 (5.6)	5.0	112.7 (1.4)	88.8 (3.3)	5.0	98.2 (3.6)	70.2 (4.2)	2.5	66.0 (2.5)	49.8 (2.1)
146	Edifenphos	5.0	86.7 (1.5)	91.6 (1.3)	5.0	76.1 (6.0)	87.0 (5.8)	2.5	97.7 (2.3)	89.1 (2.0)	5.0	96.0 (3.1)	89.8 (4.0)	2.5	87.2 (3.3)	91.8 (2.3)
147	Emamectin B1a	2.5	93.9 (1.4)	107.4 (1.2)	2.5	87.3 (2.8)	96.7 (6.7)	2.5	93.3 (1.2)	100.9 (0.4)	2.5	87.7 (2.0)	100.9 (2.7)	2.5	74.8 (1.3)	94.0 (3.0)
148	Epoxiconazole	2.5	95.8 (2.1)	92.4 (2.2)	5.0	105.9 (1.3)	88.6 (3.1)	2.5	86.2 (1.3)	104.2 (1.0)	5.0	97.7 (1.6)	94.4 (1.5)	5.0	109.3 (1.7)	93.4 (1.4)
149	EPTC	2.5	107.4 (4.3)	104.3 (1.3)	2.5	83.7 (1.9)	96.3 (2.1)	2.5	90.5 (2.0)	100.1 (1.8)	2.5	102.6 (2.7)	93.6 (2.4)	2.5	82.5 (3.7)	79.1 (1.6)
150	Esprocarb	2.5	99.8 (8.8)	96.3 (2.0)	2.5	105.1 (3.5)	95.4 (5.0)	2.5	107.4 (2.9)	101.5 (0.8)	2.5	100.5 (3.8)	93.5 (4.9)	2.5	55.3 (5.4)	66.7 (7.6)
151	Etaconazole	2.5	113.1 (1.4)	95.4 (0.9)	2.5	101.1 (1.7)	87.5 (3.0)	2.5	92.8 (3.7)	105.2 (0.8)	2.5	113.5 (2.4)	93.3 (1.0)	2.5	103.9 (1.1)	92.1 (2.3)
152	Ethaboxam	2.5	96.7 (2.4)	95.4 (0.8)	5.0	110.0 (1.4)	90.6 (2.5)	2.5	94.1 (2.3)	105.1 (0.8)	5.0	112.4 (2.6)	94.6 (2.2)	5.0	108.2 (0.2)	103.9 (1.7)
153	Ethametsulfuron-methyl	2.5	66.5 (34.9)	61.7 (24.9)	2.5	41.7 (6.7)	63.0 (45.3)	5.0	42.9 (4.2)	62.4 (6.8)	2.5	65.2 (25.3)	68.2 (12.3)	2.5	48.6 (8.1)	69.8 (26.8)
154	Ethiofencarb	2.5	105.9 (3.2)	104.9 (2.0)	2.5	104.9 (3.6)	89.8 (3.7)	5.0	65.6 (14.2)	102.8 (0.7)	2.5	102.4 (6.0)	99.4 (2.2)	2.5	95.7 (2.0)	94.5 (2.9)
155	Ethion	2.5	96.0 (2.5)	107.1 (0.2)	2.5	105.8 (4.3)	94.8 (5.0)	2.5	91.6 (1.6)	97.2 (0.6)	2.5	85.3 (2.1)	96.8 (2.4)	2.5	71.0 (1.2)	76.7 (2.4)
156	Ethofumesate	2.5	102.7 (0.8)	95.9 (1.4)	2.5	76.0 (5.1)	99.0 (4.8)	2.5	81.3 (2.1)	104.7 (1.6)	2.5	80.2 (5.3)	103.6 (2.1)	2.5	113.2 (2.8)	105.0 (3.5)
157	Ethofumesate metabolite (NC 20645)	2.5	87.0 (4.8)	91.8 (0.8)	2.5	93.2 (2.0)	112.4 (26.0)	2.5	99.8 (5.9)	82.6 (4.5)	2.5	104.4 (2.3)	83.2 (8.2)	2.5	97.2 (2.1)	96.6 (3.2)
158	Ethoprophos (ethoprop)	5.0	100.3 (5.5)	94.2 (2.4)	2.5	91.8 (1.7)	84.0 (2.5)	2.5	92.5 (1.7)	93.3 (0.9)	2.5	96.3 (2.3)	91.8 (0.2)	2.5	114.5 (1.8)	87.0 (2.6)
159	Ethychlozate	2.5	91.4 (2.6)	107.7 (1.5)	2.5	101.9 (2.5)	99.4 (2.7)	2.5	102.8 (2.0)	104.0 (2.8)	2.5	106.6 (3.9)	92.1 (0.8)	25.0	N.D.	84.7 (1.9)
160	Etofenprox	2.5	89.7 (2.2)	101.1 (0.8)	2.5	93.2 (2.9)	96.6 (1.9)	2.5	97.3 (0.4)	99.8 (1.2)	2.5	90.9 (4.0)	91.9 (2.1)	2.5	47.3 (1.3)	56.7 (2.7)
161	Etrimfos	2.5	105.1 (4.3)	107.9 (1.2)	2.5	101.1 (0.7)	97.7 (5.1)	2.5	104.8 (1.8)	101.2 (1.9)	2.5	99.9 (3.0)	92.6 (3.9)	2.5	83.3 (1.2)	90.0 (1.3)
162	Famoxadone	2.5	94.9 (2.6)	107.7 (2.1)	5.0	92.0 (0.9)	98.1 (4.7)	2.5	95.7 (3.7)	103.0 (1.8)	2.5	98.4 (3.0)	102.0 (3.7)	2.5	98.9 (2.2)	103.3 (1.6)
163	Fenamidone	2.5	104.9 (7.0)	112.3 (1.7)	2.5	101.1 (2.0)	101.9 (3.1)	2.5	89.9 (2.0)	99.9 (2.1)	2.5	113.1 (2.3)	90.6 (3.7)	2.5	95.8 (2.2)	99.7 (1.9)
164	Fenamiphos	2.5	92.5 (0.7)	90.4 (1.5)	2.5	106.2 (2.4)	89.1 (3.3)	2.5	91.8 (2.8)	105.8 (1.5)	5.0	104.7 (2.9)	94.4 (1.1)	2.5	106.6 (2.7)	94.9 (1.9)
165	Fenarimol	2.5	107.7 (0.2)	103.2 (1.0)	2.5	99.9 (3.6)	94.9 (3.7)	2.5	104.7 (1.7)	100.6 (0.8)	2.5	107.1 (0.5)	91.5 (2.1)	2.5	96.8 (3.0)	93.0 (2.7)
166	Fenazaquin	2.5	100.7 (2.4)	102.5 (2.8)	2.5	98.7 (3.7)	91.7 (3.6)	2.5	99.2 (0.3)	98.1 (1.5)	2.5	89.9 (11.1)	89.9 (3.5)	2.5	48.4 (4.5)	59.1 (3.2)
167	Fenbuconazole	2.5	95.7 (1.4)	88.8 (3.1)	2.5	103.7 (2.8)	97.4 (2.6)	2.5	93.3 (4.0)	100.7 (2.1)	2.5	109.9 (3.0)	96.6 (2.1)	2.5	110.3 (2.5)	105.2 (1.9)
168	Fenfuram	2.5	99.4 (2.8)	92.3 (0.9)	2.5	88.6 (2.1)	98.4 (3.8)	2.5	83.8 (2.2)	95.3 (1.8)	2.5	90.4 (6.8)	97.3 (1.9)	2.5	105.2 (1.2)	100.9 (3.4)
169	Fenhexamid	2.5	93.9 (9.4)	94.3 (4.6)	2.5	83.3 (1.7)	88.9 (7.7)	2.5	97.0 (2.6)	95.8 (0.5)	2.5	94.8 (3.0)	93.5 (1.5)	2.5	80.3 (2.9)	86.5 (3.1)
170	Fenobucarb	2.5	97.8 (5.3)	94.7 (1.7)	2.5	104.4 (4.4)	89.7 (6.4)	2.5	86.5 (1.6)	104.2 (0.4)	2.5	85.0 (4.8)	102.7 (1.5)	2.5	106.9 (2.3)	104.4 (1.6)
171	Fenothiocarb	2.5	101.0 (3.4)	93.2 (1.9)	2.5	105.8 (2.4)	88.4 (4.5)	2.5	86.0 (0.4)	102.0 (1.7)	2.5	105.5 (3.1)	90.9 (2.3)	2.5	92.5 (2.0)	96.1 (2.6)
172	Fenoxanil	2.5	100.0 (1.9)	93.7 (0.7)	2.5	104.3 (3.6)	89.9 (3.5)	2.5	95.9 (2.1)	92.1 (1.2)	2.5	82.4 (3.4)	102.3 (1.3)	2.5	106.6 (3.1)	90.2 (2.0)
173	Fenoxaprop-ethyl	2.5	62.2 (2.2)	88.1 (1.6)	2.5	107.9 (3.2)	98.8 (3.7)	2.5	92.5 (0.8)	93.0 (1.0)	2.5	102.4 (1.5)	90.6 (2.1)	2.5	87.2 (1.1)	93.9 (2.5)
174	Fenoxycarb	2.5	108.6 (1.8)	103.4 (0.7)	2.5	109.3 (1.8)	99.6 (3.7)	2.5	83.3 (1.5)	102.8 (1.0)	2.5	108.6 (2.9)	91.5 (3.4)	2.5	101.0 (1.9)	91.4 (1.0)
175	Fenpropathrin	2.5	109.2 (0.6)	104.4 (1.3)	2.5	107.5 (3.1)	97.9 (1.8)	2.5	83.6 (0.9)	98.9 (1.2)	2.5	105.8 (2.4)	96.8 (1.9)	2.5	76.9 (1.4)	80.2 (2.5)
176	Fenpropimorph	2.5	83.6 (6.0)	102.8 (1.2)	2.5	108.2 (2.9)	96.6 (6.0)	2.5	82.6 (1.8)	101.3 (1.0)	2.5	104.4 (2.4)	93.5 (2.1)	2.5	76.1 (0.9)	75.6 (0.3)
177	Fenpyrazamine	2.5	89.4 (4.2)	86.4 (3.6)	2.5	104.0 (1.9)	84.9 (3.5)	2.5	91.5 (3.6)	100.0 (1.4)	2.5	111.4 (5.0)	95.3 (3.3)	2.5	100.4 (1.1)	101.5 (2.4)
178	Fenpyroximate	2.5	103.0 (1.4)	107.4 (0.5)	2.5	97.3 (2.4)	96.9 (1.8)	2.5	102.4 (1.1)	102.7 (1.8)	2.5	94.0 (3.0)	95.3 (1.1)	2.5	75.7 (1.5)	87.1 (2.4)
179	Fensulfothion	5.0	88.8 (1.7)	97.2 (1.1)	2.5	103.7 (1.1)	88.0 (1.4)	2.5	90.8 (1.4)	91.6 (1.8)	2.5	111.1 (4.4)	94.2 (1.4)	5.0	103.1 (1.4)	96.8 (1.8)
180	Fenthion	2.5	97.7 (3.2)	91.4 (1.6)	2.5	106.1 (1.2)	88.9 (3.0)	2.5	80.4 (0.7)	85.9 (1.2)	2.5	106.7 (5.0)	86.3 (3.5)	2.5	92.5 (2.0)	88.3 (1.7)
181	Fentrazamide	2.5	91.3 (3.6)	93.0 (0.8)	2.5	106.5 (2.0)	90.0 (4.5)	2.5	85.4 (2.6)	100.1 (2.6)	2.5	112.5 (2.9)	93.5 (1.7)	2.5	96.3 (1.9)	102.0 (3.6)
182	Ferimzone	2.5	97.7 (5.4)	93.9 (1.6)	2.5	106.2 (1.8)	97.7 (4.7)	2.5	102.4 (1.7)	91.5 (3.5)	2.5	105.7 (2.2)	95.6 (1.2)	2.5	102.1 (1.0)	106.3 (1.2)
183	Fipronil	5.0	108.6 (3.5)	94.5 (1.5)	5.0	98.1 (7.9)	95.5 (4.2)	20.0	N.D.	85.8 (4.8)	2.5	114.2 (15.1)	91.9 (6.3)	5.0	107.3 (6.7)	92.6 (1.0)
184	Flamprop-isopropyl	2.5	110.3 (2.5)	93.4 (2.8)	2.5	84.7 (1.3)	89.6 (3.0)	2.5	88.3 (1.0)	102.1 (0.7)	2.5	93.4 (2.5)	92.0 (2.9)	2.5	110.2 (2.6)	89.8 (1.7)
185	Flazasulfuron	2.5	77.1 (26.5)	61.3 (20.0)	2.5	55.9 (6.7)	60.7 (36.6)	2.5	65.1 (1.6)	63.1 (5.4)	5.0	72.7 (24.1)	62.9 (8.6)	2.5	55.9 (8.2)	62.5 (23.5)
186	Flonicamid	2.5	104.8 (2.0)	106.2 (0.5)	2.5	108.4 (3.4)	102.0 (2.6)	2.5	95.0 (6.8)	99.9 (2.7)	2.5	104.7 (3.9)	105.3 (1.2)	5.0	63.4 (1.5)	74.1 (3.2)
187	Florpyrauxifen	2.5	32.0 (70.9)	20.4 (45.2)	2.5	9.3 (95.7)	24.3 (112.9)	10.0	23.7 (21.1)	16.8 (36.7)	2.5	14.0 (63.8)	21.1 (48.8)	10.0	39.2 (13.6)	16.4 (50.3)
188	Fluacrypyrim	2.5	107.8 (2.3)	109.8 (0.5)	2.5	99.8 (3.3)	98.5 (5.1)	2.5	103.0 (1.8)	100.2 (1.3)	2.5	106.6 (1.2)	105.2 (1.8)	2.5	92.1 (2.1)	97.0 (1.9)
189	Fluazifop Butyl	2.5	68.2 (4.1)	96.2 (3.1)	2.5	102.4 (2.7)	96.0 (4.3)	2.5	105.8 (1.3)	100.9 (0.9)	2.5	107.3 (1.4)	99.5 (2.8)	2.5	89.9 (1.6)	97.5 (2.0)
190	Fluazinam	2.5	94.1 (1.3)	101.3 (1.0)	2.5	89.7 (2.2)	98.3 (0.3)	2.5	100.9 (0.7)	103.2 (2.5)	2.5	92.6 (3.1)	97.7 (0.9)	2.5	85.7 (0.6)	92.8 (2.5)
191	Flubendiamide	2.5	92.8 (4.0)	102.7 (1.8)	2.5	98.1 (4.1)	88.2 (0.5)	2.5	84.9 (2.2)	85.7 (0.7)	5.0	112.2 (4.2)	100.4 (0.9)	2.5	124.3 (2.5)	113.8 (0.7)
192	Flucetosulfuron	2.5	94.3 (15.7)	81.1 (11.9)	2.5	77.5 (3.8)	80.9 (30.6)	2.5	80.1 (4.7)	79.0 (6.0)	2.5	97.8 (9.0)	80.5 (5.9)	2.5	76.3 (1.9)	78.1 (11.4)
193	Flucythrinate	2.5	103.0 (1.3)	108.4 (0.8)	2.5	97.8 (2.7)	96.8 (2.1)	2.5	100.0 (0.8)	99.8 (0.7)	2.5	99.7 (3.1)	100.3 (2.4)	2.5	84.9 (2.1)	94.1 (0.9)
194	Fludioxonil	5.0	99.1 (6.4)	94.5 (2.3)	5.0	95.0 (2.1)	83.9 (9.4)	2.5	93.5 (1.3)	82.9 (3.1)	2.5	109.5 (3.8)	89.8 (3.1)	2.5	99.4 (2.3)	86.9 (2.4)
195	Flufenacet	5.0	106.9 (2.4)	92.6 (1.1)	5.0	98.7 (2.4)	87.5 (2.7)	2.5	88.6 (4.7)	83.6 (0.5)	5.0	109.2 (2.8)	96.9 (0.3)	2.5	104.0 (1.6)	89.3 (3.8)
196	Flufenoxuron	2.5	102.1 (2.5)	105.8 (0.4)	2.5	98.4 (3.6)	96.3 (2.5)	2.5	97.6 (2.7)	99.6 (1.5)	2.5	90.6 (4.4)	94.4 (6.1)	2.5	85.3 (1.9)	95.9 (1.7)
197	Flufenpyr-ethyl	20.0	N.D.	66.3 (1.4)	2.5	102.7 (1.6)	89.5 (2.5)	2.5	96.9 (1.3)	104.1 (1.0)	2.5	109.1 (2.0)	93.9 (2.3)	2.5	108.4 (1.1)	94.4 (2.1)
198	Flumetralin	2.5	91.6 (1.9)	104.6 (1.4)	5.0	96.4 (8.3)	100.6 (2.3)	10.0	92.7 (6.2)	101.0 (1.4)	20.0	N.D.	91.1 (2.7)	10.0	63.0 (9.0)	81.3 (0.2)
199	Flumioxazin	2.5	90.7 (1.5)	90.7 (1.6)	5.0	102.3 (1.7)	83.0 (40.9)	10.0	90.9 (1.8)	103.2 (1.5)	2.5	108.0 (1.0)	97.1 (1.3)	2.5	103.2 (2.0)	96.1 (1.0)
200	Fluometuron	5.0	103.3 (2.4)	96.8 (1.5)	2.5	105.9 (2.9)	85.4 (4.6)	2.5	83.7 (5.6)	84.7 (1.0)	2.5	108.6 (4.6)	90.5 (2.4)	2.5	106.4 (1.2)	95.5 (3.3)
201	Fluopicolide	2.5	86.3 (0.8)	95.7 (0.7)	2.5	101.7 (2.6)	87.3 (3.4)	2.5	87.9 (3.9)	93.2 (0.3)	2.5	107.8 (3.0)	93.4 (2.8)	2.5	100.1 (0.4)	90.2 (3.3)
202	Fluopyram	2.5	103.5 (4.6)	92.7 (0.9)	2.5	93.4 (3.1)	85.9 (4.2)	2.5	100.4 (2.2)	88.3 (0.2)	2.5	87.1 (5.2)	93.9 (1.7)	2.5	111.6 (1.1)	90.4 (1.7)
203	Flupoxam	2.5	106.8 (0.6)	107.6 (1.4)	2.5	97.5 (2.6)	97.0 (3.5)	2.5	102.9 (4.2)	99.8 (0.3)	2.5	103.7 (1.9)	100.2 (3.3)	2.5	107.1 (1.5)	106.3 (1.9)
204	Flupyradifurone	2.5	81.2 (2.1)	96.6 (0.4)	2.5	105.2 (3.5)	93.1 (2.8)	2.5	104.4 (4.9)	93.1 (9.3)	2.5	108.0 (2.0)	95.8 (1.7)	2.5	111.3 (2.0)	100.2 (1.1)
205	Fluquinconazole	2.5	87.9 (3.6)	96.3 (1.2)	2.5	105.6 (1.5)	98.8 (3.8)	2.5	77.1 (12.1)	102.2 (1.3)	2.5	113.7 (1.5)	100.5 (1.9)	2.5	101.0 (1.9)	98.1 (2.5)
206	Fluridone	2.5	86.2 (3.1)	92.0 (1.5)	2.5	106.8 (0.3)	88.6 (15.6)	2.5	96.7 (4.2)	103.6 (1.1)	2.5	106.1 (3.0)	92.6 (2.9)	2.5	101.1 (0.9)	89.8 (2.0)
207	Flurochloridone	2.5	102.6 (1.4)	95.5 (0.5)	2.5	104.5 (1.1)	87.0 (3.8)	2.5	97.9 (1.0)	105.0 (0.3)	2.5	92.8 (3.6)	92.4 (2.0)	2.5	101.9 (1.7)	86.8 (2.6)
208	Flurtamone	2.5	101.5 (3.3)	93.7 (1.8)	5.0	110.4 (4.2)	89.4 (6.8)	5.0	101.7 (2.5)	95.1 (1.5)	2.5	100.1 (3.1)	95.1 (2.0)	5.0	113.8 (2.0)	89.7 (0.5)
209	Flusilazole	2.5	111.3 (2.4)	91.9 (1.2)	2.5	92.6 (3.6)	86.9 (3.2)	2.5	101.9 (0.4)	92.2 (1.1)	2.5	89.5 (5.5)	92.4 (0.8)	2.5	110.5 (2.0)	89.0 (1.7)
210	Flusulfamide	2.5	96.1 (1.9)	91.0 (1.0)	5.0	98.9 (3.2)	92.7 (1.8)	2.5	92.1 (0.6)	97.4 (2.4)	2.5	106.8 (2.7)	106.0 (1.0)	2.5	80.7 (1.5)	114.1 (2.7)
211	Fluthiacet-methyl	20.0	N.D.	78.6 (2.4)	2.5	94.1 (2.0)	90.7 (3.7)	2.5	88.8 (1.7)	96.0 (5.9)	2.5	90.7 (0.6)	99.4 (5.1)	2.5	102.4 (2.2)	106.3 (1.4)
212	Flutianil	5.0	84.2 (3.2)	92.0 (1.7)	5.0	104.9 (2.6)	88.2 (4.3)	2.5	83.0 (0.9)	90.3 (2.8)	5.0	97.5 (3.2)	99.2 (3.3)	5.0	112.6 (2.3)	94.4 (2.0)
213	Flutolanil	5.0	101.6 (6.1)	91.1 (0.2)	5.0	98.1 (0.9)	86.5 (4.5)	2.5	92.5 (1.1)	86.9 (4.2)	5.0	105.8 (3.7)	96.6 (2.6)	5.0	100.8 (3.2)	90.4 (1.4)
214	Flutriafol	2.5	108.9 (2.2)	103.2 (2.3)	2.5	106.3 (2.6)	98.1 (1.1)	2.5	83.5 (3.4)	99.1 (0.8)	2.5	102.0 (4.4)	101.0 (3.0)	2.5	103.4 (1.1)	106.8 (0.5)
215	Fluvalinate	2.5	107.4 (1.2)	103.8 (1.8)	2.5	102.0 (3.5)	93.6 (1.0)	2.5	101.2 (4.1)	100.1 (0.7)	2.5	109.9 (3.2)	98.6 (2.6)	5.0	65.3 (4.2)	67.0 (1.5)
216	Fluxametamide	2.5	92.2 (2.7)	90.6 (0.8)	2.5	95.4 (1.9)	89.7 (0.4)	2.5	94.5 (2.2)	94.8 (2.6)	2.5	99.4 (12.4)	100.9 (1.5)	2.5	96.1 (2.6)	81.1 (3.6)
217	Fluxapyroxad	2.5	101.3 (2.7)	92.4 (1.5)	2.5	87.5 (4.1)	84.5 (4.1)	2.5	85.0 (3.0)	90.9 (1.4)	2.5	90.2 (4.1)	90.8 (2.6)	5.0	113.1 (1.3)	94.2 (2.4)
218	Fomesafen	2.5	90.5 (4.6)	90.2 (3.6)	2.5	84.9 (2.6)	86.3 (7.7)	2.5	87.5 (6.2)	93.7 (2.8)	2.5	86.8 (1.9)	89.2 (1.5)	2.5	88.7 (2.3)	92.4 (2.8)
219	Fonofos	2.5	104.2 (3.9)	107.6 (0.8)	2.5	100.7 (3.6)	97.4 (4.0)	2.5	100.9 (2.1)	99.4 (1.5)	2.5	96.7 (4.6)	94.7 (2.6)	2.5	75.2 (1.3)	77.5 (1.0)
220	Foramsulfuron	2.5	37.4 (40.7)	35.5 (29.0)	2.5	19.9 (15.5)	42.1 (71.4)	2.5	20.9 (2.3)	34.5 (17.6)	2.5	36.2 (32.4)	41.1 (17.2)	2.5	6.3 (23.4)	35.5 (37.8)
221	Forchlorfenuron	2.5	88.2 (4.3)	104.4 (2.7)	2.5	101.9 (3.3)	96.7 (1.9)	2.5	102.2 (3.2)	99.7 (2.2)	2.5	101.3 (3.2)	96.7 (1.9)	2.5	89.4 (1.3)	97.0 (2.1)
222	Fosthiazate	2.5	75.2 (3.8)	105.8 (1.0)	2.5	104.2 (1.0)	88.0 (3.8)	2.5	90.9 (2.6)	102.8 (0.9)	2.5	105.4 (3.8)	104.5 (1.6)	2.5	106.1 (1.4)	99.5 (2.9)
223	Furathiocarb	2.5	102.4 (2.4)	106.1 (3.3)	2.5	94.7 (2.1)	96.1 (4.3)	2.5	101.8 (0.7)	100.8 (0.1)	2.5	80.1 (1.3)	82.1 (2.5)	2.5	84.1 (1.6)	89.6 (2.3)
224	GPTC (isofetamid metabolite)	2.5	94.1 (11.9)	74.4 (7.6)	2.5	87.1 (11.6)	80.6 (11.0)	10.0	87.4 (5.3)	84.4 (2.1)	2.5	87.1 (20.5)	71.5 (19.0)	5.0	76.2 (2.4)	61.7 (20.6)
225	Halfenprox	2.5	91.4 (3.7)	99.0 (1.1)	2.5	92.9 (5.2)	94.2 (1.8)	2.5	86.9 (5.0)	89.4 (12.2)	2.5	88.0 (2.7)	97.0 (4.4)	2.5	40.5 (3.2)	50.3 (3.1)
226	Halosulfuron-methyl	2.5	86.3 (7.8)	93.4 (5.0)	2.5	79.6 (2.5)	91.3 (5.4)	2.5	80.1 (3.3)	93.0 (2.9)	2.5	77.9 (3.4)	90.8 (3.2)	2.5	78.9 (2.5)	96.9 (4.2)
227	Heptenophos	20.0	N.D.	87.4 (1.5)	2.5	92.0 (2.4)	88.1 (4.3)	2.5	82.5 (4.0)	104.5 (1.8)	2.5	91.0 (3.7)	101.6 (2.1)	2.5	83.8 (1.1)	91.3 (1.8)
228	Hexaconazole	2.5	79.6 (6.9)	72.4 (2.9)	2.5	106.8 (2.3)	98.8 (4.7)	2.5	106.9 (0.8)	102.0 (0.6)	2.5	104.2 (1.8)	102.5 (1.7)	2.5	106.2 (3.4)	104.9 (2.5)
229	Hexaflumuron	2.5	90.8 (5.1)	103.8 (2.8)	2.5	109.2 (3.2)	95.1 (3.7)	2.5	102.0 (2.0)	104.7 (1.3)	2.5	103.8 (9.1)	102.8 (0.7)	2.5	96.1 (1.2)	98.6 (3.1)
230	Hexazinone	5.0	103.5 (2.6)	92.5 (1.7)	5.0	101.2 (2.6)	87.9 (5.2)	2.5	109.3 (2.2)	92.4 (0.5)	2.5	109.7 (1.8)	104.0 (1.5)	2.5	101.4 (0.4)	88.6 (1.7)
231	Hexythiazox	2.5	105.7 (0.8)	103.8 (1.6)	2.5	101.5 (2.1)	95.2 (3.1)	2.5	102.2 (2.8)	98.4 (1.6)	2.5	97.1 (3.5)	89.4 (3.1)	5.0	66.0 (1.8)	68.2 (2.2)
232	Imazalil	2.5	99.8 (2.4)	100.5 (1.3)	2.5	106.3 (2.7)	95.6 (5.8)	2.5	105.2 (0.1)	102.1 (1.3)	2.5	95.7 (3.9)	98.0 (0.8)	2.5	74.4 (2.5)	80.8 (4.7)
233	Imazamethabenz-methyl	2.5	98.9 (4.0)	97.1 (1.5)	5.0	110.4 (0.8)	88.8 (4.8)	2.5	99.6 (0.5)	95.4 (1.8)	2.5	126.8 (3.6)	105.7 (1.1)	5.0	110.6 (0.0)	92.6 (3.9)
234	Imazamox	2.5	17.2 (86.7)	18.0 (40.4)	2.5	N.D.	23.3 (105.5)	2.5	3.2 (6.3)	17.7 (28.5)	2.5	9.6 (81.6)	21.6 (44.3)	2.5	N.D.	15.1 (57.4)
235	Imazamox metabolite (M720H001)	2.5	28.6 (16.5)	7.4 (37.7)	2.5	N.D.	12.3 (98.8)	20.0	N.D.	14.4 (17.3)	2.5	22.2 (6.4)	8.4 (42.8)	10.0	52.2 (16.0)	8.0 (24.1)
236	Imazapic	2.5	8.9 (86.8)	15.9 (37.5)	2.5	N.D.	22.6 (105.3)	2.5	N.D.	16.7 (27.4)	2.5	5.1 (86.8)	20.4 (44.3)	5.0	N.D.	10.7 (74.4)
237	Imazaquin	2.5	17.0 (85.6)	16.5 (46.1)	2.5	N.D.	22.7 (115.4)	2.5	N.D.	15.0 (37.3)	2.5	7.8 (86.6)	19.1 (53.5)	2.5	N.D.	13.7 (62.0)
238	Imazethapyr	2.5	8.3 (87.3)	21.2 (27.1)	2.5	0.9 (134.5)	26.1 (100.1)	2.5	N.D.	19.4 (29.1)	2.5	11.3 (64.5)	23.7 (43.4)	2.5	N.D.	11.4 (53.3)
239	Imazosulfuron	2.5	91.8 (22.2)	75.7 (16.9)	2.5	70.9 (2.7)	67.6 (29.7)	5.0	65.2 (2.8)	64.0 (7.4)	2.5	83.0 (12.9)	70.7 (9.9)	2.5	74.9 (5.6)	68.3 (18.8)
240	Imibenconazole	2.5	83.2 (26.0)	89.8 (6.7)	2.5	101.3 (3.6)	93.4 (4.0)	2.5	99.3 (3.0)	99.2 (0.1)	2.5	91.0 (5.6)	91.3 (7.5)	2.5	74.3 (4.9)	77.2 (6.2)
241	Imicyafos	5.0	97.3 (4.3)	95.6 (3.7)	2.5	106.3 (2.3)	86.8 (2.3)	2.5	112.5 (1.4)	90.4 (4.8)	2.5	113.8 (3.8)	105.4 (2.2)	2.5	93.8 (2.9)	100.3 (1.0)
242	Imidacloprid	2.5	96.4 (1.3)	104.6 (0.3)	2.5	107.1 (2.7)	90.9 (3.3)	2.5	108.7 (10.4)	89.0 (8.5)	2.5	100.0 (3.2)	100.9 (1.6)	2.5	93.9 (3.2)	88.6 (4.5)
243	Inabenfide	2.5	86.2 (8.6)	103.6 (4.1)	2.5	97.0 (1.7)	91.2 (8.2)	5.0	95.6 (1.7)	99.0 (3.6)	2.5	100.7 (1.5)	94.7 (3.2)	5.0	95.4 (3.6)	95.9 (2.2)
244	Indanofan	2.5	114.2 (3.0)	97.2 (1.6)	2.5	108.0 (2.2)	88.2 (2.7)	2.5	102.9 (1.8)	100.9 (1.6)	2.5	90.7 (5.4)	105.7 (1.1)	2.5	95.3 (2.6)	83.8 (1.0)
245	Indaziflam	20.0	N.D.	90.2 (2.2)	20.0	N.D.	84.0 (2.0)	20.0	N.D.	104.4 (0.4)	20.0	N.D.	103.4 (0.9)	25.0	N.D.	84.8 (3.9)
246	Indoxacarb	2.5	84.1 (1.7)	106.0 (1.1)	2.5	100.6 (3.1)	96.9 (4.4)	2.5	105.4 (1.7)	101.3 (0.2)	2.5	110.9 (5.2)	100.1 (2.7)	2.5	108.3 (0.9)	107.6 (2.0)
247	Ipconazole	2.5	88.1 (1.6)	104.4 (2.8)	2.5	102.9 (2.4)	95.5 (5.2)	2.5	105.3 (2.0)	99.4 (0.6)	2.5	101.1 (2.4)	94.3 (2.9)	2.5	92.0 (2.8)	91.5 (0.9)
248	Ipfencarbazone	2.5	80.4 (3.3)	90.9 (1.4)	5.0	99.5 (2.3)	89.4 (4.2)	2.5	79.3 (2.0)	102.6 (1.1)	2.5	83.1 (1.8)	89.3 (2.6)	2.5	100.6 (2.7)	89.6 (1.0)
249	Iprobenfos	5.0	79.7 (6.8)	96.0 (1.3)	5.0	105.3 (1.7)	89.0 (5.0)	2.5	88.6 (3.3)	90.7 (1.3)	2.5	97.8 (2.1)	108.0 (2.6)	5.0	107.1 (4.3)	93.8 (2.3)
250	Iprovalicarb	2.5	121.4 (3.5)	108.6 (2.8)	2.5	109.3 (2.4)	88.9 (28.1)	2.5	110.7 (2.0)	104.1 (0.4)	2.5	115.9 (3.0)	104.1 (1.6)	2.5	111.0 (2.8)	110.1 (0.9)
251	Isazofos	2.5	90.7 (5.2)	95.9 (3.3)	2.5	104.0 (1.4)	84.5 (3.1)	2.5	83.7 (1.5)	91.9 (0.7)	2.5	80.9 (1.8)	97.5 (2.9)	2.5	104.5 (1.7)	105.0 (2.3)
252	Isofenphos	2.5	105.0 (6.4)	103.6 (2.2)	2.5	107.9 (1.4)	97.2 (6.8)	2.5	86.0 (4.9)	99.7 (1.6)	2.5	107.6 (5.8)	97.0 (3.0)	2.5	84.6 (3.1)	82.8 (6.4)
253	Isofenphos-methyl	2.5	104.9 (6.9)	103.6 (2.2)	2.5	95.5 (6.0)	94.8 (7.4)	5.0	92.9 (6.7)	100.9 (0.5)	2.5	86.5 (6.9)	92.8 (3.5)	2.5	50.6 (12.4)	64.8 (6.5)
254	Isoprocarb	2.5	116.4 (5.4)	108.6 (1.3)	2.5	106.1 (2.7)	97.1 (3.7)	5.0	72.6 (3.9)	105.1 (0.5)	2.5	107.8 (8.5)	101.4 (1.6)	2.5	106.8 (2.4)	106.4 (2.3)
255	Isopropalin	2.5	110.5 (2.6)	104.0 (1.2)	2.5	106.3 (1.5)	92.1 (2.3)	2.5	95.8 (1.9)	100.3 (1.2)	2.5	106.3 (12.7)	92.3 (1.8)	2.5	72.2 (3.4)	74.2 (1.6)
256	Isoprothiolane	2.5	101.3 (9.7)	95.5 (1.4)	2.5	89.8 (4.4)	87.4 (5.3)	2.5	95.2 (2.1)	104.9 (2.7)	2.5	100.2 (4.1)	89.9 (2.1)	5.0	110.4 (2.1)	87.6 (2.0)
257	Isoproturon	2.5	82.3 (8.8)	101.9 (2.9)	2.5	107.9 (3.0)	86.5 (3.0)	2.5	102.1 (1.9)	95.9 (2.2)	2.5	109.5 (2.3)	104.5 (1.0)	2.5	103.1 (0.7)	90.4 (3.0)
258	Isopyrazam	20.0	N.D.	79.8 (0.6)	2.5	105.3 (3.8)	88.5 (5.8)	2.5	87.0 (7.9)	91.9 (1.1)	2.5	97.3 (1.8)	101.3 (2.8)	5.0	136.8 (1.0)	95.3 (2.0)
259	Isotianil	2.5	104.7 (2.8)	96.3 (0.9)	2.5	89.4 (7.6)	88.0 (5.4)	20.0	N.D.	101.6 (3.9)	2.5	99.3 (12.7)	84.7 (4.0)	2.5	96.7 (2.4)	95.6 (3.0)
260	Isouron	2.5	100.7 (5.3)	94.5 (1.3)	2.5	94.5 (3.5)	97.7 (7.6)	5.0	97.5 (0.8)	91.6 (1.4)	2.5	94.5 (4.9)	108.7 (1.7)	2.5	107.5 (0.8)	98.9 (5.1)
261	Isoxaben	2.5	79.8 (1.5)	104.1 (0.7)	2.5	103.9 (2.3)	86.1 (3.7)	2.5	95.8 (1.4)	99.1 (0.8)	2.5	89.3 (4.0)	106.0 (1.6)	5.0	100.4 (1.0)	93.4 (2.3)
262	Isoxadifen	2.5	43.5 (44.3)	28.1 (44.6)	2.5	23.2 (7.3)	32.7 (98.3)	10.0	32.8 (1.7)	26.0 (27.1)	2.5	N.D.	26.4 (45.9)	5.0	34.9 (14.6)	20.5 (49.6)
263	Isoxadifen-ethyl	5.0	91.7 (3.6)	90.6 (1.0)	2.5	86.1 (3.2)	85.3 (4.4)	2.5	73.3 (2.7)	88.7 (2.5)	2.5	77.2 (4.0)	85.8 (5.3)	2.5	105.8 (2.4)	89.1 (1.3)
264	Isoxathion	2.5	105.3 (10.7)	102.5 (3.5)	2.5	106.7 (3.1)	86.2 (3.9)	2.5	102.6 (1.6)	103.8 (0.9)	2.5	102.0 (1.4)	94.4 (2.7)	2.5	82.6 (2.7)	91.7 (4.4)
265	Ivermectin B1a	2.5	106.6 (2.4)	111.6 (1.3)	5.0	110.7 (4.7)	98.8 (4.2)	5.0	111.8 (4.7)	104.5 (2.3)	10.0	131.7 (9.1)	102.3 (1.9)	25.0	N.D.	96.0 (2.9)
266	Kresoxim-methyl	2.5	105.3 (3.1)	92.9 (1.8)	2.5	89.5 (5.4)	87.5 (5.9)	2.5	94.0 (1.8)	90.7 (1.7)	2.5	101.0 (0.2)	84.3 (4.4)	2.5	82.9 (5.0)	73.6 (2.8)
267	Lancotrione	2.5	39.7 (86.9)	29.9 (54.4)	2.5	3.3 (99.3)	38.5 (124.0)	10.0	N.D.	26.3 (26.9)	2.5	30.1 (78.2)	44.0 (40.8)	2.5	N.D.	26.5 (70.8)
268	Lenacil	2.5	116.2 (4.9)	106.1 (2.4)	2.5	108.8 (6.5)	88.8 (2.8)	5.0	85.3 (20.7)	100.6 (8.2)	2.5	101.1 (4.6)	100.3 (2.9)	2.5	101.2 (2.6)	104.7 (1.7)
269	Leptophos	2.5	86.0 (5.6)	99.0 (4.3)	5.0	100.1 (9.8)	95.6 (1.7)	20.0	N.D.	105.3 (8.5)	20.0	N.D.	79.1 (12.0)	10.0	43.1 (4.5)	57.3 (2.9)
270	Linuron	2.5	82.6 (9.3)	102.7 (1.3)	2.5	105.3 (1.4)	83.0 (16.0)	2.5	96.3 (2.8)	102.3 (0.6)	2.5	95.2 (2.8)	104.0 (1.8)	2.5	100.0 (1.3)	99.2 (4.1)
271	Lufenuron	2.5	82.6 (26.5)	91.7 (5.4)	2.5	108.4 (3.0)	91.7 (2.2)	2.5	105.9 (2.6)	106.6 (2.2)	2.5	96.4 (18.4)	96.1 (6.7)	2.5	72.4 (4.6)	88.8 (5.9)
272	Malaoxon	2.5	83.3 (4.2)	93.9 (1.6)	2.5	107.3 (1.7)	88.7 (2.8)	2.5	95.6 (2.7)	104.8 (1.3)	2.5	93.8 (5.2)	94.4 (1.2)	2.5	101.1 (0.8)	98.3 (3.4)
273	Malathion	2.5	105.8 (5.5)	91.2 (1.7)	2.5	105.6 (2.3)	85.0 (4.1)	2.5	108.4 (0.7)	93.9 (1.5)	2.5	107.0 (2.1)	95.9 (2.9)	2.5	105.8 (1.5)	91.2 (2.7)
274	Mandestrobin	2.5	88.4 (6.9)	104.2 (1.3)	2.5	108.2 (1.1)	98.6 (5.0)	2.5	107.3 (1.5)	101.7 (2.7)	2.5	96.7 (4.4)	103.1 (4.2)	2.5	102.3 (1.6)	103.4 (1.3)
275	Mandipropamid	2.5	86.7 (3.8)	88.1 (3.7)	2.5	105.0 (0.9)	90.0 (1.9)	2.5	92.0 (1.8)	105.0 (1.8)	2.5	107.0 (2.1)	96.5 (2.2)	5.0	109.2 (0.3)	100.5 (1.0)
276	Mecarbam	2.5	86.9 (4.6)	106.4 (0.8)	2.5	106.4 (2.3)	97.6 (4.1)	2.5	85.9 (4.0)	86.9 (0.4)	2.5	89.2 (3.6)	104.9 (0.9)	2.5	103.4 (1.9)	103.7 (3.3)
277	Mefenacet	2.5	98.8 (5.9)	105.7 (1.4)	2.5	105.5 (1.1)	84.8 (5.6)	2.5	109.8 (1.2)	101.1 (1.3)	2.5	88.3 (7.1)	105.8 (1.7)	2.5	100.6 (1.2)	102.2 (0.6)
278	Mefenpyr-diethyl	2.5	92.5 (4.2)	107.0 (2.7)	5.0	83.3 (2.1)	94.7 (6.5)	2.5	91.9 (2.5)	101.1 (0.8)	2.5	94.2 (2.4)	99.5 (2.5)	2.5	103.1 (3.6)	97.7 (3.0)
279	Mefentrifluconazole	2.5	106.8 (2.6)	102.2 (1.1)	2.5	105.0 (2.8)	97.3 (4.6)	2.5	105.2 (3.3)	103.7 (0.4)	2.5	102.2 (2.4)	100.8 (2.8)	2.5	105.7 (0.4)	105.8 (1.6)
280	Mepanipyrim	2.5	102.3 (5.2)	93.2 (2.8)	2.5	103.2 (2.1)	85.1 (4.7)	2.5	73.5 (2.2)	88.6 (1.1)	2.5	106.7 (3.7)	90.8 (0.9)	2.5	84.1 (2.0)	75.5 (1.3)
281	Mephosfolan	2.5	90.3 (4.6)	102.5 (2.5)	2.5	105.4 (2.5)	86.9 (4.7)	2.5	96.0 (2.6)	104.7 (1.4)	2.5	92.4 (2.7)	107.3 (0.6)	2.5	107.5 (0.6)	106.7 (3.2)
282	Mepronil	5.0	104.3 (5.3)	95.0 (1.2)	5.0	98.5 (0.3)	86.7 (2.6)	2.5	83.3 (2.1)	89.8 (0.7)	2.5	113.3 (4.0)	92.0 (3.6)	2.5	101.6 (1.5)	85.7 (3.2)
283	Mesosulfuron-methyl	2.5	75.1 (26.4)	70.2 (18.7)	2.5	55.2 (10.4)	68.1 (29.8)	5.0	59.1 (4.6)	63.8 (7.8)	2.5	82.3 (18.8)	76.1 (7.9)	2.5	62.5 (7.5)	67.3 (18.1)
284	Mesotrione	2.5	N.D.	31.4 (38.2)	2.5	7.8 (21.5)	40.4 (95.3)	5.0	20.9 (14.9)	33.2 (17.4)	2.5	32.9 (28.4)	45.2 (33.2)	5.0	23.7 (2.0)	26.5 (46.7)
285	Metaflumizone	2.5	95.6 (0.5)	98.4 (1.5)	2.5	96.5 (2.1)	94.2 (4.1)	2.5	93.5 (1.1)	97.4 (0.6)	2.5	93.5 (3.4)	95.9 (3.8)	2.5	97.6 (1.3)	100.6 (1.2)
286	Metamifop	2.5	74.2 (3.7)	104.9 (1.3)	2.5	103.5 (2.4)	98.1 (4.5)	2.5	94.5 (1.6)	96.1 (0.6)	2.5	104.7 (2.3)	102.9 (2.1)	2.5	94.1 (1.3)	101.4 (2.5)
287	Metamitron	5.0	99.7 (2.8)	91.3 (2.2)	2.5	107.6 (2.4)	88.3 (11.0)	2.5	86.2 (2.9)	86.0 (4.4)	2.5	100.4 (3.9)	91.3 (2.8)	2.5	80.0 (0.7)	77.1 (5.7)
288	Metazosulfuron	2.5	97.1 (16.0)	89.4 (11.3)	2.5	107.1 (5.2)	81.5 (9.7)	2.5	88.6 (4.6)	83.9 (4.0)	2.5	112.2 (9.4)	81.1 (4.6)	2.5	114.5 (4.5)	87.4 (8.5)
289	Metconazole	2.5	87.9 (3.3)	106.4 (1.4)	2.5	105.9 (3.0)	95.9 (3.1)	2.5	108.7 (0.6)	101.1 (0.3)	2.5	103.3 (3.0)	104.3 (2.7)	2.5	101.1 (2.5)	104.6 (0.9)
290	Methabenzthiazuron	5.0	101.8 (4.9)	92.8 (1.6)	2.5	107.6 (1.8)	86.0 (3.3)	2.5	96.0 (2.3)	88.1 (0.7)	2.5	102.0 (4.4)	90.7 (0.9)	2.5	101.5 (2.0)	102.0 (2.1)
291	Methacrifos	2.5	95.9 (3.6)	106.5 (2.7)	5.0	116.4 (4.6)	77.9 (41.7)	10.0	107.6 (7.1)	101.9 (3.9)	2.5	118.1 (4.6)	105.6 (1.7)	5.0	116.7 (1.3)	95.2 (2.4)
292	Methidathion	5.0	91.1 (5.2)	95.5 (1.7)	2.5	107.4 (3.3)	72.5 (37.3)	2.5	111.2 (4.3)	104.9 (1.6)	2.5	89.1 (3.8)	92.0 (1.3)	5.0	109.1 (2.8)	87.6 (2.6)
293	Methiocarb	2.5	107.3 (3.0)	103.5 (3.1)	2.5	101.0 (3.0)	86.9 (21.1)	2.5	104.7 (16.6)	98.2 (4.4)	2.5	108.0 (5.3)	106.3 (2.1)	2.5	96.3 (2.6)	96.2 (3.6)
294	Methoprotryn	2.5	94.7 (3.3)	94.7 (2.9)	2.5	91.0 (2.8)	86.9 (28.5)	5.0	101.2 (1.5)	94.5 (1.4)	2.5	99.7 (3.3)	102.0 (0.6)	2.5	110.9 (2.0)	89.2 (3.1)
295	Methoxyfenozide	2.5	89.3 (5.5)	91.6 (0.6)	2.5	103.8 (3.3)	85.8 (4.2)	2.5	104.8 (1.2)	93.2 (1.2)	2.5	95.3 (4.1)	96.0 (2.6)	2.5	106.8 (1.3)	94.6 (2.9)
296	Metolachlor	2.5	97.2 (5.0)	104.8 (2.2)	2.5	89.8 (2.4)	98.0 (3.8)	2.5	96.5 (0.8)	100.4 (2.5)	2.5	94.8 (5.1)	99.6 (2.2)	2.5	108.1 (1.7)	93.2 (2.9)
297	Metolcarb	2.5	109.6 (8.0)	104.4 (0.4)	2.5	102.5 (1.2)	99.4 (3.4)	10.0	81.6 (8.2)	105.5 (2.1)	2.5	100.4 (1.8)	102.8 (3.1)	2.5	81.3 (1.0)	105.8 (3.2)
298	Metominostrobin	2.5	75.6 (6.7)	92.0 (1.2)	2.5	108.5 (3.0)	86.9 (4.9)	2.5	90.2 (1.6)	93.2 (1.0)	2.5	85.7 (5.1)	95.4 (2.2)	2.5	104.3 (0.8)	92.9 (1.7)
299	Metosulam	2.5	51.9 (39.6)	55.0 (26.9)	2.5	29.1 (5.5)	58.2 (55.9)	2.5	36.4 (4.2)	53.5 (12.5)	2.5	51.2 (30.5)	59.7 (15.1)	2.5	31.9 (13.1)	65.1 (31.4)
300	Metrafenone	2.5	105.1 (0.4)	103.8 (1.6)	2.5	100.1 (4.3)	97.8 (5.0)	2.5	106.0 (0.7)	101.6 (1.6)	2.5	97.3 (3.0)	99.7 (3.4)	2.5	85.6 (1.9)	90.2 (1.6)
301	Metribuzin	2.5	111.4 (6.0)	93.5 (2.2)	2.5	92.0 (4.2)	89.0 (5.6)	5.0	77.5 (3.3)	90.7 (1.5)	2.5	91.5 (6.1)	93.2 (0.8)	2.5	81.8 (2.0)	73.7 (4.1)
302	Mevinphos	2.5	100.1 (8.6)	97.6 (4.2)	2.5	103.7 (1.0)	98.3 (6.0)	2.5	91.6 (1.9)	102.7 (2.4)	2.5	76.8 (4.3)	102.0 (2.0)	2.5	48.7 (4.6)	101.2 (3.1)
303	MGK-264	2.5	115.9 (6.1)	94.9 (1.7)	2.5	80.7 (7.4)	99.0 (3.9)	2.5	100.8 (1.3)	104.0 (0.9)	2.5	102.0 (2.1)	96.7 (3.0)	2.5	97.8 (3.0)	88.3 (2.9)
304	Milbemectin A4	5.0	83.8 (18.4)	101.1 (5.8)	5.0	88.5 (17.9)	96.5 (7.4)	20.0	N.D.	93.4 (5.7)	5.0	95.8 (5.9)	96.2 (3.4)	25.0	N.D.	87.1 (5.2)
305	Molinate	2.5	101.5 (5.2)	109.5 (1.4)	2.5	98.3 (1.4)	97.3 (2.7)	2.5	102.4 (0.6)	99.9 (0.7)	2.5	98.9 (0.6)	96.7 (2.0)	2.5	80.9 (2.0)	84.8 (4.9)
306	Monocrotophos	2.5	106.9 (8.3)	89.0 (30.3)	2.5	110.7 (1.7)	95.9 (7.3)	2.5	102.6 (22.9)	92.8 (3.8)	2.5	101.7 (9.9)	104.9 (8.3)	2.5	84.7 (1.5)	74.3 (31.0)
307	Monolinuron	5.0	85.8 (4.3)	97.5 (1.8)	5.0	94.0 (1.8)	89.4 (5.1)	2.5	98.9 (2.5)	93.1 (1.3)	2.5	106.1 (6.2)	92.4 (1.3)	5.0	121.7 (1.7)	95.9 (2.3)
308	Myclobutanil	5.0	97.8 (1.7)	100.1 (2.3)	5.0	106.3 (3.5)	96.7 (5.0)	2.5	101.5 (3.5)	95.4 (0.0)	2.5	89.9 (5.2)	86.5 (1.1)	5.0	116.8 (2.4)	95.0 (1.0)
309	Naftalofos	2.5	100.4 (3.5)	105.8 (0.7)	2.5	86.3 (1.9)	98.5 (5.1)	2.5	101.3 (1.8)	94.3 (1.5)	2.5	96.9 (2.1)	104.0 (1.6)	25.0	N.D.	101.7 (3.6)
310	Napropamide	5.0	104.6 (4.2)	93.4 (3.1)	2.5	105.1 (2.5)	86.2 (3.3)	2.5	89.9 (0.9)	102.5 (2.2)	2.5	105.3 (2.1)	95.7 (2.3)	2.5	106.4 (2.3)	105.0 (1.2)
311	Neburon	5.0	106.6 (2.4)	92.3 (2.7)	5.0	96.0 (3.4)	86.6 (4.5)	5.0	104.0 (3.2)	90.8 (1.2)	5.0	101.4 (2.2)	92.6 (2.7)	5.0	81.7 (2.7)	87.4 (3.0)
312	Nicosulfuron	2.5	44.5 (28.7)	40.6 (21.7)	2.5	30.1 (5.8)	44.3 (47.7)	5.0	39.1 (5.4)	38.4 (12.2)	2.5	48.2 (16.9)	46.1 (16.9)	5.0	23.2 (14.7)	36.5 (34.1)
313	Nitenpyram	2.5	88.2 (5.1)	89.7 (2.1)	2.5	101.9 (3.8)	98.2 (2.0)	2.5	102.9 (2.2)	94.0 (4.0)	2.5	102.2 (2.9)	87.2 (2.7)	2.5	56.0 (1.9)	45.7 (40.1)
314	Norea (noruron)	2.5	104.5 (4.2)	91.2 (3.9)	2.5	101.8 (3.0)	87.1 (15.9)	2.5	88.7 (1.1)	101.4 (1.7)	2.5	109.7 (5.7)	100.6 (2.2)	2.5	96.4 (2.4)	98.5 (3.3)
315	Norflurazon	2.5	87.6 (4.4)	90.5 (1.6)	2.5	106.6 (1.6)	99.9 (4.6)	2.5	99.6 (2.1)	95.0 (1.6)	2.5	108.1 (4.0)	93.8 (2.6)	2.5	100.3 (0.4)	92.0 (1.6)
316	Novaluron	2.5	103.9 (1.5)	102.7 (3.7)	2.5	100.5 (2.7)	93.0 (13.0)	2.5	95.3 (2.4)	100.1 (1.4)	2.5	95.8 (4.6)	100.2 (3.2)	2.5	96.3 (1.3)	104.0 (3.0)
317	Nuarimol	2.5	108.2 (5.8)	106.7 (3.1)	2.5	90.9 (2.1)	98.9 (3.0)	2.5	99.3 (0.3)	99.7 (2.4)	2.5	113.2 (0.1)	97.0 (2.8)	5.0	114.5 (2.3)	98.0 (3.6)
318	Ofurace	5.0	105.5 (4.3)	93.3 (1.6)	5.0	101.2 (2.0)	89.0 (4.3)	2.5	97.8 (1.3)	93.6 (1.1)	5.0	105.8 (3.4)	94.2 (0.9)	2.5	102.7 (0.9)	90.7 (3.0)
319	Omethoate	2.5	92.8 (18.0)	73.9 (1.0)	5.0	102.8 (10.0)	94.6 (11.3)	2.5	107.8 (16.2)	75.2 (3.9)	2.5	88.0 (22.9)	87.1 (18.6)	5.0	60.1 (2.0)	77.4 (10.0)
320	Orthosulfamuron	2.5	80.5 (15.6)	77.4 (12.6)	2.5	65.3 (5.9)	77.7 (17.8)	2.5	64.6 (1.8)	75.9 (8.1)	2.5	82.2 (9.0)	80.5 (3.5)	2.5	61.8 (5.1)	77.9 (11.8)
321	Orysastrobin	2.5	105.2 (6.7)	103.6 (1.4)	2.5	99.2 (2.6)	95.6 (5.3)	2.5	105.4 (0.4)	105.4 (2.1)	2.5	86.4 (4.2)	89.3 (2.9)	2.5	100.3 (1.3)	97.8 (2.6)
322	Orysastrobin metabolite (F001)	2.5	93.7 (3.9)	105.0 (2.3)	2.5	103.2 (2.2)	98.1 (3.1)	2.5	90.4 (2.8)	92.0 (0.8)	2.5	106.6 (2.9)	106.5 (2.3)	2.5	107.2 (1.7)	111.4 (0.5)
323	Oryzalin	2.5	112.0 (4.2)	103.7 (4.8)	2.5	109.2 (2.9)	105.4 (3.2)	2.5	106.0 (1.2)	102.8 (1.2)	2.5	115.1 (2.3)	108.4 (2.7)	2.5	116.5 (1.5)	114.3 (2.8)
324	Oxadiargyl	2.5	88.8 (2.6)	95.9 (1.1)	2.5	105.6 (2.5)	100.9 (4.0)	2.5	89.8 (0.6)	91.4 (0.3)	2.5	92.3 (1.3)	85.4 (3.6)	2.5	101.2 (2.3)	95.4 (1.4)
325	Oxadiazon	2.5	104.4 (3.2)	95.9 (1.4)	2.5	97.2 (3.6)	99.8 (2.5)	2.5	86.0 (1.9)	106.9 (0.5)	2.5	92.7 (2.9)	97.5 (3.6)	2.5	91.6 (3.6)	87.0 (2.8)
326	Oxadixyl	2.5	91.4 (1.8)	107.3 (0.6)	5.0	106.6 (0.7)	91.1 (4.5)	2.5	96.4 (1.7)	105.6 (2.0)	2.5	85.1 (2.2)	105.4 (2.6)	2.5	104.5 (0.5)	97.3 (2.2)
327	Oxathiapiprolin	2.5	87.2 (5.2)	107.2 (4.2)	2.5	95.9 (1.1)	100.9 (4.7)	2.5	105.6 (2.7)	99.7 (1.6)	2.5	104.9 (1.7)	106.7 (3.4)	2.5	107.7 (1.9)	106.1 (4.2)
328	Oxaziclomefone	2.5	82.7 (3.4)	105.7 (0.8)	2.5	104.7 (2.7)	97.7 (4.8)	2.5	102.1 (1.5)	100.4 (0.5)	2.5	102.8 (2.1)	99.2 (2.2)	2.5	77.4 (1.3)	87.3 (3.5)
329	Oxycarboxin	2.5	106.5 (3.3)	103.1 (2.0)	2.5	103.4 (1.4)	102.7 (2.6)	2.5	104.7 (0.4)	93.4 (2.0)	2.5	103.6 (2.0)	94.4 (2.3)	5.0	103.4 (2.6)	106.5 (1.5)
330	Oxydemeton-methyl	2.5	97.5 (11.4)	81.7 (1.0)	2.5	100.9 (12.4)	98.8 (13.4)	2.5	85.7 (3.2)	98.5 (3.1)	2.5	108.5 (10.7)	100.8 (12.3)	5.0	73.3 (1.5)	78.7 (4.1)
331	Oxyfluorfen	5.0	110.4 (3.8)	96.5 (1.7)	5.0	121.7 (8.9)	89.2 (4.4)	10.0	77.2 (7.2)	96.6 (10.7)	2.5	113.8 (6.5)	97.1 (1.2)	5.0	86.4 (6.4)	93.2 (4.7)
332	Paclobutrazol	2.5	110.5 (1.6)	95.7 (0.9)	2.5	94.7 (4.9)	88.7 (4.0)	2.5	91.6 (3.8)	104.7 (2.7)	2.5	95.3 (2.9)	91.6 (2.2)	2.5	107.7 (1.1)	89.9 (2.6)
333	Parathion	2.5	105.4 (5.4)	90.1 (2.7)	2.5	101.7 (5.1)	93.1 (7.7)	10.0	87.4 (9.6)	76.0 (7.0)	20.0	N.D.	90.1 (7.7)	5.0	66.1 (11.4)	72.3 (3.8)
334	Parathion-methyl	2.5	87.6 (3.8)	98.0 (1.7)	2.5	91.7 (6.5)	87.9 (3.9)	5.0	79.6 (4.9)	98.8 (1.9)	2.5	105.6 (2.3)	108.4 (2.7)	2.5	59.7 (7.6)	77.6 (3.8)
335	Pebulate	2.5	98.8 (2.8)	103.1 (1.2)	2.5	96.0 (1.7)	95.6 (3.9)	2.5	99.2 (1.0)	100.7 (0.6)	2.5	96.1 (1.9)	95.1 (2.2)	2.5	71.1 (1.1)	77.0 (2.0)
336	Penconazole	2.5	99.4 (0.5)	95.6 (1.7)	2.5	105.4 (3.7)	87.9 (5.1)	2.5	88.0 (5.1)	89.3 (2.1)	2.5	81.5 (5.3)	94.1 (3.2)	2.5	84.9 (4.9)	78.3 (1.4)
337	Pencycuron	2.5	90.5 (2.8)	106.4 (0.9)	2.5	101.8 (5.0)	96.6 (5.0)	2.5	94.8 (0.7)	102.6 (2.3)	2.5	102.3 (0.4)	99.7 (2.7)	2.5	83.8 (1.1)	92.2 (0.6)
338	Pendimethalin	2.5	113.0 (3.0)	106.9 (1.0)	2.5	109.7 (3.5)	94.8 (1.6)	5.0	89.2 (0.3)	95.7 (1.4)	2.5	106.2 (4.0)	91.3 (2.7)	2.5	74.2 (2.6)	74.9 (1.3)
339	Penflufen	2.5	98.9 (4.1)	92.3 (1.0)	5.0	87.5 (3.3)	88.8 (4.7)	2.5	84.3 (1.9)	90.1 (3.1)	2.5	101.3 (3.8)	92.0 (5.6)	2.5	113.7 (1.8)	87.7 (2.5)
340	Penoxsulam	2.5	78.6 (25.9)	75.5 (16.7)	2.5	56.0 (6.9)	76.2 (38.4)	5.0	60.9 (2.9)	73.8 (6.7)	2.5	76.1 (19.0)	82.3 (9.2)	2.5	72.9 (8.6)	106.1 (14.1)
341	Penthiopyrad	5.0	102.0 (1.7)	93.0 (2.0)	5.0	80.6 (6.0)	86.8 (5.2)	2.5	88.0 (8.3)	89.4 (1.4)	2.5	90.6 (5.2)	88.6 (2.8)	2.5	102.0 (4.5)	79.2 (2.5)
342	Pentoxazone	2.5	125.4 (1.6)	112.1 (2.5)	2.5	123.3 (9.7)	100.2 (1.5)	20.0	N.D.	99.6 (2.7)	2.5	107.6 (2.7)	97.7 (5.5)	5.0	78.9 (5.7)	89.7 (4.6)
343	Permethrin	2.5	101.3 (2.9)	103.9 (1.8)	5.0	104.2 (6.8)	98.7 (4.1)	2.5	99.9 (3.4)	95.6 (0.5)	5.0	94.5 (19.7)	92.7 (4.9)	2.5	52.4 (0.6)	58.6 (2.7)
344	Phenothrin	2.5	81.9 (4.5)	102.7 (1.3)	2.5	98.5 (3.5)	96.0 (1.7)	2.5	95.4 (2.5)	100.8 (0.3)	2.5	96.1 (16.1)	96.0 (4.4)	2.5	45.8 (3.8)	59.4 (3.0)
345	Phenthoate	5.0	89.7 (5.9)	92.8 (1.4)	5.0	76.8 (2.7)	88.8 (4.8)	2.5	89.1 (1.2)	89.5 (0.9)	2.5	89.7 (2.6)	89.3 (3.0)	2.5	100.4 (3.0)	79.8 (1.9)
346	Phorate oxon	5.0	102.3 (2.3)	92.4 (1.5)	5.0	101.3 (3.0)	69.9 (41.5)	2.5	93.4 (3.9)	93.2 (1.5)	2.5	108.9 (3.5)	95.3 (1.0)	5.0	92.0 (2.8)	93.0 (3.2)
347	Phorate oxon sulfone	5.0	99.9 (3.4)	94.9 (2.3)	2.5	105.5 (2.5)	99.9 (6.4)	2.5	102.5 (0.8)	96.6 (1.4)	20.0	N.D.	100.6 (0.5)	2.5	90.2 (1.2)	97.0 (3.0)
348	Phorate oxon sulfoxide	5.0	71.2 (5.8)	90.7 (6.1)	2.5	103.4 (2.1)	96.8 (7.9)	2.5	91.5 (1.5)	91.0 (4.8)	5.0	97.5 (17.2)	102.9 (5.0)	2.5	115.8 (1.4)	116.8 (18.4)
349	Phorate sulfone	5.0	104.5 (1.6)	94.7 (1.0)	2.5	101.4 (2.1)	89.9 (2.7)	2.5	103.4 (3.2)	95.8 (3.1)	2.5	108.8 (3.9)	96.1 (3.3)	2.5	104.5 (0.6)	98.0 (0.5)
350	Phorate sulfoxide	5.0	87.0 (7.6)	95.0 (1.8)	2.5	103.8 (2.5)	88.3 (3.7)	2.5	100.9 (1.5)	83.5 (0.5)	2.5	92.2 (5.3)	99.4 (1.6)	2.5	105.0 (1.9)	97.0 (2.4)
351	Phosalone	2.5	85.4 (3.4)	95.2 (0.9)	5.0	90.8 (3.6)	92.5 (5.9)	5.0	101.8 (1.5)	92.8 (1.5)	2.5	99.1 (4.9)	90.4 (3.7)	2.5	103.8 (3.6)	91.1 (5.8)
352	Phosfolan	2.5	99.0 (13.3)	86.3 (4.5)	2.5	106.6 (1.9)	95.7 (4.7)	2.5	98.1 (2.2)	106.4 (1.9)	2.5	90.2 (2.7)	104.1 (1.7)	2.5	95.2 (0.3)	92.3 (2.5)
353	Phosmet	20.0	N.D.	93.4 (1.5)	20.0	N.D.	93.0 (2.5)	20.0	N.D.	103.0 (2.1)	20.0	N.D.	85.9 (1.8)	25.0	N.D.	97.4 (1.6)
354	Phosphamidon	2.5	101.1 (3.4)	98.4 (0.5)	5.0	97.0 (3.1)	91.8 (4.7)	5.0	109.4 (1.6)	92.6 (1.6)	2.5	104.7 (1.5)	105.9 (3.6)	5.0	113.8 (0.7)	98.7 (2.4)
355	Phoxim	2.5	103.8 (10.0)	102.4 (2.8)	2.5	104.2 (1.9)	99.2 (6.3)	2.5	106.6 (1.0)	103.6 (1.1)	2.5	86.2 (4.8)	103.6 (1.5)	2.5	86.0 (3.3)	96.3 (2.8)
356	Picarbutrazox	2.5	108.4 (2.1)	102.8 (3.7)	2.5	98.1 (2.7)	96.5 (6.4)	2.5	103.0 (1.5)	104.1 (0.4)	2.5	103.3 (2.1)	101.9 (0.1)	2.5	99.0 (1.3)	106.0 (1.6)
357	Picolinafen	2.5	92.2 (5.4)	84.6 (2.4)	2.5	104.9 (3.7)	86.9 (5.3)	2.5	106.3 (0.7)	93.5 (1.8)	2.5	101.6 (2.0)	100.4 (1.7)	2.5	68.7 (1.8)	81.4 (6.1)
358	Picoxystrobin	5.0	104.9 (3.0)	98.0 (2.0)	2.5	99.2 (1.6)	98.7 (3.3)	2.5	87.5 (3.4)	101.3 (1.6)	2.5	95.7 (2.6)	101.2 (2.0)	2.5	111.1 (1.9)	92.7 (1.8)
359	Pinoxaden	20.0	N.D.	90.0 (1.4)	2.5	97.7 (1.5)	87.6 (7.0)	2.5	94.2 (1.2)	99.0 (1.6)	2.5	15.8 (10.2)	21.0 (9.4)	2.5	19.5 (3.1)	24.7 (2.3)
360	Pinoxaden metabolite (SYN 505164)	2.5	114.8 (1.5)	103.6 (1.0)	2.5	109.5 (2.0)	98.1 (3.0)	5.0	110.3 (1.4)	98.8 (0.8)	2.5	111.4 (2.6)	106.0 (1.3)	2.5	110.1 (1.4)	108.2 (1.9)
361	Piperonyl butoxide	2.5	116.3 (3.7)	97.8 (1.9)	2.5	114.0 (3.8)	95.8 (2.7)	2.5	118.9 (0.7)	100.5 (0.7)	2.5	112.6 (3.2)	96.2 (2.7)	2.5	85.2 (4.0)	79.6 (3.3)
362	Piperophos	2.5	84.1 (4.6)	104.6 (2.3)	2.5	102.0 (4.2)	94.6 (6.0)	2.5	106.9 (3.1)	101.7 (0.9)	2.5	91.8 (4.0)	102.1 (2.7)	2.5	89.4 (1.5)	97.1 (2.9)
363	Pirimicarb	2.5	105.6 (3.6)	107.2 (2.0)	2.5	94.3 (1.8)	99.2 (7.0)	2.5	79.1 (2.2)	103.2 (0.5)	2.5	84.6 (4.1)	103.1 (1.7)	2.5	106.6 (0.3)	90.2 (3.0)
364	Pirimiphos-ethyl	2.5	99.6 (20.5)	91.0 (9.6)	2.5	92.5 (6.4)	92.8 (5.9)	2.5	89.5 (1.3)	100.3 (1.1)	2.5	89.7 (10.7)	87.2 (8.9)	2.5	65.2 (4.2)	57.9 (9.8)
365	Pirimiphos-methyl	2.5	102.4 (6.4)	103.2 (1.1)	2.5	78.0 (3.6)	97.9 (7.1)	2.5	93.4 (1.4)	101.8 (2.8)	2.5	94.8 (1.8)	96.6 (2.2)	2.5	84.4 (1.5)	79.0 (4.9)
366	Pretilachlor	2.5	106.9 (2.5)	108.3 (1.4)	2.5	102.9 (3.8)	93.8 (10.9)	2.5	105.7 (2.3)	101.2 (0.4)	2.5	100.3 (1.9)	98.1 (2.7)	2.5	80.3 (3.3)	84.3 (3.5)
367	Probenazole	2.5	49.3 (35.2)	57.4 (17.3)	2.5	42.2 (4.7)	52.3 (69.0)	5.0	42.1 (3.3)	73.5 (4.6)	2.5	55.7 (15.5)	16.5 (68.8)	2.5	24.1 (22.0)	47.1 (12.7)
368	Prochloraz metabolite (BTS 44595)	2.5	112.8 (2.3)	95.5 (1.5)	2.5	107.0 (1.8)	101.5 (3.4)	2.5	81.6 (8.1)	102.1 (1.7)	2.5	103.5 (4.2)	90.8 (2.0)	2.5	107.3 (1.4)	98.6 (2.3)
369	Procymidone	5.0	113.4 (2.4)	93.0 (1.6)	5.0	105.7 (9.1)	92.9 (4.5)	20.0	N.D.	100.1 (7.2)	2.5	106.6 (8.2)	93.9 (1.6)	5.0	101.0 (1.2)	86.3 (0.5)
370	Prodiamine	5.0	95.7 (14.7)	93.8 (2.7)	2.5	86.1 (7.5)	96.5 (2.6)	2.5	86.1 (4.2)	98.8 (0.5)	2.5	103.2 (6.0)	96.2 (3.8)	5.0	118.4 (5.1)	76.9 (6.1)
371	Profenofos	2.5	103.5 (3.5)	109.2 (1.1)	2.5	81.9 (4.3)	87.0 (4.0)	2.5	103.2 (1.0)	94.1 (0.9)	2.5	79.6 (3.0)	98.5 (1.9)	2.5	92.4 (0.3)	89.2 (2.8)
372	Prohydrojasmon	5.0	116.9 (1.9)	96.9 (1.8)	2.5	96.2 (3.3)	89.5 (4.1)	2.5	82.5 (3.6)	89.5 (0.3)	2.5	90.9 (5.0)	88.4 (3.2)	2.5	110.7 (1.9)	82.8 (1.3)
373	Promecarb	2.5	82.7 (7.4)	104.0 (0.9)	2.5	107.9 (1.4)	98.5 (5.7)	2.5	97.6 (5.5)	98.3 (2.4)	2.5	88.4 (5.1)	106.2 (0.8)	2.5	103.2 (2.4)	103.5 (4.5)
374	Prometryn	2.5	110.2 (8.0)	106.0 (1.4)	2.5	106.3 (3.8)	96.3 (3.7)	2.5	111.7 (0.9)	104.9 (1.7)	2.5	106.6 (2.7)	99.7 (2.7)	2.5	93.2 (1.5)	93.9 (3.3)
375	Propachlor	2.5	104.5 (7.9)	95.0 (2.4)	2.5	86.8 (4.2)	87.2 (3.7)	2.5	97.2 (1.5)	92.0 (2.4)	2.5	96.5 (2.9)	104.0 (1.0)	2.5	113.1 (1.1)	90.2 (2.8)
376	Propamocarb	2.5	95.3 (15.7)	72.8 (1.3)	2.5	100.0 (20.3)	107.7 (17.4)	5.0	104.9 (16.2)	74.3 (1.8)	2.5	100.9 (20.6)	98.4 (16.9)	2.5	60.6 (7.7)	82.8 (12.7)
377	Propanil	2.5	98.5 (4.0)	94.1 (1.6)	5.0	105.6 (2.5)	84.7 (30.2)	2.5	101.7 (2.6)	92.8 (4.0)	2.5	99.9 (6.4)	92.8 (1.8)	2.5	110.7 (1.3)	97.8 (2.7)
378	Propargite	2.5	101.9 (2.1)	102.5 (0.4)	2.5	97.9 (4.2)	97.3 (3.8)	2.5	101.1 (2.1)	100.0 (2.2)	2.5	95.5 (1.9)	96.0 (4.9)	5.0	66.7 (2.3)	73.3 (2.4)
379	Propazine	2.5	101.6 (9.7)	93.6 (3.4)	2.5	107.9 (1.6)	83.3 (30.8)	2.5	94.8 (1.2)	102.1 (1.8)	2.5	92.7 (7.1)	104.3 (1.7)	2.5	89.4 (1.5)	94.5 (5.4)
380	Propetamphos	2.5	103.9 (4.0)	96.4 (1.1)	2.5	82.7 (1.8)	86.1 (3.4)	2.5	83.6 (15.2)	103.1 (2.0)	2.5	98.5 (3.4)	94.9 (1.1)	2.5	116.4 (1.7)	89.2 (1.7)
381	Propiconazole	2.5	96.0 (3.2)	98.5 (2.2)	5.0	106.9 (1.7)	91.9 (3.9)	2.5	104.8 (1.4)	100.7 (0.2)	2.5	99.0 (1.9)	101.4 (3.6)	2.5	112.9 (0.2)	100.6 (1.2)
382	Propisochlor	2.5	89.8 (4.1)	94.9 (0.8)	2.5	81.8 (2.8)	88.8 (4.9)	5.0	83.1 (4.2)	91.5 (0.6)	2.5	82.5 (4.5)	102.9 (3.5)	2.5	93.8 (1.6)	92.1 (1.9)
383	Propoxur	2.5	110.1 (3.8)	93.7 (2.0)	2.5	105.1 (1.2)	88.1 (5.9)	10.0	94.1 (4.1)	104.6 (1.6)	2.5	113.6 (2.9)	96.0 (1.3)	2.5	112.7 (2.3)	94.3 (3.2)
384	Propyrisulfuron	2.5	84.9 (37.9)	75.4 (23.4)	2.5	49.9 (7.7)	66.6 (29.5)	2.5	54.3 (2.7)	65.9 (7.2)	2.5	69.3 (17.6)	70.9 (10.5)	2.5	48.9 (7.5)	70.7 (19.7)
385	Proquinazid	2.5	98.3 (5.0)	101.0 (0.2)	2.5	94.2 (3.3)	90.4 (0.6)	2.5	99.4 (1.3)	98.1 (2.3)	2.5	86.4 (2.4)	84.7 (3.2)	2.5	46.1 (1.9)	53.4 (1.6)
386	Prosulfocarb	2.5	106.5 (3.7)	103.0 (0.3)	2.5	103.2 (4.0)	97.6 (3.8)	2.5	94.7 (1.6)	102.5 (1.0)	2.5	99.6 (1.6)	97.0 (2.3)	2.5	72.2 (2.0)	81.4 (2.9)
387	Prothiofos	2.5	102.5 (1.2)	102.1 (2.5)	2.5	99.6 (1.1)	96.7 (0.9)	5.0	99.5 (5.5)	101.6 (2.8)	5.0	89.1 (9.2)	81.9 (4.0)	2.5	53.6 (3.4)	60.8 (3.0)
388	Pydiflumetofen	2.5	95.5 (3.0)	107.5 (1.2)	2.5	106.8 (2.5)	100.3 (4.4)	2.5	107.6 (1.4)	91.8 (0.5)	2.5	107.5 (2.8)	102.1 (5.1)	2.5	95.2 (2.0)	89.5 (0.9)
389	Pyflubumide	2.5	103.7 (1.6)	104.3 (0.8)	2.5	95.1 (3.2)	97.0 (4.0)	2.5	100.1 (0.6)	99.9 (1.7)	2.5	104.7 (3.1)	104.8 (2.5)	2.5	85.2 (2.3)	97.6 (2.1)
390	Pyflubumide-NH	2.5	103.8 (0.3)	103.0 (1.5)	2.5	95.6 (3.2)	96.8 (3.8)	2.5	98.9 (2.1)	100.8 (1.2)	2.5	99.6 (3.4)	103.0 (3.0)	2.5	97.6 (1.3)	107.7 (3.0)
391	Pyracarbolid	2.5	108.8 (3.5)	92.9 (1.9)	2.5	90.7 (4.5)	84.4 (6.0)	2.5	91.2 (0.2)	86.7 (0.8)	2.5	115.4 (4.0)	93.0 (0.8)	2.5	81.1 (2.0)	76.8 (5.4)
392	Pyraclofos	2.5	86.3 (1.9)	97.0 (0.4)	2.5	108.6 (2.9)	90.4 (2.5)	2.5	98.9 (2.3)	97.7 (0.6)	2.5	94.7 (2.4)	94.7 (4.9)	2.5	106.1 (2.1)	93.0 (1.6)
393	Pyraclonil	2.5	92.5 (1.4)	92.2 (1.7)	2.5	104.4 (2.1)	89.6 (2.5)	2.5	97.5 (4.4)	106.5 (2.2)	2.5	86.9 (3.9)	96.4 (2.8)	5.0	103.5 (0.6)	103.3 (2.0)
394	Pyraclostrobin	2.5	103.9 (5.3)	103.6 (1.7)	2.5	100.9 (1.4)	99.7 (5.8)	2.5	104.2 (0.1)	103.9 (1.4)	2.5	105.3 (4.5)	104.1 (1.3)	2.5	86.3 (3.8)	95.5 (3.8)
395	Pyraflufen-ethyl	2.5	N.D.	45.7 (2.0)	2.5	89.6 (1.3)	87.9 (3.7)	2.5	88.3 (0.8)	90.5 (1.0)	2.5	117.4 (1.6)	92.3 (2.7)	2.5	112.8 (2.3)	92.8 (1.5)
396	Pyraziflumid	5.0	81.3 (5.0)	92.0 (1.6)	2.5	104.4 (0.5)	86.5 (2.7)	2.5	107.3 (1.2)	105.6 (2.2)	2.5	93.8 (3.5)	93.3 (1.5)	2.5	101.6 (2.1)	88.2 (1.1)
397	Pyrazosulfuron-ethyl	2.5	88.6 (7.6)	97.2 (4.9)	2.5	78.2 (1.5)	90.5 (4.9)	2.5	76.5 (2.4)	93.5 (3.5)	2.5	81.1 (3.3)	93.1 (3.0)	2.5	75.7 (2.4)	96.4 (3.7)
398	Pyrazoxyfen	2.5	92.8 (1.7)	108.2 (1.1)	2.5	104.7 (1.6)	101.7 (3.7)	2.5	94.2 (3.9)	102.7 (1.1)	2.5	108.0 (1.5)	94.3 (2.5)	2.5	96.5 (1.8)	92.2 (1.8)
399	Pyribencarb E	2.5	110.9 (3.5)	109.0 (1.7)	2.5	103.9 (1.3)	95.3 (3.1)	2.5	106.0 (1.3)	99.6 (1.7)	2.5	110.2 (2.7)	107.3 (1.2)	5.0	102.0 (0.9)	103.1 (3.4)
400	Pyribencarb Z (KIE-9749)	2.5	116.5 (3.0)	107.7 (1.8)	2.5	108.9 (0.8)	95.2 (5.6)	2.5	116.1 (2.2)	92.7 (1.7)	2.5	116.4 (3.0)	93.1 (31.8)	2.5	108.1 (1.3)	81.6 (28.2)
401	Pyribenzoxim	2.5	88.1 (2.9)	107.6 (1.4)	2.5	99.9 (3.7)	99.1 (3.5)	2.5	93.5 (2.2)	90.7 (2.0)	2.5	93.1 (5.2)	109.0 (3.3)	2.5	101.9 (1.5)	112.7 (2.3)
402	Pyributicarb	2.5	105.7 (4.0)	93.7 (2.6)	2.5	107.1 (4.9)	98.5 (3.2)	2.5	89.6 (2.2)	101.2 (2.0)	2.5	100.1 (1.2)	99.0 (3.9)	5.0	57.8 (2.3)	71.3 (1.9)
403	Pyridaben	2.5	111.7 (1.8)	106.8 (3.7)	2.5	89.3 (3.7)	96.1 (6.7)	2.5	97.1 (0.9)	96.8 (2.5)	2.5	94.0 (18.4)	102.1 (15.1)	2.5	56.3 (2.0)	64.1 (3.1)
404	Pyridalyl	2.5	106.8 (8.5)	99.7 (2.1)	2.5	105.0 (5.6)	95.8 (3.3)	2.5	99.9 (7.8)	91.2 (1.5)	2.5	106.1 (4.3)	92.4 (1.4)	2.5	41.4 (3.9)	47.8 (2.7)
405	Pyridaphenthion	2.5	90.0 (1.7)	107.7 (3.0)	2.5	105.3 (1.8)	98.2 (4.1)	2.5	102.5 (0.6)	100.7 (1.5)	2.5	96.9 (3.7)	97.2 (2.0)	2.5	98.9 (0.8)	90.8 (1.8)
406	Pyrifenox	5.0	100.7 (5.3)	91.4 (1.9)	5.0	104.7 (1.8)	86.3 (4.1)	2.5	99.6 (1.8)	86.6 (1.7)	2.5	98.4 (3.0)	91.0 (1.6)	2.5	98.8 (2.2)	78.7 (1.4)
407	Pyrifluquinazon	2.5	82.4 (1.7)	104.2 (1.0)	2.5	98.1 (0.4)	96.7 (2.9)	2.5	104.7 (2.4)	100.2 (1.8)	2.5	104.4 (0.8)	101.1 (3.4)	2.5	96.8 (0.6)	94.5 (1.1)
408	Pyriftalid	5.0	107.7 (5.3)	95.8 (1.5)	2.5	87.5 (1.2)	98.7 (3.6)	5.0	88.5 (0.8)	93.9 (3.8)	2.5	114.0 (10.7)	107.2 (3.2)	2.5	104.7 (1.8)	92.1 (0.8)
409	Pyrimethanil	2.5	97.4 (3.6)	104.4 (0.4)	2.5	82.8 (5.1)	96.3 (7.0)	2.5	89.4 (1.0)	101.6 (1.9)	2.5	107.4 (3.1)	96.0 (1.6)	2.5	91.7 (2.6)	90.0 (2.1)
410	Pyrimethanil-5-hydroxy	5.0	99.7 (4.9)	90.4 (1.6)	2.5	96.4 (3.5)	86.4 (9.0)	2.5	101.1 (5.3)	98.9 (1.6)	2.5	92.1 (4.9)	82.8 (1.4)	2.5	N.D.	24.7 (4.1)
411	Pyrimidifen	5.0	90.9 (8.7)	87.9 (3.2)	2.5	95.5 (7.2)	98.1 (5.3)	2.5	96.6 (3.6)	101.6 (1.5)	2.5	98.9 (3.0)	96.3 (2.3)	2.5	59.7 (4.0)	62.7 (7.2)
412	Pyriminobac-methyl	2.5	110.5 (5.2)	95.7 (0.9)	2.5	81.2 (1.1)	86.2 (5.4)	2.5	91.9 (12.1)	93.3 (1.7)	2.5	76.8 (5.5)	107.3 (1.3)	2.5	102.8 (0.9)	91.9 (1.8)
413	Pyrimisulfan	2.5	103.0 (15.9)	95.6 (8.8)	2.5	82.9 (3.0)	90.6 (11.5)	2.5	91.6 (1.3)	96.4 (4.5)	2.5	96.6 (3.7)	94.8 (3.3)	2.5	96.2 (2.9)	104.7 (4.1)
414	Pyriofenone	2.5	91.5 (2.8)	109.1 (1.7)	2.5	105.3 (2.6)	100.1 (4.6)	2.5	98.8 (2.6)	104.2 (0.9)	2.5	98.6 (2.3)	87.1 (5.1)	2.5	86.9 (1.4)	81.8 (0.9)
415	Pyriproxyfen	2.5	102.1 (3.0)	101.1 (3.7)	2.5	99.8 (4.6)	97.3 (6.2)	2.5	105.1 (2.6)	101.3 (0.2)	2.5	94.3 (2.5)	95.9 (2.5)	2.5	58.7 (1.6)	73.1 (3.2)
416	Pyroquilon	2.5	94.9 (6.4)	107.1 (0.4)	2.5	106.8 (1.5)	89.4 (5.9)	5.0	101.7 (0.5)	92.2 (0.5)	2.5	106.9 (4.4)	91.5 (1.0)	2.5	95.0 (2.4)	100.8 (2.6)
417	Quinalphos	2.5	90.7 (2.0)	91.8 (1.4)	2.5	79.0 (3.6)	87.2 (4.3)	2.5	77.4 (1.5)	87.9 (3.5)	2.5	85.7 (6.1)	103.8 (4.8)	2.5	92.3 (2.2)	94.3 (2.4)
418	Quinclorac methyl ester	2.5	96.4 (3.2)	97.6 (1.0)	2.5	93.6 (2.3)	101.8 (4.7)	2.5	103.9 (0.9)	91.6 (1.8)	2.5	117.0 (3.7)	104.2 (1.7)	2.5	99.8 (2.5)	95.0 (1.0)
419	Quinoclamine	2.5	89.2 (6.8)	104.2 (1.3)	2.5	106.8 (1.3)	89.5 (6.3)	2.5	104.0 (2.7)	89.6 (1.2)	2.5	105.8 (3.8)	98.7 (1.9)	2.5	52.9 (4.4)	76.9 (4.7)
420	Quinoxyfen	2.5	93.9 (3.2)	102.3 (0.9)	2.5	90.2 (5.2)	93.4 (5.3)	2.5	78.1 (0.8)	89.3 (2.1)	2.5	102.0 (3.0)	87.3 (2.2)	2.5	60.4 (1.1)	57.5 (2.8)
421	Quizalofop-ethyl	2.5	69.7 (2.6)	105.5 (2.3)	2.5	103.8 (7.4)	87.0 (2.6)	5.0	94.3 (1.5)	95.1 (1.3)	2.5	106.7 (7.1)	98.6 (2.7)	2.5	84.6 (3.1)	90.2 (2.0)
422	Resmethrin	2.5	83.8 (1.6)	97.1 (2.4)	2.5	92.4 (4.5)	95.6 (2.3)	2.5	75.8 (2.3)	87.1 (1.6)	2.5	79.3 (3.6)	70.7 (3.4)	2.5	48.5 (1.5)	63.2 (3.1)
423	Rimsulfuron	2.5	67.5 (27.0)	68.1 (17.6)	2.5	53.2 (6.7)	70.2 (34.7)	2.5	53.4 (1.7)	58.8 (6.6)	2.5	73.7 (19.5)	74.2 (10.1)	2.5	38.9 (19.6)	74.9 (17.3)
424	Saflufenacil	2.5	87.5 (14.0)	86.2 (7.6)	2.5	76.0 (4.1)	83.6 (16.1)	5.0	71.9 (4.7)	89.7 (4.1)	2.5	86.3 (8.0)	88.9 (4.7)	2.5	75.2 (7.5)	92.0 (7.1)
425	Sedaxane	5.0	106.8 (4.1)	94.0 (2.0)	2.5	105.0 (1.8)	84.9 (4.5)	2.5	96.4 (0.9)	90.3 (0.6)	2.5	115.6 (4.4)	95.3 (2.2)	2.5	108.5 (0.8)	107.9 (1.8)
426	Sethoxydim	2.5	75.5 (5.1)	86.5 (2.9)	2.5	83.1 (1.9)	86.1 (5.7)	2.5	89.8 (1.1)	92.3 (2.4)	2.5	80.8 (5.5)	83.2 (1.9)	5.0	47.0 (6.5)	71.6 (3.2)
427	Simazine	5.0	106.8 (6.6)	94.8 (1.4)	2.5	107.2 (1.6)	86.4 (7.1)	2.5	110.1 (2.6)	90.4 (2.0)	2.5	104.8 (2.5)	90.6 (2.4)	2.5	92.1 (1.2)	98.3 (1.8)
428	Simeconazole	2.5	101.1 (3.6)	95.4 (1.8)	2.5	80.0 (4.6)	97.0 (3.0)	2.5	78.8 (3.9)	101.6 (1.3)	2.5	94.7 (3.1)	108.2 (1.5)	2.5	112.6 (3.5)	91.2 (2.5)
429	Simetryn	2.5	104.7 (2.5)	104.1 (0.8)	2.5	92.2 (1.8)	97.8 (5.4)	2.5	90.3 (1.3)	99.6 (3.0)	2.5	93.7 (4.8)	99.9 (0.3)	2.5	105.0 (1.8)	96.9 (1.9)
430	Spinetoram-J	2.5	96.8 (11.2)	102.0 (2.9)	2.5	98.1 (1.3)	102.2 (3.8)	2.5	104.0 (1.6)	105.1 (1.0)	2.5	101.4 (1.3)	111.8 (2.2)	2.5	74.6 (3.1)	91.7 (5.8)
431	Spinetoram-L	2.5	102.5 (2.2)	103.3 (0.7)	2.5	96.1 (4.6)	98.2 (4.3)	2.5	100.4 (1.8)	100.2 (0.1)	2.5	95.5 (2.0)	98.8 (3.0)	2.5	79.3 (0.4)	83.9 (3.0)
432	Spinosyn A	2.5	101.4 (3.7)	102.1 (1.7)	2.5	95.9 (2.2)	99.4 (5.1)	2.5	99.6 (1.4)	103.3 (1.6)	2.5	99.2 (1.7)	103.3 (3.5)	2.5	77.7 (4.7)	91.2 (2.2)
433	Spinosyn D	2.5	101.7 (6.5)	103.3 (2.4)	2.5	99.6 (1.9)	99.8 (4.7)	2.5	103.4 (1.3)	103.1 (0.6)	2.5	101.5 (5.3)	106.6 (2.4)	2.5	73.5 (3.1)	89.2 (4.9)
434	Spirodiclofen	2.5	75.1 (0.3)	90.5 (1.4)	2.5	84.9 (3.0)	92.4 (4.7)	2.5	96.3 (1.6)	101.8 (1.7)	2.5	82.4 (10.2)	80.1 (4.7)	2.5	68.0 (1.5)	74.7 (1.7)
435	Spiromesifen	20	N.D.	104.8 (1.0)	20	N.D.	93.4 (1.2)	20	N.D.	98.8 (0.4)	20	N.D.	97.5 (2.6)	25	N.D.	76.5 (1.6)
436	Spirotetramat	2.5	100.5 (5.5)	101.9 (2.5)	2.5	96.1 (1.5)	97.2 (6.0)	2.5	101.5 (1.8)	98.4 (3.1)	2.5	62.1 (1.3)	68.1 (4.4)	2.5	90.2 (0.6)	93.7 (2.0)
437	Spiroxamine	2.5	109.4 (3.1)	105.5 (0.3)	2.5	105.9 (2.8)	97.4 (4.2)	2.5	97.2 (1.0)	100.0 (1.4)	2.5	107.8 (3.1)	100.9 (2.1)	2.5	62.1 (0.6)	72.1 (3.5)
438	Sulfentrazone	2.5	90.1 (6.8)	90.7 (5.9)	2.5	82.9 (3.6)	92.1 (13.3)	2.5	93.5 (2.8)	97.3 (3.6)	2.5	95.3 (6.2)	97.6 (4.3)	5.0	63.5 (12.0)	85.5 (1.0)
439	Sulfotep	2.5	104.0 (2.7)	108.1 (1.4)	2.5	87.1 (5.6)	97.8 (5.9)	2.5	95.2 (3.7)	100.6 (2.5)	2.5	78.7 (3.9)	102.2 (3.3)	2.5	96.3 (2.9)	89.1 (3.3)
440	Sulfoxaflor	5.0	106.7 (4.5)	94.8 (2.7)	5.0	100.8 (1.3)	92.1 (7.7)	2.5	96.2 (3.3)	90.9 (3.7)	2.5	107.9 (3.0)	97.5 (1.4)	2.5	100.4 (1.8)	93.3 (3.0)
441	Sulprofos	2.5	88.9 (2.0)	101.9 (0.4)	2.5	97.2 (4.2)	97.1 (2.3)	2.5	104.0 (2.7)	102.4 (1.6)	2.5	91.5 (4.3)	92.3 (4.6)	2.5	63.6 (0.7)	74.8 (2.0)
442	TCMTB	2.5	94.3 (4.0)	103.0 (1.7)	2.5	104.8 (1.0)	91.5 (17.1)	2.5	103.6 (4.2)	101.9 (2.1)	2.5	29.9 (2.0)	34.5 (2.1)	2.5	23.7 (5.0)	49.4 (4.7)
443	Tebuconazole	2.5	101.2 (2.1)	95.9 (1.0)	5.0	104.9 (2.4)	90.6 (3.8)	5.0	91.4 (3.8)	92.7 (1.3)	2.5	95.9 (2.7)	93.7 (2.0)	2.5	111.0 (1.0)	91.8 (1.2)
444	Tebufenozide	2.5	106.6 (4.2)	104.6 (1.1)	2.5	102.5 (3.3)	106.5 (1.9)	2.5	100.0 (0.6)	105.0 (2.8)	2.5	116.5 (1.3)	111.7 (2.3)	2.5	98.0 (2.5)	100.1 (1.2)
445	Tebufenpyrad	2.5	97.2 (6.0)	92.9 (1.6)	2.5	90.4 (5.4)	97.2 (3.5)	2.5	89.9 (2.2)	101.3 (1.5)	2.5	110.4 (1.7)	95.6 (2.3)	2.5	84.8 (4.8)	80.3 (5.6)
446	Tebufloquin	2.5	91.4 (0.9)	104.1 (1.5)	2.5	103.0 (2.1)	97.2 (6.8)	2.5	95.6 (3.4)	91.1 (1.5)	2.5	97.7 (1.7)	96.8 (3.5)	2.5	78.3 (2.0)	88.0 (2.5)
447	Tebufloquin metabolite (M1)	5.0	106.7 (4.2)	92.9 (1.0)	2.5	107.7 (3.9)	98.1 (6.0)	2.5	101.6 (1.5)	96.1 (1.8)	2.5	91.5 (3.5)	102.9 (2.3)	2.5	98.3 (2.0)	89.7 (2.7)
448	Tebupirimfos	2.5	88.0 (10.5)	101.7 (3.0)	2.5	86.7 (5.6)	94.2 (2.1)	2.5	88.6 (0.8)	98.9 (0.8)	2.5	104.9 (5.2)	94.3 (4.3)	2.5	73.3 (2.0)	74.7 (4.8)
449	Tebuthiuron	5.0	103.7 (3.8)	92.3 (1.9)	2.5	107.3 (2.8)	86.5 (4.1)	2.5	90.7 (1.6)	91.1 (1.0)	2.5	104.4 (5.2)	90.5 (0.4)	5.0	59.1 (2.0)	70.7 (4.8)
450	Teflubenzuron	2.5	89.2 (6.4)	106.1 (0.1)	2.5	94.2 (3.0)	97.0 (3.2)	2.5	98.5 (1.9)	85.8 (2.2)	2.5	106.3 (7.4)	102.2 (0.6)	2.5	94.1 (8.6)	90.3 (1.0)
451	Tefuryltrione	2.5	66.5 (67.0)	26.3 (49.6)	2.5	9.9 (44.8)	31.3 (115.4)	2.5	30.7 (2.5)	25.9 (21.4)	2.5	68.8 (51.6)	38.5 (38.3)	2.5	8.0 (21.8)	19.2 (65.3)
452	TEPP	20.0	N.D.	94.7 (1.3)	2.5	85.8 (5.1)	91.7 (3.7)	2.5	95.3 (1.7)	101.6 (3.5)	20.0	N.D.	86.0 (2.3)	25.0	N.D.	88.9 (1.4)
453	Tepraloxydim	2.5	71.6 (14.3)	71.3 (9.1)	2.5	72.4 (3.3)	82.1 (19.5)	5.0	84.8 (9.0)	86.2 (10.2)	2.5	84.4 (17.1)	63.1 (5.7)	2.5	77.8 (2.1)	82.1 (10.5)
454	Terbacil	2.5	110.9 (1.5)	101.9 (1.5)	2.5	109.3 (1.5)	98.7 (1.9)	2.5	112.6 (0.4)	96.0 (4.0)	2.5	106.8 (1.6)	98.5 (3.2)	2.5	87.4 (3.8)	88.3 (3.9)
455	Terbufos	2.5	80.2 (20.1)	99.0 (3.7)	2.5	97.2 (5.0)	96.9 (3.3)	10.0	84.5 (3.5)	103.2 (2.1)	5.0	96.7 (12.2)	93.1 (7.2)	10.0	18.6 (35.0)	64.8 (8.8)
456	Terbufos oxon	2.5	93.0 (5.3)	88.0 (2.1)	2.5	107.0 (3.2)	99.0 (4.1)	2.5	104.4 (13.1)	98.0 (12.7)	2.5	109.0 (3.5)	94.5 (0.7)	2.5	102.9 (2.6)	90.8 (1.9)
457	Terbufos oxon sulfone	2.5	103.0 (6.0)	108.5 (4.8)	2.5	100.5 (2.9)	101.2 (2.8)	2.5	97.0 (1.5)	94.4 (4.9)	2.5	78.3 (3.0)	103.8 (1.8)	2.5	84.3 (5.4)	98.4 (2.8)
458	Terbufos oxon sulfoxide	2.5	106.6 (5.0)	100.9 (2.3)	2.5	99.6 (1.1)	94.8 (5.5)	2.5	98.1 (2.6)	103.2 (0.2)	2.5	99.3 (3.4)	105.5 (2.5)	2.5	99.1 (1.5)	98.1 (2.1)
459	Terbufos sulfone	5.0	103.4 (3.9)	93.5 (1.2)	2.5	103.9 (3.2)	88.7 (4.3)	2.5	109.4 (0.4)	93.8 (1.3)	2.5	95.0 (4.6)	107.2 (1.9)	2.5	104.9 (1.8)	92.4 (2.8)
460	Terbufos sulfoxide	2.5	101.5 (3.3)	94.0 (1.3)	2.5	107.6 (2.1)	100.8 (3.4)	2.5	93.1 (2.0)	93.5 (2.0)	2.5	82.8 (3.8)	106.1 (1.7)	2.5	105.3 (2.5)	95.4 (2.2)
461	Terbuthylazine	5.0	99.3 (7.8)	89.5 (2.5)	5.0	99.8 (1.0)	85.2 (9.3)	2.5	91.7 (1.4)	90.5 (1.0)	2.5	95.5 (3.8)	103.3 (2.7)	2.5	91.6 (2.8)	80.2 (5.0)
462	Terbutryn	2.5	104.3 (12.1)	101.1 (4.8)	2.5	87.2 (2.4)	97.7 (6.1)	2.5	85.5 (3.2)	98.3 (0.9)	2.5	106.9 (4.6)	98.8 (1.8)	2.5	89.8 (2.2)	90.9 (0.1)
463	Tetrachlorvinphos	5.0	96.5 (2.3)	94.4 (1.9)	5.0	80.8 (4.1)	88.3 (4.5)	2.5	71.5 (3.5)	89.5 (0.8)	2.5	73.7 (3.2)	93.0 (2.6)	2.5	102.9 (2.1)	90.0 (1.6)
464	Tetraconazole	2.5	91.4 (4.2)	96.1 (2.2)	2.5	79.5 (4.2)	98.9 (2.0)	2.5	83.6 (3.9)	103.6 (0.2)	2.5	84.3 (4.1)	107.8 (2.0)	2.5	107.8 (2.2)	93.1 (2.9)
465	Tetramethrin	2.5	94.9 (1.5)	93.3 (0.6)	2.5	81.2 (3.9)	89.2 (2.4)	2.5	95.7 (3.6)	90.1 (1.0)	2.5	79.4 (8.1)	97.8 (2.2)	2.5	102.6 (3.4)	93.1 (1.6)
466	Tetraniliprole	2.5	87.1 (2.5)	105.6 (2.4)	2.5	97.6 (1.3)	99.1 (3.0)	2.5	102.3 (2.6)	104.2 (0.4)	2.5	101.2 (6.2)	102.6 (3.1)	2.5	107.2 (1.7)	104.0 (1.9)
467	Thenylchlor	2.5	91.1 (3.1)	104.2 (0.9)	2.5	103.1 (4.3)	97.8 (4.7)	2.5	107.8 (2.9)	103.6 (1.1)	2.5	90.4 (3.2)	103.7 (1.8)	2.5	95.3 (2.0)	100.3 (1.2)
468	Thiabendazole	2.5	84.1 (5.5)	103.7 (2.1)	2.5	104.6 (2.1)	91.1 (12.1)	2.5	107.1 (2.2)	105.3 (5.4)	2.5	93.3 (4.6)	90.5 (4.0)	2.5	65.8 (3.3)	75.5 (6.5)
469	Thiacloprid	5.0	102.7 (1.9)	93.6 (2.1)	5.0	99.9 (3.3)	88.5 (7.5)	2.5	95.2 (1.6)	91.6 (2.8)	2.5	110.1 (2.4)	93.6 (0.4)	2.5	100.8 (0.4)	92.5 (4.1)
470	Thiamethoxam	5.0	102.5 (1.8)	95.6 (1.3)	2.5	105.3 (1.6)	89.9 (1.7)	2.5	101.8 (5.1)	84.2 (5.1)	2.5	101.3 (4.7)	89.8 (1.9)	2.5	53.3 (2.6)	52.4 (6.3)
471	Thiazopyr	5.0	107.5 (1.4)	93.2 (1.8)	5.0	98.6 (2.6)	89.3 (4.9)	2.5	92.6 (2.2)	90.5 (2.3)	2.5	92.8 (5.9)	93.3 (2.8)	2.5	89.7 (2.8)	81.9 (2.5)
472	Thidiazuron	2.5	110.8 (7.2)	88.7 (2.0)	2.5	93.0 (2.2)	92.7 (5.1)	10.0	92.0 (1.4)	100.2 (4.4)	2.5	100.6 (11.5)	86.3 (1.6)	5.0	94.4 (2.9)	90.1 (1.7)
473	Thifensulfuron-methyl	2.5	63.1 (10.0)	71.1 (7.0)	2.5	58.0 (3.7)	70.7 (21.9)	2.5	53.9 (1.9)	66.9 (8.1)	2.5	66.4 (7.8)	70.5 (8.1)	2.5	41.0 (7.2)	64.2 (12.4)
474	Thifluzamide	5.0	96.1 (4.6)	97.7 (2.2)	5.0	118.3 (0.6)	90.1 (2.3)	2.5	97.9 (3.1)	90.7 (3.2)	2.5	92.6 (5.8)	104.3 (1.0)	2.5	116.1 (2.7)	89.5 (3.2)
475	Thiobencarb	5.0	97.6 (3.2)	95.7 (1.3)	2.5	102.5 (3.9)	98.9 (4.1)	2.5	91.5 (1.0)	91.3 (1.6)	2.5	101.2 (2.1)	97.1 (2.6)	2.5	78.8 (1.0)	85.7 (1.1)
476	Thiometon	2.5	106.8 (3.3)	94.5 (1.2)	5.0	84.7 (11.4)	89.6 (5.2)	20.0	N.D.	108.8 (4.8)	2.5	97.2 (9.7)	80.7 (5.1)	10.0	92.1 (4.0)	105.6 (4.8)
477	Tiadinil	2.5	94.3 (3.0)	107.9 (1.0)	2.5	103.6 (1.7)	100.0 (3.7)	2.5	105.3 (3.3)	103.7 (0.9)	2.5	105.7 (3.0)	102.3 (2.4)	2.5	94.6 (4.8)	96.6 (1.8)
478	Tolclofos-methyl	2.5	96.7 (1.5)	106.3 (0.6)	2.5	89.5 (5.5)	96.9 (3.5)	2.5	91.2 (3.4)	99.6 (1.9)	2.5	84.7 (6.1)	97.4 (3.8)	2.5	87.8 (3.7)	82.1 (1.1)
479	Tolfenpyrad	2.5	88.6 (16.2)	92.0 (6.3)	2.5	94.5 (2.9)	98.4 (1.5)	2.5	101.4 (1.6)	101.3 (0.2)	2.5	97.4 (5.9)	93.4 (7.4)	2.5	65.0 (3.5)	72.6 (8.4)
480	Tralkoxydim	2.5	84.1 (8.3)	85.0 (4.0)	2.5	78.7 (2.1)	84.4 (10.2)	2.5	89.3 (0.7)	88.4 (1.5)	2.5	82.5 (1.7)	81.2 (3.1)	2.5	58.5 (1.9)	68.7 (2.9)
481	Triadimefon	5.0	100.1 (1.3)	100.3 (3.7)	5.0	84.0 (1.7)	90.4 (3.9)	5.0	96.1 (2.9)	93.2 (1.5)	2.5	90.8 (6.8)	106.1 (1.0)	5.0	111.2 (2.2)	91.4 (1.7)
482	Triadimenol	2.5	104.0 (10.1)	109.6 (3.4)	2.5	78.2 (4.8)	98.5 (0.8)	5.0	61.6 (9.1)	100.7 (0.4)	2.5	90.3 (5.9)	71.8 (3.7)	5.0	113.3 (6.7)	97.5 (1.3)
483	Triafamone	5.0	105.5 (2.6)	94.3 (2.2)	2.5	104.0 (1.0)	89.2 (5.3)	2.5	95.0 (1.0)	89.3 (0.3)	2.5	106.9 (2.4)	94.1 (2.4)	2.5	102.6 (1.1)	96.6 (2.4)
484	Tri-allate	2.5	94.9 (4.0)	105.5 (0.8)	2.5	96.5 (4.4)	94.0 (2.7)	2.5	95.8 (2.2)	97.3 (1.1)	2.5	83.2 (6.2)	89.5 (3.7)	2.5	60.0 (1.9)	63.3 (3.3)
485	Triasulfuron	2.5	59.0 (16.8)	67.9 (9.3)	2.5	49.8 (4.4)	65.8 (31.4)	5.0	48.3 (2.6)	62.8 (6.5)	2.5	66.9 (10.9)	68.4 (10.7)	2.5	37.2 (13.5)	54.3 (15.1)
486	Triazamate	5.0	100.9 (4.8)	93.9 (1.4)	2.5	105.2 (1.5)	99.2 (3.3)	2.5	94.6 (1.1)	93.7 (2.4)	2.5	81.1 (3.4)	102.0 (1.7)	2.5	96.8 (2.1)	89.2 (0.9)
487	Triazophos	5.0	91.6 (4.1)	96.0 (1.7)	5.0	101.8 (1.4)	85.6 (4.9)	2.5	95.5 (1.7)	102.0 (1.8)	2.5	120.7 (1.8)	94.1 (1.8)	2.5	105.4 (1.4)	87.1 (2.5)
488	Tribufos	5.0	103.1 (1.2)	97.2 (2.6)	2.5	90.3 (2.3)	96.0 (4.8)	2.5	103.2 (1.4)	98.8 (1.0)	2.5	85.2 (15.7)	86.2 (14.9)	2.5	57.0 (2.4)	58.7 (3.3)
489	Tricyclazole	2.5	101.0 (12.1)	88.3 (2.5)	2.5	107.6 (1.4)	96.5 (4.0)	2.5	91.8 (3.1)	102.2 (1.3)	2.5	100.7 (4.4)	93.1 (0.3)	2.5	84.9 (1.1)	95.9 (4.1)
490	Trifloxystrobin	2.5	96.5 (2.6)	107.4 (0.9)	2.5	107.2 (4.2)	96.6 (5.2)	2.5	93.6 (1.7)	100.4 (2.1)	2.5	111.3 (1.6)	100.5 (2.8)	2.5	94.1 (1.6)	95.1 (2.1)
491	Trifloxysulfuron	2.5	60.7 (31.5)	58.6 (23.0)	2.5	39.1 (11.2)	59.6 (40.1)	2.5	48.9 (2.7)	62.9 (8.6)	2.5	61.1 (25.6)	64.7 (10.9)	2.5	39.1 (7.6)	64.6 (27.9)
492	Triflumizole	2.5	98.4 (3.7)	107.6 (1.6)	5.0	109.8 (2.0)	90.5 (3.2)	2.5	101.1 (1.8)	99.0 (0.3)	2.5	116.6 (2.9)	98.6 (1.6)	2.5	94.8 (1.4)	88.3 (3.6)
493	Triflumuron	2.5	92.1 (6.3)	106.6 (0.9)	2.5	102.6 (2.5)	90.1 (2.7)	2.5	94.0 (1.8)	93.3 (0.6)	2.5	104.2 (0.7)	93.6 (2.6)	2.5	95.6 (2.5)	89.4 (1.6)
494	Triflusulfuron metabolite (IN-M7222)	2.5	127.9 (4.8)	105.2 (3.7)	2.5	122.7 (2.8)	90.3 (7.5)	2.5	110.2 (3.4)	96.1 (3.8)	2.5	110.7 (5.0)	95.6 (4.1)	2.5	74.7 (1.3)	75.3 (6.4)
495	Trinexapac-ethyl	2.5	29.2 (79.8)	32.6 (44.1)	2.5	0.4 (173.2)	37.6 (98.8)	2.5	17.5 (8.6)	34.4 (18.7)	2.5	32.8 (54.0)	46.2 (29.1)	2.5	1.7 (77.5)	31.0 (49.3)
496	Triticonazole	2.5	94.2 (0.5)	105.3 (1.7)	2.5	103.7 (4.6)	96.5 (2.3)	2.5	96.6 (3.4)	91.2 (1.6)	2.5	108.9 (2.2)	102.7 (1.6)	2.5	103.9 (1.0)	94.8 (2.8)
497	Tritosulfuron metabolite (M635H004)	2.5	88.9 (5.2)	103.5 (0.9)	2.5	97.3 (7.4)	101.4 (4.2)	2.5	94.1 (1.6)	99.9 (1.4)	2.5	111.2 (3.4)	104.9 (1.7)	2.5	98.7 (1.1)	96.3 (2.3)
498	TZ-1E (picarbutrazox metabolite)	2.5	91.0 (2.8)	106.4 (2.4)	2.5	100.7 (0.6)	98.4 (3.8)	5.0	95.3 (3.4)	99.6 (2.2)	2.5	87.7 (3.9)	96.2 (2.9)	2.5	103.2 (2.9)	99.9 (0.5)
499	Uniconazole	2.5	94.3 (3.0)	102.6 (1.3)	2.5	104.3 (3.5)	98.1 (3.6)	2.5	88.3 (1.7)	87.2 (0.2)	2.5	110.9 (4.7)	101.6 (1.4)	2.5	98.7 (1.6)	103.5 (0.7)
500	Valifenalate	2.5	107.5 (6.3)	95.1 (1.5)	2.5	101.7 (2.1)	89.8 (3.2)	2.5	90.9 (4.2)	92.6 (1.6)	2.5	94.8 (6.0)	95.0 (2.4)	2.5	107.7 (1.8)	97.5 (1.4)
501	Vamidothion	2.5	99.8 (4.0)	90.9 (1.3)	2.5	101.5 (3.2)	99.7 (9.6)	2.5	93.0 (2.3)	90.8 (5.4)	2.5	102.1 (3.3)	89.3 (5.9)	2.5	96.1 (2.3)	104.3 (2.8)
502	Vernolate	2.5	98.8 (2.8)	103.0 (1.2)	2.5	95.2 (1.4)	92.4 (3.9)	2.5	99.1 (1.0)	100.7 (0.6)	2.5	96.0 (1.7)	95.1 (2.3)	2.5	71.1 (1.1)	77.0 (2.0)
503	XMC	2.5	108.2 (1.9)	96.8 (0.6)	2.5	100.5 (4.4)	90.9 (4.6)	5.0	105.2 (3.0)	105.5 (0.6)	2.5	99.1 (8.9)	103.4 (2.5)	2.5	95.5 (1.2)	97.9 (3.2)
504	Zoxamide	2.5	92.8 (2.7)	95.5 (1.3)	2.5	86.8 (3.5)	99.8 (4.8)	2.5	90.6 (1.8)	91.3 (1.3)	5.0	105.3 (3.3)	89.2 (3.9)	2.5	106.4 (1.5)	97.8 (1.8)

^1^ Relative standard deviation. ^2^ Not detectable.

**Table 3:** Distribution of the limits of quantitation (LOQs) for multiresidues in representative crops.

LOQ (μg/kg)	Number of Pesticides (Percentage)				
	Potato	Cabbage	Mandarin	Brown Rice	Soybean
2.5	413 (81.9%)	415 (82.2%)	418 (82.9%)	440 (87.1%)	404 (80.2%)
5.0	75 (14.9%)	79 (15.7%)	47 (9.3%)	50 (9.9%)	79 (15.6%)
10	1 (0.2%)	4 (0.8%)	23 (4.6%)	3 (0.6%)	9 (1.8%)
20–25	15 (3.0%)	6 (1.2%)	16 (3.2%)	11 (2.2%)	12 (2.4%)
Sum	504 (100%)	504 (100%)	504 (100%)	504 (100%)	504 (100%)

**Table 4:** Distribution of correlation coefficients (*r*^2^) for multiresidues in representative crops.

*r* ^2^	Number of Pesticides (Percentage)				
	Potato	Cabbage	Mandarin	Brown Rice	Soybean
≥0.990	412 (81.7%)	406 (80.6%)	429 (85.1%)	428 (84.9%)	392 (77.8%)
0.980–0.990	91 (18.0%)	98 (19.4%)	73 (14.5%)	76 (15.0%)	109 (21.6%)
0.940–0.980	1 (0.2%)	0 (0.0%)	2 (0.4%)	0 (0.0%)	3 (0.6%)
Sum	504 (100%)	504 (100%)	504 (100%)	504 (100%)	504 (100%)

**Table 5:** Distribution of recovery ranges at fortification levels of 10 and 100 µg/kg for 504 pesticide multiresidues in representative crops.

Recovery (%)	RSD ^1^ (%)	Number of Pesticides (Percentage)									
		Potato		Cabbage		Mandarin		Brown Rice		Soybean	
		10 μg/kg	100 μg/kg	10 μg/kg	100 μg/kg	10 μg/kg	100 μg/kg	10 μg/kg	100 μg/kg	10 μg/kg	100 μg/kg
N.D. ^2^		17 (3.4%)	0 (0.0%)	10 (2.0%)	0 (0.0%)	22 (4.4%)	0 (0.0%)	13 (2.6%)	0 (0.0%)	20 (4.0%)	0 (0.0%)
<30	all	7 (1.4%)	9 (1.8%)	11 (2.2%)	6 (1.2%)	6 (1.2%)	9 (1.8%)	10 (2.0%)	9 (1.8%)	11 (2.2%)	15 (3.0%)
30–70	≤20	8 (1.6%)	7 (1.4%)	17 (3.4%)	2 (0.4%)	22 (4.4%)	18 (3.6%)	9 (1.8%)	13 (2.6%)	74 (14.7%)	41 (8.1%)
	>20	14 (2.8%)	11 (2.2%)	1 (0.2%)	19 (3.8%)	1 (0.2%)	1 (0.2%)	9 (1.8%)	6 (1.2%)	1 (0.2%)	12 (2.4%)
70–120	≤20	437 (86.7%)	475 (94.2%)	457 (90.7%)	456 (90.5%)	449 (89.1%)	476 (94.4%)	453 (89.9%)	475 (94.2%)	394 (78.2%)	434 (86.1%)
	>20	14 (2.8%)	2 (0.4%)	1 (0.2%)	21 (4.2%)	4 (0.8%)	0 (0.0%)	6 (1.2%)	1 (0.2%)	0 (0.0%)	2 (0.4%)
120–140	≤20	7 (1.4%)	0 (0.0%)	7 (1.4%)	0 (0.0%)	0 (0.0%)	0 (0.0%)	4 (0.8%)	0 (0.0%)	4 (0.8%)	0 (0.0%)
Sum		504 (100%)	504 (100%)	504 (100%)	504 (100%)	504 (100%)	504 (100%)	504 (100%)	504 (100%)	504 (100%)	504 (100%)

^1^ Relative standard deviation. ^2^ Not detectable.

**Table 6:** Residue analysis of pesticides in agricultural products using the QTOF method.

Crop	Numberof Sample	Compound Name	Fit Score	Residue (μg/kg)	MRL (μg/kg)
Cabbage	3	Cyazofamid	100	164.1	700
		Fluopicolide	97.6	13.8	300
		Fluxapyroxad	100	48.1	1000
		Propamocarb	100	28.3	1000
		Pyraclostrobin	100	136.5	2000
Mandarin	2	Cyprodinil	100	26.7	1000
		Fenpropathrin	100	96.0	5000
		Fluxametamide	100	61.6	300
		Lufenuron	98.5	43.9	500
	3	Buprofezin	100	12.3	500
		Cyprodinil	100	36.5	1000
		Flonicamid	99.6	17.0	1000
		Indoxacarb	70.8	15.5	500
		Lufenuron	98.6	38.9	500
		Pyridaben	100	15.2	2000
		Pyriproxyfen	99	24.2	700
	4	Buprofezin	99.4	27.2	500
		Carbendazim	100	339.3	5000
		Flonicamid	99.7	59.2	1000
		Sulfoxaflor	100	44.8	1000
Rice	4	Fenobucarb	100	17.1	500
	6	Fenoxanil	99.3	55.7	1000
	11	Acetamiprid	100	25.6	300
	22	Fenoxanil	99.3	41.3	1000
	23	Acetamiprid	98.6	36.0	300
		Tricyclazole	100	119.1	700
	26	Propiconazole	93.7	33.4	700

## Data Availability

The original contributions presented in the study are included in the article/Supplementary Materials; further inquiries can be directed to the corresponding authors.

## Supplementary Materials

The following supporting information can be downloaded at: https://www.mdpi.com/article/10.3390/foods13213503/s1, Table S1: Molecular formulae, monoisotopic masses, ionization types, theoretical *m*/*z*, and retention times (t_R_) for 504 pesticide multiresidues using UHPLC-QTOF; Table S2: Linear ranges and correlation coefficients (*r*^2^) for 504 pesticide multiresidues in five representative crops; Table S3: Recovery ranges (100 µg/kg) of pesticides exhibiting generally low recovery rates in the representative crops and their chemical moieties.
