# Supplementary material for: Simultaneous Analysis of 504 Pesticide Multiresidues in Crops Using UHPLC-QTOF at MS1 and MS2 Levels
Source: Foods. 2024 Oct 31;13(21):3503. doi: 10.3390/foods13213503 (PMC11545108; doi:10.3390/foods13213503)
Supplement: Supplementary file 1 [file foods-13-03503-s001.zip › foods-3279128-supplementary.pdf]

Supplementary Materials

Table S1. Molecular formulae, monoisotopic masses, ionization types, theoretical m/z, and retention times (t_R) for 504 pesticide multiresidues using UHPLC-QTOF.

No.	Compound name	Molecular formula formula	Monoisotopic mass	Ionization type	Theoretical m/z	t_R
1	2,3,5-Trimethacarb	C11H15NO2	193.11028	[M+H]+	194.11756	6.58
2	3,4,5-Trimethacarb	C11H15NO2	193.11028	[M+H]+	194.11756	6.68
3	Acetamiprid	C10H11ClN4	222.06722	[M+H]+	223.07450	3.60
4	Acetochlor	C14H20ClNO2	269.11826	[M+H]+	270.12553	9.29
5	Acibenzolar acid	C7H4N2O2S	179.99935	[M-H]-	178.99207	3.95
6	Acibenzolar-S-methyl	C8H6N2OS2	209.99216	[M+H]+	210.99943	7.97
7	Acrinathrin	C26H21F6NO5	541.13239	[M+NH4]+	559.16622	14.19
8	AD-67 (MON-4660)	C10H15Cl2NO2	251.04799	[M+H]+	252.05526	6.83
9	Alachlor	C14H20ClNO2	269.11826	[M+H]+	270.12553	9.30
10	Aldoxycarb (Aldicarb sulfone)	C7H14N2O4S	222.06743	[M+NH4]+	240.10126	3.05
11	Allethrin	C19H26O3	302.18820	[M+H]+	303.19547	12.61
12	Allidochlor	C8H12ClNO	173.06074	[M+H]+	174.06802	4.62
13	Ametoctradin	C15H25N5	275.21100	[M+H]+	276.21827	11.16
14	Ametryn	C9H17N5S	227.12047	[M+H]+	228.12774	6.86
15	Amisulbrom	C13H13BrFN5O4S2	464.95764	[M+H]+	465.96492	11.84
16	Anilofos	C13H19ClNO3PS2	367.02325	[M+H]+	368.03053	10.18
17	Aramite	C15H23ClO4S	334.10056	[M+NH4]+	352.13439	12.58
18	Aspon	C12H28O5P2S2	378.08535	[M+H]+	379.09262	13.63
19	Asulam	C8H10N2O4S	230.03613	[M+H]+	231.04341	2.98
20	Atrazine	C8H14ClN5	215.09377	[M+H]+	216.10105	6.38
21	Avermectin B1a	C48H72O14	872.49221	[M+NH4]+	890.52604	14.66
22	Azaconazole	C12H11Cl2N3O2	299.02283	[M+H]+	300.03011	6.70
23	Azamethiphos	C9H10ClN2O5PS	323.97366	[M+H]+	324.98094	4.77
24	Azimsulfuron	C13H16N10O5S	424.10259	[M+H]+	425.10986	6.24
25	Azinphos-ethyl	C12H16N3O3PS2	345.03707	[M+H]+	346.04435	8.98
26	Azoxystrobin	C22H17N3O5	403.11682	[M+H]+	404.12410	7.29
27	Bendiocarb	C11H13NO4	223.08446	[M+H]+	224.09174	5.06
28	Benfuresate	C12H16O4S	256.07693	[M+NH4]+	274.11076	6.74
29	Benodanil	C13H10INO	322.98071	[M+H]+	323.98799	6.13
30	Bensulide	C14H24NO4PS3	397.06051	[M+H]+	398.06779	9.68
31	Benthiavalicarb-isopropyl	C18H24FN3O3S	381.15224	[M+H]+	382.15952	8.38
32	Benzobicyclon	C22H19ClO4S2	446.04133	[M+H]+	447.04861	8.15
33	Benzoximate	C18H18ClNO5	363.08735	[M+H]+	364.09463	10.92
34	Benzoylprop-ethyl	C18H17Cl2NO3	365.05855	[M+H]+	366.06583	10.50
35	Benzpyrimoxan	C16H15F3N2O3	340.10348	[M+H]+	341.11075	9.54
36	Benzyladenine	C12H11N5	225.10145	[M+H]+	226.10872	4.36
37	Bifenox	C14H9Cl2NO5	340.98578	[M+NH4]+	359.01961	11.18
38	Bifenthrin	C23H22ClF3O2	422.12604	[M+NH4]+	440.15987	15.54
39	Bispyribac	C19H17N4NaO8	452.09441	[M+H]+	453.10169	7.91
40	Bistrifluron	C16H7ClF8N2O2	446.00683	[M-H]-	444.99955	13.32
41	Bitertanol	C20H23N3O2	337.17903	[M+H]+	338.18630	10.82
42	Bixafen	C18H12Cl2F3N3O	413.03095	[M+H]+	414.03823	9.80
43	Boscalid	C18H12Cl2N2O	342.03267	[M+H]+	343.03995	7.97
44	Bromacil	C9H13BrN2O2	260.01604	[M-H]-	259.00876	5.16
45	Bromobutide	C15H22BrNO	311.08848	[M+H]+	312.09575	9.20
46	Bupirimate	C13H24N4O3S	316.15691	[M+H]+	317.16419	9.16

No.	Compound name	Molecular formula formula	Monoisotopic mass	Ionization type	Theoretical m/z	t_R
47	Buprofezin	C16H23N3OS	305.15618	[M+H]+	306.16346	12.39
48	Butachlor	C17H26ClNO2	311.16521	[M+H]+	312.17248	12.65
49	Butafenacil	C20H18ClF3N2O6	474.08055	[M+NH4]+	492.11438	8.86
50	Butocarboxim	C7H14N2O2S	190.07760	[M+Na]+	213.06682	4.31
51	Butralin	C14H21N3O4	295.15321	[M+H]+	296.16048	13.68
52	Butylate	C11H23NOS	217.15004	[M+H]+	218.15731	12.08
53	Cadusafos	C10H23O2PS2	270.08771	[M+H]+	271.09499	11.27
54	Cafenstrole	C16H22N4O3S	350.14126	[M+H]+	351.14854	8.47
55	Carbaryl	C12H11NO2	201.07898	[M+H]+	202.08626	5.46
56	Carbendazim	C9H9N3O2	191.06948	[M+H]+	192.07675	3.32
57	Carbetamide	C12H16N2O3	236.11609	[M+H]+	237.12337	4.66
58	Carbofuran	C12H15NO3	221.10519	[M+H]+	222.11247	5.11
59	Carbofuran-3-hydroxy	C12H15NO4	237.10011	[M+H]+	238.10739	3.60
60	Carbophenothion	C11H16ClO2PS3	341.97386	[M+H]+	342.98114	13.50
61	Carboxin	C12H13NO2S	235.06670	[M+H]+	236.07398	5.47
62	Carfentrazone-ethyl	C15H14Cl2F3N3O3	411.03643	[M+H]+	412.04371	9.98
63	Carpropamide	C15H18Cl3NO	333.04540	[M+H]+	334.05268	10.28
64	Chlorantraniliprole	C18H14BrCl2N5O2	480.97079	[M+H]+	481.97807	6.97
65	Chlorbufam	C11H10ClNO2	223.04001	[M+H]+	224.04728	7.73
66	Chlorfenapyr	C15H11BrClF3N2O	405.96954	[M+NH4]+	424.00336	11.78
67	Chlorfenvinphos	C12H14Cl3O4P	357.96953	[M+H]+	358.97681	10.44
68	Chlorfluazuron	C20H9Cl3F5N3O3	538.96297	[M+H]+	539.97024	13.84
69	Chlorflurenol-methyl	C15H11ClO3	274.03967	[M+NH4]+	292.07350	7.06
70	Chloridazon	C10H8ClN3O	221.03559	[M+H]+	222.04287	3.76
71	Chlorimuron-ethyl	C15H15ClN4O6S	414.04009	[M+H]+	415.04736	8.03
72	Chlorobenzuron	C14H10Cl2N2O2	308.01193	[M+H]+	309.01921	10.09
73	Chlorotoluron	C10H13ClN2O	212.07164	[M+H]+	213.07892	6.06
74	Chloroxuron	C15H15ClN2O2	290.08221	[M+H]+	291.08948	8.69
75	Chlorpyrifos	C9H11Cl3NO3PS	348.92629	[M+H]+	349.93357	13.12
76	Chlorpyrifos-methyl	C7H7Cl3NO3PS	320.89499	[M+H]+	321.90227	11.43
77	Chlorthiophos	C11H15Cl2O3PS2	359.95773	[M+H]+	360.96501	13.48
78	Chromafenozide	C24H30N2O3	394.22564	[M+H]+	395.23292	8.80
79	Cinmethylin	C18H26O2	274.19328	[M+NH4]+	292.22711	12.64
80	Clethodim	C17H26ClNO3S	359.13219	[M+H]+	360.13947	11.88
81	Clethodim sulfone	C17H26ClNO5S	391.12202	[M+H]+	392.12930	6.78
82	Clethodim sulfoxide	C17H26ClNO4S	375.12711	[M+H]+	376.13439	6.92
83	Clofentezine	C14H8Cl2N4	302.01260	[M+H]+	303.01988	10.99
84	Clomazone	C12H14ClNO2	239.07131	[M+H]+	240.07858	7.25
85	Clomeprop	C16H15Cl2NO2	323.04799	[M+H]+	324.05526	12.34
86	Coumaphos	C14H16ClO5PS	362.01446	[M+H]+	363.02174	10.33
87	Crufomate	C12H19ClNO3P	291.07911	[M+H]+	292.08639	9.61
88	Cyanazine	C9H13ClN6	240.08902	[M+H]+	241.09630	4.61
89	Cyanophos	C9H10NO3PS	243.01190	[M+H]+	244.01918	6.29
90	Cyantraniliprole	C19H14BrClN6O2	472.00501	[M-H]-	470.99774	5.52
91	Cyazofamid	C13H13ClN4O2S	324.04478	[M+H]+	325.05205	9.22
92	Cyclaniliprole	C21H17Br2Cl2N5O2	598.91260	[M-H]-	597.90533	9.52
93	Cycloate	C11H21NOS	215.13439	[M+H]+	216.14166	11.44
94	Cycloprothrin	C26H21Cl2NO4	481.08477	[M+NH4]+	499.11859	13.66
95	Cyclosulfamuron	C17H19N5O6S	421.10561	[M+H]+	422.11288	8.66
96	Cyenopyrafen	C24H31N3O2	393.24163	[M+H]+	394.24890	13.63
97	Cyflufenamid	C20H17F5N2O2	412.12102	[M+H]+	413.12830	10.82

No.	Compound name	Molecular formula formula	Monoisotopic mass	Ionization type	Theoretical m/z	t_R
98	Cyflumetofen	C24H24F3NO4	447.16574	[M+NH4]+	465.19957	11.86
99	Cyhalofop-butyl	C20H20FNO4	357.13764	[M+NH4]+	375.17146	11.45
100	Cyhalothrin	C23H19ClF3NO3	449.10056	[M+NH4]+	467.13438	13.68
101	Cymoxanil	C7H10N4O3	198.07529	[M+H]+	199.08257	3.92
102	Cyprazine	C9H14ClN5	227.09377	[M+H]+	228.10105	6.45
103	Cyproconazole	C15H18ClN3O	291.11384	[M+H]+	292.12112	8.82
104	Cyprodinil	C14H15N3	225.12660	[M+H]+	226.13387	9.83
105	Cyromazine	C6H10N6	166.09669	[M+H]+	167.10397	2.74
106	Daimuron (Dymron)	C17H20N2O	268.15756	[M+H]+	269.16484	8.40
107	Deltamethrin	C22H19Br2NO3	502.97317	[M+NH4]+	521.00699	14.07
108	Demeton-S	C8H19O3PS2	258.05133	[M+H]+	259.05860	7.30
109	Demeton-S sulfone	C8H19O5PS2	290.04116	[M+H]+	291.04843	3.78
110	Demeton-S sulfoxide	C8H19O4PS2	274.04624	[M+H]+	275.05352	3.70
111	Demeton-S-methyl	C6H15O3PS2	230.02003	[M+H]+	231.02730	5.23
112	Demeton-S-methylsulfone	C6H15O5PS2	262.00986	[M+H]+	263.01713	3.16
113	Desmetryn	C8H15N5S	213.10482	[M+H]+	214.11209	5.62
114	Dialifor	C14H17ClNO4PS2	393.00252	[M+H]+	394.00980	11.10
115	Di-allate	C10H17Cl2NOS	269.04079	[M+H]+	270.04807	11.84
116	Diazinon	C12H21N2O3PS	304.10105	[M+H]+	305.10833	10.48
117	Dichlobenil	C7H3Cl2N	170.96426	[M+NH4]+	188.99808	11.11
118	Dichlofenthion	C10H13Cl2O3PS	313.97001	[M+H]+	314.97729	12.92
119	Dichlormid	C8H11Cl2NO	207.02177	[M+H]+	208.02905	5.40
120	Dichlorvos	C4H7Cl2O4P	219.94590	[M+H]+	220.95318	5.02
121	Diclobutrazol	C15H19Cl2N3O	327.09052	[M+H]+	328.09780	9.99
122	Diclocymet	C15H18Cl2N2O	312.07962	[M-H]-	311.07234	9.62
123	Diclofop-methyl	C16H14Cl2O4	340.02692	[M+NH4]+	358.06074	12.46
124	Diclosulam	C13H10Cl2FN5O3S	404.98655	[M+H]+	405.99382	5.59
125	Dicrotophos	C8H16NO5P	237.07661	[M+H]+	238.08389	3.27
126	Dicyclanil	C8H10N6	190.09669	[M+H]+	191.10397	1.72
127	Diethatyl-ethyl	C16H22ClNO3	311.12882	[M+H]+	312.13610	9.82
128	Diethofencarb	C14H21NO4	267.14706	[M+H]+	268.15434	7.53
129	Difenoconazole	C19H17Cl2N3O3	405.06470	[M+H]+	406.07198	11.20
130	Diflubenzuron	C14H9ClF2N2O2	310.03206	[M+H]+	311.03934	9.60
131	Diflufenican	C19H11F5N2O2	394.07407	[M+H]+	395.08135	11.58
132	Dimepiperate	C15H21NOS	263.13439	[M+H]+	264.14166	11.58
133	Dimethametryn	C11H21N5S	255.15177	[M+H]+	256.15904	9.36
134	Dimethenamid	C12H18ClNO2S	275.07468	[M+NH4]+	293.10851	7.90
135	Dimethoate	C5H12NO3PS2	228.99963	[M+H]+	230.00690	3.70
136	Dimethylvinphos	C10H10Cl3O4P	329.93823	[M+H]+	330.94551	9.22
137	Dinitramine	C11H13F3N4O4	322.08889	[M+H]+	323.09617	10.82
138	Dioxathion	C12H26O6P2S4	456.00875	[M+NH4]+	474.04258	12.39
139	Diphenamid	C16H17NO	239.13101	[M+H]+	240.13829	6.87
140	Disulfoton sulfone	C8H19O4PS3	306.01831	[M+H]+	307.02559	6.45
141	Disulfoton sulfoxide	C8H19O3PS3	290.02340	[M+H]+	291.03068	5.94
142	Dithiopyr	C15H16F5NO2S2	401.05426	[M+H]+	402.06154	11.70
143	Diuron	C9H10Cl2N2O	232.01702	[M+H]+	233.02430	6.67
144	DNOC	C7H6N2O5	198.02767	[M-H]-	197.02040	6.22
145	Dodine	C15H33N3O2	287.25728	[M+H-C2H4O2]+	228.24342	9.83
146	Edifenphos	C14H15O2PS2	310.02511	[M+H]+	311.03239	10.11
147	Emamectin B1a	C49H75NO13	885.52384	[M+H]+	886.53112	11.89
148	Epoxiconazole	C17H13ClFN3O	329.07312	[M+H]+	330.08040	9.19

No.	Compound name	Molecular formula formula	Monoisotopic mass	Ionization type	Theoretical m/z	t_R
149	EPTC	C9H19NOS	189.11874	[M+H]+	190.12601	9.94
150	Esprocarb	C15H23NOS	265.15004	[M+H]+	266.15731	12.46
151	Etaconazole	C14H15Cl2N3O2	327.05413	[M+H]+	328.06141	9.13
152	Ethaboxam	C14H16N4OS2	320.07656	[M+H]+	321.08383	5.77
153	Ethametsulfuron-methyl	C15H18N6O6S	410.10086	[M+H]+	411.10813	5.43
154	Ethiofencarb	C11H15NO2S	225.08235	[M+H]+	226.08963	5.79
155	Ethion	C9H22O4P2S4	383.98762	[M+H]+	384.99490	12.74
156	Ethofumesate	C13H18O5S	286.08750	[M+NH4]+	304.12132	7.58
157	Ethofumesate metabolte (NC 20645)	C11H14O6S	274.05111	[M-H]-	273.04384	3.76
158	Ethoprophos (Ethoprop)	C8H19O2PS2	242.05641	[M+H]+	243.06369	9.16
159	Ethychlozate	C11H11ClN2O2	238.05091	[M+H]+	239.05818	7.18
160	Etofenprox	C25H28O3	376.20385	[M+NH4]+	394.23767	15.47
161	Etrimfos	C10H17N2O4PS	292.06467	[M+H]+	293.07195	10.30
162	Famoxadone	C22H18N2O4	374.12666	[M+NH4]+	392.16048	10.46
163	Fenamidone	C17H17N3OS	311.10923	[M+H]+	312.11651	7.82
164	Fenamiphos	C13H22NO3PS	303.10580	[M+H]+	304.11308	9.56
165	Fenarimol	C17H12Cl2N2O	330.03267	[M+H]+	331.03995	9.01
166	Fenazaquin	C20H22N2O	306.17321	[M+H]+	307.18049	14.39
167	Fenbuconazole	C19H17ClN4	336.11417	[M+H]+	337.12145	9.38
168	Fenfuram	C12H11NO2	201.07898	[M+H]+	202.08626	5.63
169	Fenhexamid	C14H17Cl2NO2	301.06364	[M+H]+	302.07091	8.97
170	Fenobucarb	C12H17NO2	207.12593	[M+H]+	208.13321	7.50
171	Fenothiocarb	C13H19NO2S	253.11365	[M+H]+	254.12093	9.79
172	Fenoxanil	C15H18Cl2N2O2	328.07453	[M+H]+	329.08181	9.58
173	Fenoxaprop-ethyl	C18H16ClNO5	361.07170	[M+H]+	362.07898	12.10
174	Fenoxycarb	C17H19NO4	301.13141	[M+H]+	302.13869	9.73
175	Fenpropathrin	C22H23NO3	349.16779	[M+H]+	350.17507	13.59
176	Fenpropimorph	C20H33NO	303.25621	[M+H]+	304.26349	6.79
177	Fenpyrazamine	C17H21N3O2S	331.13545	[M+H]+	332.14273	8.52
178	Fenpyroximate	C24H27N3O4	421.20016	[M+H]+	422.20743	13.70
179	Fensulfothion	C11H17O4PS2	308.03059	[M+H]+	309.03787	6.36
180	Fenthion	C10H15O3PS2	278.02003	[M+H]+	279.02730	10.33
181	Fentrazamide	C16H20ClN5O2	349.13055	[M+H]+	350.13783	10.16
182	Ferimzone	C15H18N4	254.15315	[M+H]+	255.16042	7.22
183	Fipronil	C12H4Cl2F6N4OS	435.93871	[M+H]+	436.94598	9.65
184	Flamprop-isopropyl	C19H19ClFNO3	363.10375	[M+H]+	364.11103	10.31
185	Flazasulfuron	C13H12F3N5O5S	407.05113	[M+H]+	408.05840	6.88
186	Flonicamid	C9H6F3N3O	229.04630	[M+H]+	230.05357	3.22
187	Florpyrauxifen	C13H8Cl2F2N2O3	347.98801	[M+H]+	348.99528	4.95
188	Fluacrypyrim	C20H21F3N2O5	426.14026	[M+H]+	427.14753	11.27
189	Fluazifop Butyl	C19H20F3NO4	383.13444	[M+H]+	384.14172	12.18
190	Fluazinam	C13H4Cl2F6N4O4	463.95138	[M-H]-	462.94410	12.65
191	Flubendiamide	C23H22F7IN2O4S	682.02332	[M+H]+	683.03060	9.86
192	Flucetosulfuron	C18H22FN5O8S	487.11732	[M+H]+	488.12459	7.35
193	Flucythrinate	C26H23F2NO4	451.15952	[M+NH4]+	469.19334	13.19
194	Fludioxonil	C12H6F2N2O2	248.03973	[M-H]-	247.03246	7.88
195	Flufenacet	C14H13F4N3O2S	363.06646	[M+H]+	364.07374	9.12
196	Flufenoxuron	C21H11ClF6N2O3	488.03624	[M+H]+	489.04352	13.32
197	Flufenpyr-ethyl	C16H13ClF4N2O4	408.05000	[M+H]+	409.05728	9.65
198	Flumetralin	C16H12ClF4N3O4	421.04525	[M+H]+	422.05252	13.50
199	Flumioxazin	C19H15FN2O4	354.10159	[M+H]+	355.10886	6.95

No.	Compound name	Molecular formula formula	Monoisotopic mass	Ionization type	Theoretical m/z	t_R
200	Fluometuron	C10H11F3N2O	232.08235	[M+H]+	233.08962	5.89
201	Fluopicolide	C14H8Cl3F3N2O	381.96543	[M+H]+	382.97271	8.30
202	Fluopyram	C16H11ClF6N2O	396.04641	[M+H]+	397.05369	8.82
203	Flupoxam	C19H14ClF5N4O2	460.07254	[M+H]+	461.07982	9.75
204	Flupyradifurone	C12H11ClF2N2O2	288.04771	[M+H]+	289.05499	3.59
205	Fluquinconazole	C16H8Cl2FN5O	375.00899	[M+H]+	376.01627	8.87
206	Fluridone	C19H14F3NO	329.10275	[M+H]+	330.11003	7.09
207	Flurochloridone	C12H10Cl2F3NO	311.00915	[M+H]+	312.01643	8.63
208	Flurtamone	C18H14F3NO2	333.09766	[M+H]+	334.10494	7.56
209	Flusilazole	C16H15F2N3Si	315.10033	[M+H]+	316.10761	9.65
210	Flusulfamide	C13H7Cl2F3N2O4S	413.94557	[M-H]-	412.93829	10.76
211	Fluthiacet-methyl	C15H15ClFN3O3S2	403.02274	[M+H]+	404.03002	9.94
212	Flutianil	C19H14F4N2OS2	426.04837	[M+H]+	427.05565	9.96
213	Flutolanil	C17H16F3NO2	323.11331	[M+H]+	324.12059	8.17
214	Flutriafol	C16H13F2N3O	301.10267	[M+H]+	302.10995	6.22
215	Fluvalinate	C26H22ClF3N2O3	502.12711	[M+H]+	503.13438	14.56
216	Fluxametamide	C20H16Cl2F3N3O3	473.05208	[M+H]+	474.05936	12.90
217	Fluxapyroxad	C18H12F5N3O	381.09005	[M+H]+	382.09733	8.24
218	Fomesafen	C15H10ClF3N2O6S	437.99002	[M-H]-	436.98275	8.03
219	Fonofos	C10H15OPS2	246.03020	[M+H]+	247.03747	10.51
220	Foramsulfuron	C17H20N6O7S	452.11142	[M+H]+	453.11870	5.29
221	Forchlorfenuron	C12H10ClN3O	247.05124	[M-H]-	246.04396	6.55
222	Fosthiazate	C9H18NO3PS2	283.04658	[M+H]+	284.05385	5.82
223	Furathiocarb	C18H26N2O5S	382.15625	[M+H]+	383.16352	12.21
224	GPTC	C23H29NO8S	479.16139	[M+H]+	480.16867	3.96
225	Halfenprox	C24H23BrF2O3	476.07986	[M+NH4]+	494.11369	15.98
226	Halosulfuron-methyl	C13H15ClN6O7S	434.04115	[M+H]+	435.04843	8.80
227	Heptenophos	C9H12ClO4P	250.01618	[M+H]+	251.02345	6.66
228	Hexaconazole	C14H17Cl2N3O	313.07487	[M+H]+	314.08215	10.66
229	Hexaflumuron	C16H8Cl2F6N2O3	459.98162	[M-H]-	458.97434	11.55
230	Hexazinone	C12H20N4O2	252.15863	[M+H]+	253.16590	5.15
231	Hexythiazox	C17H21ClN2O2S	352.10123	[M+H]+	353.10851	13.04
232	Imazalil	C14H14Cl2N2O	296.04832	[M+H]+	297.05560	5.30
233	Imazamethabenz-methyl	C16H20N2O3	288.14739	[M+H]+	289.15467	5.04
234	Imazamox	C15H19N3O4	305.13756	[M+H]+	306.14483	3.81
235	Imazamox metabolite (M720H001)	C14H17N3O4	291.12191	[M+H]+	292.12918	3.35
236	Imazapic	C14H17N3O3	275.12699	[M+H]+	276.13427	3.92
237	Imazaquin	C17H17N3O3	311.12699	[M+H]+	312.13427	4.97
238	Imazethapyr	C15H19N3O3	289.14264	[M+H]+	290.14992	4.51
239	Imazosulfuron	C14H13ClN6O5S	412.03567	[M+H]+	413.04295	7.95
240	Imibenconazole	C17H13Cl3N4S	409.99265	[M+H]+	410.99993	12.57
241	Imicyafos	C11H21N4O2PS	304.11229	[M+H]+	305.11956	4.47
242	Imidacloprid	C9H10ClN5O2	255.05230	[M+H]+	256.05958	3.40
243	Inabenfide	C19H15ClN2O2	338.08221	[M-H]-	337.07493	7.68
244	Indanofan	C20H17ClO3	340.08662	[M+H]+	341.09390	9.24
245	Indaziflam	C16H20FN5	301.17027	[M+H]+	302.17755	9.15
246	Indoxacarb	C22H17ClF3N3O7	527.07071	[M+H]+	528.07799	11.38
247	Ipconazole	C18H24ClN3O	333.16079	[M+H]+	334.16807	11.66
248	Ipfencarbazone	C18H14Cl2F2N4O2	426.04619	[M+H]+	427.05347	10.21
249	Iprobenfos	C13H21O3PS	288.09491	[M+H]+	289.10218	9.87
250	Iprovalicarb	C18H28N2O3	320.20999	[M+H]+	321.21727	8.92

No.	Compound name	Molecular formula formula	Monoisotopic mass	Ionization type	Theoretical m/z	t_R
251	Isazofos	C9H17ClN3O3PS	313.04168	[M+H]+	314.04896	8.71
252	Isofenphos	C15H24NO4PS	345.11637	[M+H]+	346.12365	10.93
253	Isofenphos-methyl	C14H22NO4PS	331.10072	[M+H]+	332.10800	10.05
254	Isoprocarb	C11H15NO2	193.11028	[M+H]+	194.11756	6.29
255	Isopropalin	C15H23N3O4	309.16886	[M+H]+	310.17613	14.08
256	Isoprothiolane	C12H18O4S2	290.06465	[M+H]+	291.07193	8.20
257	Isoproturon	C12H18N2O	206.14191	[M+H]+	207.14919	6.50
258	Isopyrazam	C20H23F2N3O	359.18092	[M+H]+	360.18820	11.25
259	Isotianil	C11H5Cl2N3OS	296.95304	[M-H]-	295.94576	8.06
260	Isouron	C10H17N3O2	211.13208	[M+H]+	212.13935	5.36
261	Isoxaben	C18H24N2O4	332.17361	[M-H]-	331.16633	8.07
262	Isoxadifen	C16H13NO3	267.08954	[M+H]+	268.09682	6.33
263	Isoxadifen-ethyl	C18H17NO3	295.12084	[M+H]+	296.12812	9.92
264	Isoxathion	C13H16NO4PS	313.05377	[M+H]+	314.06105	10.76
265	Ivermectin B1a	C48H74O14	874.50786	[M+NH4]+	892.54169	15.93
266	Kresoxim-methyl	C18H19NO4	313.13141	[M+H]+	314.13869	10.06
267	Lancotrione	C19H20ClNaO8S	466.04652	[M+H]+	467.05379	5.43
268	Lenacil	C13H18N2O2	234.13683	[M+H]+	235.14411	6.39
269	Leptophos	C13H10BrCl2O2PS	409.86996	[M+H]+	410.87723	14.85
270	Linuron	C9H10Cl2N2O2	248.01193	[M+H]+	249.01921	7.65
271	Lufenuron	C17H8Cl2F8N2O3	509.97842	[M-H]-	508.97115	12.58
272	malaoxon	C10H19O7PS	314.05892	[M+H]+	315.06619	5.03
273	Malathion	C10H19O6PS2	330.03607	[M+H]+	331.04335	8.24
274	Mandestrobin	C19H23NO3	313.16779	[M+H]+	314.17507	10.29
275	Mandipropamid	C23H22ClNO4	411.12374	[M+H]+	412.13101	7.96
276	Mecarbam	C10H20NO5PS2	329.05206	[M+H]+	330.05933	9.07
277	Mefenacet	C16H14N2O2S	298.07760	[M+H]+	299.08488	8.57
278	Mefenpyr-diethyl	C16H18Cl2N2O4	372.06436	[M+H]+	373.07164	10.62
279	Mefentrifluconazole	C18H15ClF3N3O2	397.08049	[M+H]+	398.08777	10.49
280	Mepanipyrim	C14H13N3	223.11095	[M+H]+	224.11822	8.93
281	Mephosfolan	C8H16NO3PS2	269.03093	[M+H]+	270.03820	4.81
282	Mepronil	C17H19NO2	269.14158	[M+H]+	270.14886	8.31
283	Mesosulfuron-methyl	C17H21N5O9S2	503.07807	[M+H]+	504.08535	5.91
284	Mesotrione	C14H13NO7S	339.04128	[M+H]+	340.04855	3.86
285	Metaflumizone	C24H16F6N4O2	506.11774	[M+H]+	507.12502	11.43
286	Metamifop	C23H18ClFN2O4	440.09391	[M+H]+	441.10119	12.08
287	Metamitron	C10H10N4O	202.08546	[M+H]+	203.09274	3.77
288	Metazosulfuron	C15H18ClN7O7S	475.06770	[M+H]+	476.07498	7.56
289	Metconazole	C17H22ClN3O	319.14514	[M+H]+	320.15242	10.74
290	Methabenzthiazuron	C10H11N3OS	221.06228	[M+H]+	222.06956	6.33
291	Methacrifos	C7H13O5PS	240.02214	[M+H]+	241.02941	6.98
292	Methidathion	C6H11N2O4PS3	301.96186	[M+H]+	302.96914	6.91
293	Methiocarb	C11H15NO2S	225.08235	[M+H]+	226.08963	7.78
294	Methoprotryn	C11H21N5OS	271.14668	[M+H]+	272.15396	6.91
295	Methoxyfenozide	C22H28N2O3	368.20999	[M+H]+	369.21727	8.26
296	Metolachlor	C15H22ClNO2	283.13391	[M+H]+	284.14118	9.40
297	Metolcarb	C9H11NO2	165.07898	[M+H]+	166.08626	4.69
298	Metominostrobin	C16H16N2O3	284.11609	[M+H]+	285.12337	6.75
299	Metosulam	C14H13Cl2N5O4S	417.00653	[M+H]+	418.01381	4.99
300	Metrafenone	C19H21BrO5	408.05724	[M+H]+	409.06451	11.02
301	Metribuzin	C8H14N4OS	214.08883	[M+H]+	215.09611	5.23

No.	Compound name	Molecular formula formula	Monoisotopic mass	Ionization type	Theoretical m/z	t_R
302	Mevinphos	C7H13O6P	224.04498	[M+H]+	225.05226	3.97
303	MGK-264	C17H25NO2	275.18853	[M+H]+	276.19581	11.32
304	Milbemectin A4	C32H46O7	542.32436	[M+H-H2O]+	525.32107	15.53
305	Molinate	C9H17NOS	187.10309	[M+H]+	188.11036	8.65
306	Monocrotophos	C7H14NO5P	223.06096	[M+H]+	224.06824	3.21
307	Monolinuron	C9H11ClN2O2	214.05091	[M+H]+	215.05818	5.80
308	Myclobutanil	C15H17ClN4	288.11417	[M+H]+	289.12145	8.44
309	Naftalofos	C16H16NO6P	349.07153	[M+H]+	350.07880	7.95
310	Napropamide	C17H21NO2	271.15723	[M+H]+	272.16451	9.20
311	Neburon	C12H16Cl2N2O	274.06397	[M+H]+	275.07125	9.83
312	Nicosulfuron	C15H18N6O6S	410.10086	[M+H]+	411.10813	4.73
313	Nitenpyram	C11H15ClN4O2	270.08835	[M+H]+	271.09563	3.07
314	Norea (Noruron)	C13H22N2O	222.17321	[M+H]+	223.18049	7.80
315	Norflurazon	C12H9ClF3N3O	303.03862	[M+H]+	304.04590	6.64
316	Novaluron	C17H9ClF8N2O4	492.01231	[M+H]+	493.01959	11.70
317	Nuarimol	C17H12ClFN2O	314.06222	[M+H]+	315.06950	7.61
318	Ofurace	C14H16ClNO3	281.08187	[M+H]+	282.08915	5.03
319	Omethoate	C5H12NO4PS	213.02247	[M+H]+	214.02975	2.97
320	Orthosulfamuron	C16H20N6O6S	424.11651	[M+H]+	425.12378	6.28
321	Orysastrobin	C18H25N5O5	391.18557	[M+H]+	392.19285	8.22
322	Orysastrobin metabolite (F001)	C18H25N5O5	391.18557	[M+H]+	392.19285	8.76
323	Oryzalin	C12H18N4O6S	346.09471	[M-H]-	345.08743	9.18
324	Oxadiargyl	C15H14Cl2N2O3	340.03815	[M+H]+	341.04543	10.71
325	Oxadiazon	C15H18Cl2N2O3	344.06945	[M+NH4]+	362.10328	12.63
326	Oxadixyl	C14H18N2O4	278.12666	[M+H]+	279.13394	4.36
327	Oxathiapiprolin	C24H22F5N5O2S	539.14144	[M+H]+	540.14871	8.10
328	Oxaziclomefone	C20H19Cl2NO2	375.07929	[M+H]+	376.08656	12.05
329	Oxycarboxin	C12H13NO4S	267.05653	[M+H]+	268.06381	3.89
330	Oxydemeton-methyl	C6H15O4PS2	246.01494	[M+H]+	247.02222	3.11
331	Oxyfluorfen	C15H11ClF3NO4	361.03287	[M+H]+	362.04015	12.25
332	Paclobutrazol	C15H20ClN3O	293.12949	[M+H]+	294.13677	8.19
333	Parathion	C10H14NO5PS	291.03303	[M+H]+	292.04031	9.91
334	Parathion-Methyl	C8H10NO5PS	263.00173	[M+H]+	264.00901	3.16
335	Pebulate	C10H21NOS	203.13439	[M+H]+	204.14166	11.25
336	Penconazole	C13H15Cl2N3	283.06430	[M+H]+	284.07158	10.09
337	Pencycuron	C19H21ClN2O	328.13424	[M+H]+	329.14152	11.08
338	Pendimethalin	C13H19N3O4	281.13756	[M+H]+	282.14483	13.22
339	Penflufen	C18H24FN3O	317.19034	[M+H]+	318.19762	9.94
340	Penoxsulam	C16H14F5N5O5S	483.06358	[M+H]+	484.07086	5.38
341	Penthiopyrad	C16H20F3N3OS	359.12792	[M+H]+	360.13520	10.06
342	Pentoxazone	C17H17ClFNO4	353.08302	[M+H]+	354.09029	12.15
343	Permethrin	C21H20Cl2O3	390.07895	[M+NH4]+	408.11278	15.28
344	Phenothrin	C23H26O3	350.18820	[M+H]+	351.19547	15.22
345	Phenthoate	C12H17O4PS2	320.03059	[M+H]+	321.03787	9.90
346	Phorate oxon	C7H17O3PS2	244.03568	[M+H]+	245.04295	6.92
347	Phorate oxon sulfone	C7H17O5PS2	276.02551	[M+H]+	277.03278	3.79
348	Phorate oxon sulfoxide	C7H17O4PS2	260.03059	[M+H]+	261.03787	3.60
349	Phorate sulfone	C7H17O4PS3	292.00266	[M+H]+	293.00994	6.16
350	Phorate sulfoxide	C7H17O3PS3	276.00775	[M+H]+	277.01503	5.95
351	Phosalone	C12H15ClNO4PS2	366.98687	[M+H]+	367.99415	10.89
352	Phosfolan	C7H14NO3PS2	255.01528	[M+H]+	256.02255	4.15

No.	Compound name	Molecular formula formula	Monoisotopic mass	Ionization type	Theoretical m/z	t_R
353	Phosmet	C11H12NO4PS2	316.99454	[M+H]+	318.00182	7.13
354	Phosphamidon	C10H19ClNO5P	299.06894	[M+H]+	300.07622	4.34
355	Phoxim	C12H15N2O3PS	298.05410	[M+H]+	299.06138	10.70
356	Picarbutrazox	C20H23N7O3	409.18624	[M+H]+	410.19352	9.49
357	Picolinafen	C19H12F4N2O2	376.08349	[M+H]+	377.09077	12.42
358	Picoxystrobin	C18H16F3NO4	367.10314	[M+H]+	368.11042	9.67
359	Pinoxaden	C23H32N2O4	400.23621	[M+H]+	401.24349	10.74
360	Pinoxaden metabolite (SYN 505164)	C18H24N2O4	332.17361	[M+H]+	333.18089	3.58
361	Piperonyl butoxide	C19H30O5	338.20933	[M+NH4]+	356.24315	12.68
362	Piperophos	C14H28NO3PS2	353.12483	[M+H]+	354.13210	11.38
363	Pirimicarb	C11H18N4O2	238.14298	[M+H]+	239.15025	5.10
364	Pirimiphos-ethyl	C13H24N3O3PS	333.12760	[M+H]+	334.13488	12.55
365	Pirimiphos-methyl	C11H20N3O3PS	305.09630	[M+H]+	306.10358	10.90
366	Pretilachlor	C17H26ClNO2	311.16521	[M+H]+	312.17248	11.70
367	Probenazole	C10H9NO3S	223.03032	[M+H]+	224.03759	4.58
368	Prochloraz metabolite (BTS 44595)	C12H15Cl3N2O2	324.01991	[M+H]+	325.02719	10.66
369	Procymidone	C13H11Cl2NO2	283.01669	[M+H]+	284.02396	9.16
370	Prodiamine	C13H17F3N4O4	350.12019	[M+H]+	351.12747	12.61
371	Profenofos	C11H15BrClO3PS	371.93514	[M+H]+	372.94242	12.06
372	Prohydrojasmon	C15H26O3	254.18820	[M+H]+	255.19547	11.16
373	Promecarb	C12H17NO2	207.12593	[M+H]+	208.13321	8.10
374	Prometryn	C10H19N5S	241.13612	[M+H]+	242.14339	8.26
375	Propachlor	C11H14ClNO	211.07639	[M+H]+	212.08367	6.47
376	Propamocarb	C9H20N2O2	188.15248	[M+H]+	189.15976	2.92
377	Propanil	C9H9Cl2NO	217.00612	[M+H]+	218.01340	7.76
378	Propargite	C19H26O4S	350.15518	[M+NH4]+	368.18901	13.40
379	Propazine	C9H16ClN5	229.10942	[M+H]+	230.11670	7.69
380	Propetamphos	C10H20NO4PS	281.08507	[M+H]+	282.09235	8.55
381	Propiconazole	C15H17Cl2N3O2	341.06978	[M+H]+	342.07706	10.47
382	Propisochlor	C15H22ClNO2	283.13391	[M+H]+	284.14118	10.29
383	Propoxur	C11H15NO3	209.10519	[M+H]+	210.11247	5.06
384	Propyrisulfuron	C16H18ClN7O5S	455.07787	[M+H]+	456.08515	8.23
385	Proquinazid	C14H17IN2O2	372.03347	[M+H]+	373.04075	14.13
386	Prosulfocarb	C14H21NOS	251.13439	[M+H]+	252.14166	11.94
387	Prothiofos	C11H15Cl2O2PS2	343.96282	[M+H]+	344.97010	14.71
388	Pydiflumetofen	C16H16Cl3F2N3O2	425.02762	[M+H]+	426.03489	11.16
389	Pyflubumide	C25H31F6N3O3	535.22696	[M+H]+	536.23424	12.74
390	Pyflubumide-NH	C21H25F6N3O2	465.18510	[M+H]+	466.19237	12.00
391	Pyracarbolid	C13H15NO2	217.11028	[M+H]+	218.11756	5.31
392	Pyraclofos	C14H18ClN2O3PS	360.04643	[M+H]+	361.05371	10.80
393	Pyraclonil	C15H15ClN6	314.10467	[M+H]+	315.11195	5.75
394	Pyraclostrobin	C19H18ClN3O4	387.09859	[M+H]+	388.10586	10.66
395	Pyraflufen-ethyl	C15H13Cl2F3N2O4	412.02045	[M+H]+	413.02773	10.28
396	Pyraziflumid	C18H10F5N3O	379.07440	[M+H]+	380.08168	8.91
397	Pyrazosulfuron-ethyl	C14H18N6O7S	414.09577	[M+H]+	415.10305	8.76
398	Pyrazoxyfen	C20H16Cl2N2O3	402.05380	[M+H]+	403.06108	10.26
399	Pyribencarb E	C18H20ClN3O3	361.11932	[M+H]+	362.12660	8.03
400	Pyribencarb Z (KIE-9749)	C18H20ClN3O3	361.11932	[M+H]+	362.12660	7.14
401	Pyribenzoxim	C32H27N5O8	609.18597	[M+H]+	610.19324	12.29
402	Pyributicarb	C18H22N2O2S	330.14020	[M+H]+	331.14748	12.88
403	Pyridaben	C19H25ClN2OS	364.13761	[M+H]+	365.14489	14.30

No.	Compound name	Molecular formula formula	Monoisotopic mass	Ionization type	Theoretical m/z	t_R
404	Pyridalyl	C18H14Cl4F3NO3	488.96799	[M+H]+	489.97527	16.19
405	Pyridaphenthion	C14H17N2O4PS	340.06467	[M+H]+	341.07195	8.48
406	Pyrifenox	C14H12Cl2N2O	294.03267	[M+H]+	295.03995	8.91
407	Pyrifluquinazon	C19H15F7N4O2	464.10832	[M+H]+	465.11560	8.56
408	Pyriftalid	C15H14N2O4S	318.06743	[M+H]+	319.07471	7.18
409	Pyrimethanil	C12H13N3	199.11095	[M+H]+	200.11822	7.55
410	Pyrimethanil-5-hydroxy	C12H13N3O	215.10586	[M+H]+	216.11314	5.79
411	Pyrimidifen	C20H28ClN3O2	377.18701	[M+H]+	378.19428	12.38
412	Pyriminobac-methyl	C17H19N3O6	361.12739	[M+H]+	362.13466	7.24
413	Pyrimisulfan	C16H19F2N3O6S	419.09627	[M+H]+	420.10354	6.71
414	Pyriofenone	C18H20ClNO5	365.10300	[M+H]+	366.11028	11.17
415	Pyriproxyfen	C20H19NO3	321.13649	[M+H]+	322.14377	12.93
416	Pyroquilon	C11H11NO	173.08406	[M+H]+	174.09134	4.95
417	Quinalphos	C12H15N2O3PS	298.05410	[M+H]+	299.06138	9.97
418	Quinclorac methyl ester	C11H7Cl2NO2	254.98539	[M+H]+	255.99266	7.47
419	Quinoclamine	C10H6ClNO2	207.00871	[M+H]+	208.01598	4.77
420	Quinoxyfen	C15H8Cl2FNO	306.99670	[M+H]+	308.00398	13.02
421	Quizalofop-ethyl	C19H17ClN2O4	372.08769	[M+H]+	373.09496	12.16
422	Resmethrin	C22H26O3	338.18820	[M+H]+	339.19547	14.72
423	Rimsulfuron	C14H17N5O7S2	431.05694	[M+H]+	432.06422	5.33
424	Saflufenacil	C17H17ClF4N4O5S	500.05443	[M-H]-	499.04716	7.22
425	Sedaxane	C18H19F2N3O	331.14962	[M+H]+	332.15690	8.60
426	Sethoxydim	C17H29NO3S	327.18682	[M+H]+	328.19409	12.43
427	Simazine	C7H12ClN5	201.07812	[M+H]+	202.08540	5.16
428	Simeconazole	C14H20FN3OSi	293.13597	[M+H]+	294.14324	9.09
429	Simetryn	C8H15N5S	213.10482	[M+H]+	214.11209	5.56
430	Spinetoram-J	C42H69NO10	747.49215	[M+H]+	748.49943	10.78
431	Spinetoram-L	C43H69NO10	759.49215	[M+H]+	760.49943	11.50
432	Spinosyn A	C41H65NO10	731.46085	[M+H]+	732.46813	10.70
433	Spinosyn D	C42H67NO10	745.47650	[M+H]+	746.48378	11.41
434	Spirodiclofen	C21H24Cl2O4	410.10517	[M+H]+	411.11244	13.76
435	Spiromesifen	C23H30O4	370.21441	[M+H]+	371.22169	13.23
436	Spirotetramat	C21H27NO5	373.18892	[M+H]+	374.19620	8.85
437	Spiroxamine	C18H35NO2	297.26678	[M+H]+	298.27406	6.75
438	Sulfentrazone	C11H10Cl2F2N4O3S	385.98188	[M-H]-	384.97460	5.25
439	Sulfotep	C8H20O5P2S2	322.02275	[M+H]+	323.03002	10.14
440	Sulfoxaflor	C10H10F3N3OS	277.04967	[M-H]-	276.04239	3.76
441	Sulprofos	C12H19O2PS3	322.02848	[M+H]+	323.03576	13.24
442	TCMTB	C9H6N2S3	237.96931	[M+H]+	238.97659	7.66
443	Tebuconazole	C16H22ClN3O	307.14514	[M+H]+	308.15242	10.22
444	Tebufenozide	C22H28N2O2	352.21508	[M+H]+	353.22236	9.65
445	Tebufenpyrad	C18H24ClN3O	333.16079	[M+H]+	334.16807	12.36
446	Tebufloquin	C17H20FNO2	289.14781	[M+H]+	290.15508	10.12
447	Tebufloquin metabolite (M1)	C15H18FNO	247.13724	[M+H]+	248.14452	7.45
448	Tebupirimfos	C13H23N2O3PS	318.11670	[M+H]+	319.12398	12.61
449	Tebuthiuron	C9H16N4OS	228.10448	[M+H]+	229.11176	5.27
450	Teflubenzuron	C14H6Cl2F4N2O2	379.97425	[M-H]-	378.96697	12.62
451	Tefuryltrione	C20H23ClO7S	442.08531	[M+NH4]+	460.11913	6.10
452	TEPP	C8H20O7P2	290.06843	[M+H]+	291.07571	4.42
453	Tepraloxydim	C17H24ClNO4	341.13939	[M-H]-	340.13211	5.48
454	Terbacil	C9H13ClN2O2	216.06656	[M-H]-	215.05928	5.38

No.	Compound name	Molecular formula formula	Monoisotopic mass	Ionization type	Theoretical m/z	t_R
455	Terbufos	C9H21O2PS3	288.04413	[M+H]+	289.05141	12.47
456	Terbufos oxon	C9H21O3PS2	272.06698	[M+H]+	273.07425	8.93
457	Terbufos oxon sulfone	C9H21O5PS2	304.05681	[M+H]+	305.06408	4.50
458	Terbufos oxon sulfoxide	C9H21O4PS2	288.06189	[M+H]+	289.06917	4.22
459	Terbufos sulfone	C9H21O4PS3	320.03396	[M+H]+	321.04124	7.46
460	Terbufos sulfoxide	C9H21O3PS3	304.03905	[M+H]+	305.04633	7.44
461	Terbuthylazine	C9H16ClN5	229.10942	[M+H]+	230.11670	7.92
462	Terbutryn	C10H19N5S	241.13612	[M+H]+	242.14339	8.42
463	Tetrachlorvinphos	C10H9Cl4O4P	363.89926	[M+H]+	364.90654	9.80
464	Tetraconazole	C13H11Cl2F4N3O	371.02153	[M+H]+	372.02881	9.10
465	Tetramethrin	C19H25NO4	331.17836	[M+H]+	332.18564	12.27
466	Tetraniliprole	C22H16ClF3N10O2	544.10983	[M-H]-	543.10256	6.63
467	Thenylchlor	C16H18ClNO2S	323.07468	[M+H]+	324.08196	9.03
468	Thiabendazole	C10H7N3S	201.03607	[M+H]+	202.04335	3.51
469	Thiacloprid	C10H9ClN4S	252.02365	[M+H]+	253.03092	3.81
470	Thiamethoxam	C8H10ClN5O3S	291.01929	[M+H]+	292.02657	3.18
471	Thiazopyr	C16H17F5N2O2S	396.09309	[M+H]+	397.10037	10.07
472	Thidiazuron	C9H8N4OS	220.04188	[M-H]-	219.03461	5.01
473	Thifensulfuron-methyl	C12H13N5O6S2	387.03073	[M+H]+	388.03801	4.60
474	Thifluzamide	C13H6Br2F6N2O2S	525.84209	[M-H]-	524.83481	9.39
475	Thiobencarb	C12H16ClNOS	257.06411	[M+H]+	258.07139	11.09
476	Thiometon	C6H15O2PS3	245.99718	[M+H]+	247.00446	6.13
477	Tiadinil	C11H10ClN3OS	267.02331	[M-H]-	266.01604	8.67
478	Tolclofos-methyl	C9H11Cl2O3PS	299.95436	[M+H]+	300.96164	11.00
479	Tolfenpyrad	C21H22ClN3O2	383.14006	[M+H]+	384.14733	12.54
480	Tralkoxydim	C20H27NO3	329.19909	[M+H]+	330.20637	13.21
481	Triadimefon	C14H16ClN3O2	293.09311	[M+H]+	294.10038	8.44
482	Triadimenol	C14H18ClN3O2	295.10876	[M+H]+	296.11603	10.00
483	Triafamone	C14H13F3N4O5S	406.05588	[M+H]+	407.06315	5.11
484	Tri-allate	C10H16Cl3NOS	303.00182	[M+H]+	304.00910	13.31
485	Triasulfuron	C14H16ClN5O5S	401.05607	[M+H]+	402.06335	4.59
486	Triazamate	C13H22N4O3S	314.14126	[M+H]+	315.14854	8.90
487	Triazophos	C12H16N3O3PS	313.06500	[M+H]+	314.07228	8.63
488	Tribufos	C12H27OPS3	314.09617	[M+H]+	315.10345	14.30
489	Tricyclazole	C9H7N3S	189.03607	[M+H]+	190.04335	4.09
490	Trifloxystrobin	C20H19F3N2O4	408.12969	[M+H]+	409.13697	11.50
491	Trifloxysulfuron	C14H14F3N5O6S	437.06169	[M+H]+	438.06897	6.07
492	Triflumizole	C15H15ClF3N3O	345.08557	[M+H]+	346.09285	11.60
493	Triflumuron	C15H10ClF3N2O3	358.03321	[M+H]+	359.04048	10.76
494	Triflusulfuron metabolite (IN-M7222)	C5H6F3N5O	209.05244	[M+H]+	210.05972	3.42
495	Trinexapac-ethyl	C13H16O5	252.09978	[M+H]+	253.10705	6.78
496	Triticonazole	C17H20ClN3O	317.12949	[M+H]+	318.13677	9.10
497	Tritosulfuron metabolite (M635H004)	C5H5F3N4O	194.04155	[M+H]+	195.04882	3.97
498	TZ-1E	C20H23N7O3	409.18624	[M+H]+	410.19352	9.27
499	Uniconazole	C15H18ClN3O	291.11384	[M+H]+	292.12112	9.52
500	Valifenalate	C19H27ClN2O5	398.16085	[M+H]+	399.16813	8.38
501	Vamidothion	C8H18NO4PS2	287.04149	[M+H]+	288.04877	3.51
502	Vernolate	C10H21NOS	203.13439	[M+H]+	204.14166	11.25
503	XMC	C10H13NO2	179.09463	[M+H]+	180.10191	5.83
504	Zoxamide	C14H16Cl3NO2	335.02466	[M+H]+	336.03194	10.48

Table S2. Linear ranges and correlation coefficients (r^2) for 504 pesticide multiresidues in five representative crops.

No.	Compound name	Potato		Cabbage		Mandarin		Brown rice		Soybean	
		Linear range (μg/kg)	r^2	Linear range (μg/kg)	r^2	Linear range (μg/kg)	r^2	Linear range (μg/kg)	r^2	Linear range (μg/kg)	r^2
1	2,3,5-Trimethacarb	2.5-200	0.9950	2.5-200	0.9955	10-200	0.9977	2.5-200	0.9960	2.5-250	0.9957
2	3,4,5-Trimethacarb	2.5-200	0.9869	2.5-100	0.9939	5-200	0.9900	2.5-200	0.9922	2.5-100	0.9953
3	Acetamiprid	5-100	0.9867	2.5-100	0.9854	2.5-100	0.9943	2.5-100	0.9875	2.5-100	0.9902
4	Acetochlor	2.5-200	0.9916	2.5-200	0.9903	2.5-200	0.9936	2.5-200	0.9915	2.5-250	0.9902
5	Acibenzolar acid	2.5-100	0.9883	2.5-200	0.9970	2.5-200	0.9977	2.5-200	0.9942	5-250	0.9932
6	Acibenzolar-S-methyl	2.5-200	0.9939	2.5-200	0.9975	2.5-200	0.9975	2.5-200	0.9986	2.5-250	0.9956
7	Acrinathrin	2.5-200	0.9988	2.5-200	0.9995	2.5-200	0.9991	2.5-200	0.9993	2.5-250	0.9999
8	AD-67 (MON-4660)	2.5-200	0.9933	2.5-200	0.9954	2.5-200	0.9959	2.5-200	0.9969	2.5-250	0.9951
9	Alachlor	2.5-200	0.9916	2.5-200	0.9902	2.5-200	0.9958	2.5-200	0.9928	2.5-250	0.9901
10	Aldoxycarb (Aldicarb sulfone)	2.5-100	0.9916	5-100	0.9978	5-100	0.9994	5-100	0.9949	2.5-250	0.9829
11	Allethrin	2.5-200	0.9921	2.5-200	0.9952	2.5-200	0.9936	2.5-200	0.9974	2.5-250	0.9919
12	Allidochlor	2.5-100	0.9891	2.5-200	0.9965	2.5-200	0.9854	2.5-200	0.9983	2.5-250	0.9917
13	Ametoctradin	2.5-200	0.9816	2.5-200	0.9857	2.5-100	0.9934	2.5-200	0.9923	2.5-250	0.9840
14	Ametryn	2.5-200	0.9907	2.5-200	0.9905	2.5-200	0.9940	2.5-200	0.9935	2.5-250	0.9908
15	Amisulbrom	2.5-200	0.9971	2.5-200	0.9984	2.5-200	0.9988	2.5-200	0.9978	2.5-250	0.9989
16	Anilofos	2.5-200	0.9873	2.5-200	0.9907	2.5-200	0.9952	2.5-100	0.9953	2.5-250	0.9873
17	Aramite	2.5-200	0.9917	5-200	0.9913	2.5-200	0.9914	2.5-200	0.9976	2.5-100	0.9961
18	Aspon	2.5-200	0.9933	2.5-200	0.9956	2.5-200	0.9948	2.5-200	0.9965	2.5-250	0.9962
19	Asulam	2.5-200	0.9889	10-200	0.9968	10-200	0.9993	5-200	0.9992	2.5-250	0.9987
20	Atrazine	2.5-100	0.9872	5-100	0.9886	2.5-200	0.9812	5-100	0.9892	2.5-100	0.9912
21	Avermectin B1a	2.5-200	0.9980	2.5-200	0.9974	5-200	0.9984	2.5-200	0.9958	10-250	0.9990
22	Azaconazole	2.5-100	0.9945	2.5-200	0.9905	2.5-200	0.9912	2.5-200	0.9916	2.5-200	0.9875
23	Azamethiphos	2.5-200	0.9905	2.5-200	0.9937	2.5-100	0.9979	2.5-200	0.9946	2.5-250	0.9922
24	Azimsulfuron	2.5-200	0.9979	2.5-200	0.9981	2.5-200	0.9992	2.5-200	0.9977	2.5-250	0.9979
25	Azinphos-ethyl	2.5-100	0.9952	2.5-100	0.9934	2.5-100	0.9957	5-100	0.9936	2.5-100	0.9951
26	Azoxystrobin	2.5-200	0.9900	2.5-200	0.9914	2.5-200	0.9962	2.5-100	0.9967	2.5-100	0.9966
27	Bendiocarb	2.5-100	0.9883	20-100	0.9895	2.5-100	0.9938	2.5-100	0.9907	2.5-100	0.9923
28	Benfuresate	2.5-100	0.9919	2.5-100	0.9917	2.5-100	0.9934	2.5-100	0.9927	2.5-200	0.9838
29	Benodanil	5-100	0.9918	2.5-100	0.9886	2.5-100	0.9935	5-100	0.9915	2.5-100	0.9920
30	Bensulide	2.5-200	0.9949	2.5-200	0.9955	2.5-200	0.9964	2.5-100	0.9963	5-100	0.9978
31	Benthiavalicarb-isopropyl	2.5-200	0.9906	2.5-200	0.9933	2.5-200	0.9967	2.5-100	0.9950	2.5-100	0.9965
32	Benzobicyclon	2.5-200	0.9918	2.5-200	0.9959	2.5-200	0.9996	2.5-200	0.9935	2.5-250	0.9903
33	Benzoximate	2.5-200	0.9906	2.5-200	0.9929	2.5-200	0.9960	2.5-200	0.9937	5-100	0.9938
34	Benzoylprop-ethyl	2.5-100	0.9955	2.5-200	0.9925	20-200	0.9958	2.5-200	0.9920	2.5-100	0.9951
35	Benzpyrimoxan	2.5-100	0.9881	5-100	0.9895	2.5-100	0.9920	2.5-200	0.9930	2.5-100	0.9892
36	Benzyladenine	2.5-200	0.9811	2.5-200	0.9885	2.5-200	0.9820	2.5-200	0.9945	5-100	0.9917
37	Bifenox	2.5-100	0.9886	2.5-100	0.9895	10-200	0.9893	2.5-200	0.9871	5-250	0.9829
38	Bifenthrin	5-200	0.9972	2.5-200	0.9987	2.5-200	0.9989	2.5-200	0.9966	2.5-250	0.9961
39	Bispyribac	2.5-200	0.9827	2.5-200	0.9835	10-200	0.9957	2.5-100	0.9890	5-100	0.9898
40	Bistrifluron	2.5-200	0.9915	2.5-200	0.9950	2.5-200	0.9939	2.5-200	0.9980	2.5-250	0.9917
41	Bitertanol	2.5-200	0.9996	2.5-200	0.9998	2.5-200	0.9995	2.5-100	0.9984	5-100	0.9997
42	Bixafen	5-100	0.9830	2.5-200	0.9832	2.5-100	0.9900	5-100	0.9842	5-100	0.9852
43	Boscalid	2.5-100	0.9970	5-100	0.9944	2.5-100	0.9959	5-100	0.9944	5-100	0.9967
44	Bromacil	2.5-200	0.9950	2.5-200	0.9984	2.5-200	0.9962	2.5-200	0.9958	2.5-250	0.9961
45	Bromobutide	5-100	0.9916	2.5-100	0.9867	2.5-200	0.9931	2.5-100	0.9903	2.5-100	0.9920
46	Bupirimate	2.5-100	0.9926	2.5-100	0.9930	2.5-200	0.9935	2.5-100	0.9918	5-100	0.9949
47	Buprofezin	2.5-200	0.9923	2.5-200	0.9945	2.5-200	0.9913	2.5-200	0.9972	2.5-100	0.9970
48	Butachlor	2.5-200	0.9939	2.5-200	0.9963	5-200	0.9953	2.5-200	0.9990	2.5-250	0.9947
49	Butafenacil	2.5-100	0.9910	10-160	0.9871	2.5-200	0.9935	2.5-100	0.9953	2.5-100	0.9963

No.	Compound name	Potato Linear range (μg/kg)	Potato r^2	Cabbage Linear range (μg/kg)	Cabbage r^2	Mandarin Linear range (μg/kg)	Mandarin r^2	Brown rice Linear range (μg/kg)	Brown rice r^2	Soybean Linear range (μg/kg)	Soybean r^2
50	Butocarboxim	2.5-200	0.9815	20-200	0.9848	2.5-100	0.9886	20-200	0.9907	2.5-100	0.9920
51	Butralin	2.5-200	0.9987	2.5-200	0.9990	2.5-200	0.9990	2.5-200	0.9991	2.5-250	0.9994
52	Butylate	2.5-200	0.9986	2.5-200	0.9993	2.5-200	0.9988	2.5-200	0.9990	2.5-250	0.9990
53	Cadusafos	2.5-100	0.9938	2.5-100	0.9950	2.5-200	0.9898	2.5-200	0.9906	2.5-250	0.9871
54	Cafenstrole	2.5-200	0.9841	2.5-200	0.9886	2.5-100	0.9961	2.5-200	0.9888	5-100	0.9954
55	Carbaryl	20-200	0.9904	2.5-200	0.9952	20-200	0.9931	2.5-160	0.9973	2.5-100	0.9978
56	Carbendazim	2.5-200	0.9954	2.5-200	0.9943	20-100	0.9685	2.5-200	0.9951	2.5-250	0.9943
57	Carbetamide	2.5-200	0.9965	2.5-200	0.9961	2.5-100	0.9976	2.5-200	0.9963	2.5-250	0.9938
58	Carbofuran	5-100	0.9884	2.5-100	0.9855	2.5-100	0.9960	5-100	0.9923	2.5-100	0.9899
59	Carbofuran-3-hydroxy	5-100	0.9948	2.5-200	0.9861	5-160	0.9733	2.5-100	0.9957	2.5-100	0.9952
60	Carbophenothion	2.5-200	0.9983	2.5-200	0.9993	2.5-200	0.9992	2.5-200	0.9974	5-100	0.9997
61	Carboxin	2.5-100	0.9947	2.5-200	0.9892	2.5-200	0.9900	2.5-200	0.9903	25-100	0.9965
62	Carfentrazone-ethyl	2.5-100	0.9960	2.5-200	0.9901	2.5-200	0.9903	2.5-200	0.9922	2.5-250	0.9904
63	Carpropamide	5-100	0.9905	2.5-100	0.9866	2.5-200	0.9839	5-100	0.9877	5-100	0.9873
64	Chlorantraniliprole	2.5-200	0.9952	2.5-200	0.9916	2.5-200	0.9988	2.5-200	0.9946	2.5-250	0.9952
65	Chlorbufam	2.5-100	0.9953	5-160	0.9943	20-200	0.9956	5-100	0.9954	10-250	0.9951
66	Chlorfenapyr	10-200	0.9898	5-100	0.9962	5-100	0.9932	5-200	0.9958	2.5-250	0.9975
67	Chlorfenvinphos	2.5-200	0.9909	2.5-200	0.9927	2.5-200	0.9941	2.5-100	0.9948	2.5-250	0.9927
68	Chlorfluazuron	2.5-200	0.9996	2.5-200	0.9993	2.5-200	0.9994	5-200	0.9975	2.5-250	0.9991
69	Chlorflurenol-methyl	5-100	0.9981	10-200	0.9965	5-200	0.9907	2.5-100	0.9914	5-100	0.9950
70	Chloridazon	5-100	0.9836	2.5-100	0.9852	2.5-100	0.9952	5-100	0.9873	5-100	0.9877
71	Chlorimuron-ethyl	2.5-200	0.9990	2.5-100	0.9994	2.5-200	0.9994	2.5-200	0.9949	2.5-250	0.9989
72	Chlorobenzuron	5-100	0.9915	2.5-200	0.9806	2.5-200	0.9823	2.5-200	0.9841	2.5-100	0.9868
73	Chlorotoluron	2.5-200	0.9861	2.5-160	0.9866	2.5-200	0.9838	2.5-100	0.9905	2.5-250	0.9833
74	Chloroxuron	2.5-100	0.9912	2.5-160	0.9853	2.5-200	0.9857	2.5-100	0.9907	5-100	0.9927
75	Chlorpyrifos	2.5-200	0.9981	2.5-200	0.9997	2.5-200	0.9977	2.5-200	0.9991	2.5-100	0.9994
76	Chlorpyrifos-methyl	2.5-200	0.9985	2.5-200	0.9994	2.5-200	0.9989	2.5-200	0.9982	2.5-250	0.9995
77	Chlorthiophos	2.5-200	0.9987	2.5-200	0.9996	2.5-200	0.9993	2.5-200	0.9986	2.5-100	0.9997
78	Chromafenozide	2.5-200	0.9821	2.5-160	0.9872	2.5-200	0.9878	2.5-100	0.9925	5-100	0.9922
79	Cinmethylin	2.5-200	0.9928	2.5-200	0.9951	10-200	0.9960	2.5-200	0.9984	5-250	0.9946
80	Clethodim	2.5-200	0.9979	2.5-200	0.9983	2.5-200	0.9940	2.5-200	0.9983	2.5-100	0.9980
81	Clethodim sulfone	2.5-200	0.9978	5-100	0.9996	2.5-200	0.9928	2.5-200	0.9954	2.5-250	0.9976
82	Clethodim sulfoxide	2.5-200	0.9945	2.5-200	0.9935	2.5-100	0.9994	2.5-200	0.9958	2.5-250	0.9958
83	Clofentezine	2.5-100	0.9944	2.5-200	0.9908	2.5-200	0.9919	2.5-200	0.9945	2.5-250	0.9883
84	Clomazone	2.5-100	0.9930	2.5-100	0.9911	2.5-200	0.9897	2.5-100	0.9933	2.5-100	0.9953
85	Clomeprop	5-100	0.9917	2.5-200	0.9840	2.5-200	0.9827	2.5-200	0.9929	2.5-100	0.9929
86	Coumaphos	2.5-100	0.9950	5-100	0.9951	2.5-200	0.9950	5-100	0.9947	5-100	0.9950
87	Crufomate	5-100	0.9914	2.5-100	0.9904	2.5-200	0.9816	2.5-100	0.9889	2.5-100	0.9905
88	Cyanazine	2.5-200	0.9992	2.5-200	0.9983	2.5-200	0.9878	2.5-100	0.9982	2.5-250	0.9981
89	Cyanophos	2.5-100	0.9814	2.5-160	0.9873	10-160	0.9883	2.5-100	0.9826	5-100	0.9869
90	Cyantraniliprole	2.5-200	0.9982	2.5-200	0.9991	2.5-200	0.9985	2.5-200	0.9991	2.5-250	0.9986
91	Cyazofamid	2.5-100	0.9832	2.5-100	0.9801	2.5-200	0.9950	5-100	0.9859	2.5-100	0.9869
92	Cyclaniliprole	2.5-200	0.9951	2.5-200	0.9951	2.5-200	0.9921	5-100	0.9942	2.5-100	0.9974
93	Cycloate	2.5-200	0.9979	2.5-200	0.9987	2.5-200	0.9985	2.5-200	0.9981	5-100	0.9984
94	Cycloprothrin	2.5-200	0.9994	2.5-200	0.9997	2.5-200	0.9995	2.5-200	0.9996	2.5-250	0.9995
95	Cyclosulfamuron	2.5-200	0.9941	2.5-200	0.9946	2.5-200	0.9963	2.5-200	0.9930	2.5-250	0.9938
96	Cyenopyrafen	2.5-200	0.9942	2.5-200	0.9967	2.5-200	0.9962	2.5-100	0.9979	2.5-250	0.9970
97	Cyflufenamid	2.5-100	0.9954	5-100	0.9969	2.5-100	0.9973	5-100	0.9935	2.5-100	0.9947
98	Cyflumetofen	2.5-200	0.9964	5-100	0.9989	2.5-200	0.9971	5-200	0.9961	10-250	0.9975
99	Cyhalofop-butyl	20-200	0.9932	2.5-200	0.9969	5-200	0.9932	2.5-200	0.9944	5-250	0.9966
100	Cyhalothrin	2.5-200	0.9966	2.5-200	0.9979	2.5-200	0.9973	5-200	0.9984	2.5-250	0.9966

No.	Compound name	Potato		Cabbage		Mandarin		Brown rice		Soybean	
		Linear range (μg/kg)	r^2	Linear range (μg/kg)	r^2	Linear range (μg/kg)	r^2	Linear range (μg/kg)	r^2	Linear range (μg/kg)	r^2
101	Cymoxanil	2.5-200	0.9877	2.5-200	0.9915	10-200	0.9982	2.5-200	0.9934	5-250	0.9858
102	Cyprazine	2.5-100	0.9877	5-100	0.9889	2.5-100	0.9950	2.5-100	0.9887	2.5-100	0.9919
103	Cyproconazole	2.5-200	0.9851	2.5-160	0.9900	2.5-200	0.9888	2.5-100	0.9953	2.5-100	0.9934
104	Cyprodinil	2.5-200	0.9934	2.5-200	0.9944	2.5-200	0.9936	2.5-200	0.9939	2.5-250	0.9941
105	Cyromazine	2.5-200	0.9899	5-100	0.9858	10-200	0.9873	2.5-200	0.9949	5-250	0.9813
106	Daimuron (Dymron)	2.5-100	0.9883	2.5-100	0.9883	2.5-200	0.9918	2.5-100	0.9878	5-100	0.9885
107	Deltamethrin	2.5-200	0.9994	2.5-200	0.9997	2.5-200	0.9996	10-200	0.9990	2.5-250	0.9997
108	Demeton-S	2.5-200	0.9949	2.5-200	0.9959	2.5-200	0.9977	2.5-200	0.9959	2.5-250	0.9911
109	Demeton-S sulfone	2.5-100	0.9907	5-200	0.9909	2.5-200	0.9848	5-100	0.9960	2.5-100	0.9947
110	Demeton-S sulfoxide	2.5-100	0.9912	5-200	0.9826	2.5-100	0.9869	5-100	0.9985	2.5-100	0.9980
111	Demeton-S-methyl	20-200	0.9836	20-200	0.9845	20-200	0.9959	20-200	0.9858	25-250	0.9924
112	Demeton-S-methylsulfone	2.5-100	0.9902	2.5-100	0.9938	2.5-200	0.9860	2.5-100	0.9919	2.5-250	0.9829
113	Desmetryn	2.5-200	0.9928	2.5-200	0.9938	2.5-200	0.9933	2.5-200	0.9947	2.5-250	0.9942
114	Dialifor	2.5-100	0.9932	5-100	0.9937	2.5-100	0.9958	2.5-100	0.9938	2.5-250	0.9857
115	Di-allate	2.5-200	0.9984	2.5-200	0.9974	2.5-200	0.9992	2.5-200	0.9987	2.5-250	0.9988
116	Diazinon	2.5-200	0.9950	2.5-200	0.9959	2.5-200	0.9963	2.5-200	0.9960	2.5-250	0.9968
117	Dichlobenil	5-100	0.9904	10-200	0.9930	10-200	0.9914	10-200	0.9909	5-250	0.9907
118	Dichlofenthion	2.5-200	0.9973	2.5-200	0.9985	2.5-200	0.9982	2.5-200	0.9983	2.5-250	0.9982
119	Dichlormid	2.5-200	0.9941	2.5-200	0.9976	2.5-200	0.9942	2.5-200	0.9982	2.5-250	0.9965
120	Dichlorvos	20-200	0.9931	2.5-200	0.9921	2.5-200	0.9938	20-100	0.9937	25-250	0.9890
121	Diclobutrazol	2.5-200	0.9931	5-100	0.9977	2.5-200	0.9940	5-100	0.9965	2.5-250	0.9925
122	Diclocymet	5-100	0.9912	5-100	0.9917	5-100	0.9929	5-100	0.9894	2.5-100	0.9915
123	Diclofop-methyl	20-200	0.9840	5-200	0.9865	10-200	0.9918	5-200	0.9959	2.5-250	0.9868
124	Diclosulam	2.5-200	0.9968	2.5-100	0.9976	2.5-200	0.9985	5-200	0.9970	2.5-100	0.9986
125	Dicrotophos	2.5-100	0.9973	5-100	0.9981	2.5-200	0.9914	5-100	0.9970	2.5-250	0.9963
126	Dicyclanil	2.5-200	0.9961	2.5-200	0.9971	2.5-200	0.9913	2.5-200	0.9884	2.5-100	0.9961
127	Diethatyl-ethyl	2.5-100	0.9911	2.5-100	0.9903	2.5-200	0.9865	2.5-100	0.9924	2.5-100	0.9915
128	Diethofencarb	20-100	0.9926	5-100	0.9902	2.5-100	0.9962	2.5-100	0.9934	5-100	0.9952
129	Difenoconazole	2.5-100	0.9978	5-100	0.9981	2.5-200	0.9958	5-100	0.9975	2.5-250	0.9939
130	Diflubenzuron	2.5-200	0.9865	5-100	0.9930	2.5-200	0.9905	2.5-100	0.9930	2.5-100	0.9929
131	Diflufenican	2.5-100	0.9983	5-100	0.9983	2.5-200	0.9968	2.5-100	0.9980	2.5-250	0.9958
132	Dimepiperate	2.5-200	0.9962	2.5-200	0.9964	2.5-200	0.9968	2.5-200	0.9973	5-100	0.9971
133	Dimethametryn	2.5-200	0.9952	2.5-200	0.9943	2.5-200	0.9948	2.5-200	0.9957	2.5-250	0.9945
134	Dimethenamid	2.5-100	0.9917	2.5-100	0.9919	2.5-100	0.9950	2.5-100	0.9918	2.5-100	0.9950
135	Dimethoate	2.5-100	0.9912	5-100	0.9923	2.5-100	0.9971	5-100	0.9940	5-100	0.9941
136	Dimethylvinphos	5-100	0.9905	2.5-100	0.9856	2.5-200	0.9942	2.5-100	0.9880	2.5-100	0.9902
137	Dinitramine	2.5-200	0.9970	2.5-200	0.9978	2.5-200	0.9972	2.5-200	0.9982	2.5-250	0.9983
138	Dioxathion	2.5-100	0.9809	5-100	0.9835	2.5-100	0.9884	5-200	0.9838	25-100	0.9905
139	Diphenamid	2.5-100	0.9912	2.5-100	0.9918	2.5-200	0.9917	2.5-100	0.9888	2.5-100	0.9931
140	Disulfoton sulfone	2.5-100	0.9945	2.5-100	0.9931	2.5-200	0.9848	5-100	0.9928	5-100	0.9954
141	Disulfoton sulfoxide	2.5-100	0.9936	2.5-100	0.9925	2.5-100	0.9926	5-100	0.9926	2.5-100	0.9948
142	Dithiopyr	2.5-200	0.9945	2.5-100	0.9983	2.5-200	0.9956	2.5-200	0.9964	2.5-250	0.9978
143	Diuron	5-100	0.9883	2.5-100	0.9878	2.5-100	0.9882	5-100	0.9858	2.5-100	0.9896
144	DNOC	2.5-200	0.9995	2.5-200	0.9990	2.5-200	0.9961	2.5-200	0.9988	2.5-250	0.9996
145	Dodine	2.5-100	0.9840	5-100	0.9853	5-200	0.9822	5-100	0.9838	2.5-100	0.9856
146	Edifenphos	5-100	0.9916	5-100	0.9918	2.5-100	0.9907	5-100	0.9918	2.5-250	0.9806
147	Emamectin B1a	2.5-200	0.9992	2.5-200	0.9995	2.5-200	0.9994	2.5-200	0.9994	2.5-250	0.9995
148	Epoxiconazole	2.5-100	0.9904	5-100	0.9908	2.5-200	0.9961	5-100	0.9897	5-100	0.9940
149	EPTC	2.5-200	0.9972	2.5-200	0.9983	2.5-200	0.9990	2.5-200	0.9992	2.5-250	0.9969
150	Esprocarb	2.5-160	0.9904	2.5-200	0.9928	2.5-200	0.9915	2.5-200	0.9973	2.5-250	0.9921
151	Etaconazole	2.5-100	0.9924	2.5-100	0.9936	2.5-200	0.9932	2.5-100	0.9921	2.5-100	0.9937

No.	Compound name	Potato		Cabbage		Mandarin		Brown rice		Soybean	
		Linear range (μg/kg)	r^2	Linear range (μg/kg)	r^2	Linear range (μg/kg)	r^2	Linear range (μg/kg)	r^2	Linear range (μg/kg)	r^2
152	Ethaboxam	2.5-100	0.9896	5-100	0.9866	2.5-200	0.9913	5-100	0.9861	5-100	0.9874
153	Ethametsulfuron-methyl	2.5-200	0.9949	2.5-200	0.9955	5-200	0.9959	2.5-200	0.9939	2.5-250	0.9943
154	Ethiofencarb	2.5-200	0.9873	2.5-100	0.9945	5-200	0.9908	2.5-200	0.9921	2.5-100	0.9966
155	Ethion	2.5-200	0.9929	2.5-200	0.9963	2.5-200	0.9930	2.5-200	0.9960	2.5-250	0.9923
156	Ethofumesate	2.5-100	0.9966	2.5-200	0.9942	2.5-200	0.9926	2.5-200	0.9933	2.5-250	0.9907
157	Ethofumesate metabolte (NC 20645)	2.5-200	0.9417	2.5-200	0.9823	2.5-100	0.9851	2.5-200	0.9964	2.5-100	0.9936
158	Ethoprophos (Ethoprop)	5-100	0.9901	2.5-100	0.9867	2.5-100	0.9951	2.5-100	0.9855	2.5-100	0.9908
159	Ethychlozate	2.5-200	0.9908	2.5-200	0.9922	2.5-200	0.9912	2.5-100	0.9936	25-100	0.9967
160	Etofenprox	2.5-200	0.9977	2.5-200	0.9996	2.5-200	0.9996	2.5-200	0.9992	2.5-100	0.9993
161	Etrimfos	2.5-200	0.9929	2.5-200	0.9947	2.5-200	0.9947	2.5-100	0.9965	2.5-250	0.9939
162	Famoxadone	2.5-200	0.9975	5-200	0.9983	2.5-200	0.9981	2.5-200	0.9980	2.5-250	0.9980
163	Fenamidone	2.5-200	0.9920	2.5-200	0.9940	2.5-200	0.9965	2.5-100	0.9960	2.5-250	0.9892
164	Fenamiphos	2.5-100	0.9905	2.5-100	0.9900	2.5-200	0.9805	5-100	0.9926	2.5-100	0.9908
165	Fenarimol	2.5-200	0.9981	2.5-200	0.9986	2.5-200	0.9985	2.5-100	0.9992	2.5-250	0.9986
166	Fenazaquin	2.5-200	0.9923	2.5-200	0.9965	2.5-200	0.9955	2.5-200	0.9988	2.5-250	0.9970
167	Fenbuconazole	2.5-200	0.9953	2.5-200	0.9962	2.5-200	0.9961	2.5-100	0.9975	2.5-250	0.9950
168	Fenfuram	2.5-100	0.9965	2.5-200	0.9927	2.5-200	0.9911	2.5-200	0.9936	2.5-250	0.9903
169	Fenhexamid	2.5-200	0.9937	2.5-200	0.9945	2.5-200	0.9948	2.5-200	0.9949	2.5-250	0.9939
170	Fenobucarb	2.5-100	0.9942	2.5-100	0.9923	2.5-200	0.9902	2.5-200	0.9912	2.5-250	0.9886
171	Fenothiocarb	2.5-100	0.9903	2.5-100	0.9899	2.5-200	0.9886	2.5-100	0.9913	2.5-250	0.9804
172	Fenoxanil	2.5-100	0.9940	2.5-100	0.9927	2.5-100	0.9944	2.5-200	0.9907	2.5-100	0.9927
173	Fenoxaprop-ethyl	2.5-100	0.9943	2.5-200	0.9901	2.5-200	0.9878	2.5-100	0.9950	2.5-250	0.9884
174	Fenoxycarb	2.5-200	0.9874	2.5-200	0.9906	2.5-200	0.9922	2.5-100	0.9944	2.5-100	0.9951
175	Fenpropathrin	2.5-200	0.9984	2.5-200	0.9983	2.5-200	0.9982	2.5-200	0.9969	2.5-250	0.9986
176	Fenpropimorph	2.5-200	0.9959	2.5-200	0.9954	2.5-200	0.9974	2.5-200	0.9971	2.5-250	0.9958
177	Fenpyrazamine	2.5-200	0.9888	2.5-100	0.9940	2.5-200	0.9965	2.5-100	0.9912	2.5-250	0.9859
178	Fenpyroximate	2.5-200	0.9971	2.5-200	0.9981	2.5-200	0.9975	2.5-200	0.9989	2.5-250	0.9981
179	Fensulfothion	5-100	0.9915	2.5-100	0.9898	2.5-100	0.9921	2.5-100	0.9899	5-100	0.9936
180	Fenthion	2.5-100	0.9938	2.5-100	0.9935	2.5-200	0.9915	2.5-100	0.9932	2.5-250	0.9867
181	Fentrazamide	2.5-100	0.9923	2.5-100	0.9909	2.5-200	0.9936	2.5-100	0.9927	2.5-250	0.9860
182	Ferimzone	2.5-200	0.9930	2.5-200	0.9928	2.5-100	0.9971	2.5-100	0.9959	2.5-250	0.9918
183	Fipronil	5-100	0.9865	5-160	0.9857	20-200	0.9923	2.5-100	0.9890	5-100	0.9818
184	Flamprop-isopropyl	2.5-100	0.9934	2.5-100	0.9924	2.5-200	0.9913	2.5-100	0.9926	2.5-100	0.9919
185	Flazasulfuron	2.5-100	0.9943	2.5-100	0.9913	2.5-100	0.9969	5-100	0.9922	2.5-100	0.9942
186	Flonicamid	2.5-200	0.9918	2.5-200	0.9957	2.5-200	0.9906	2.5-200	0.9936	5-100	0.9958
187	Florpyrauxifen	2.5-200	0.9993	2.5-200	0.9980	10-200	0.9997	2.5-200	0.9984	10-250	0.9995
188	Fluacrypyrim	2.5-200	0.9954	2.5-200	0.9977	2.5-200	0.9980	2.5-200	0.9974	2.5-250	0.9968
189	Fluazifop Butyl	2.5-160	0.9884	2.5-200	0.9892	2.5-200	0.9891	2.5-200	0.9905	2.5-250	0.9898
190	Fluazinam	2.5-200	0.9987	2.5-200	0.9998	2.5-200	0.9989	2.5-200	0.9993	2.5-200	0.9988
191	Flubendiamide	2.5-100	0.9934	2.5-100	0.9904	2.5-200	0.9887	5-100	0.9929	2.5-100	0.9910
192	Flucetosulfuron	2.5-200	0.9968	2.5-200	0.9975	2.5-200	0.9976	2.5-200	0.9967	2.5-250	0.9963
193	Flucythrinate	2.5-200	0.9988	2.5-200	0.9997	2.5-200	0.9996	2.5-200	0.9997	2.5-250	0.9997
194	Fludioxonil	5-100	0.9896	5-100	0.9891	2.5-200	0.9809	2.5-100	0.9890	2.5-100	0.9917
195	Flufenacet	5-100	0.9913	5-100	0.9906	2.5-200	0.9860	5-100	0.9907	2.5-100	0.9915
196	Flufenoxuron	2.5-200	0.9983	2.5-200	0.9993	2.5-200	0.9990	2.5-200	0.9988	2.5-250	0.9992
197	Flufenpyr-ethyl	20-100	0.9945	2.5-100	0.9938	2.5-200	0.9896	2.5-100	0.9935	2.5-100	0.9951
198	Flumetralin	2.5-200	0.9979	5-200	0.9967	10-200	0.9987	20-200	0.9903	10-100	0.9975
199	Flumioxazin	2.5-200	0.9904	5-200	0.9915	10-200	0.9971	2.5-100	0.9961	2.5-100	0.9963
200	Fluometuron	5-100	0.9902	2.5-100	0.9898	2.5-200	0.9910	2.5-100	0.9893	2.5-100	0.9925
201	Fluopicolide	2.5-100	0.9917	2.5-100	0.9921	2.5-200	0.9893	2.5-100	0.9912	2.5-100	0.9940
202	Fluopyram	2.5-100	0.9905	2.5-100	0.9928	2.5-200	0.9868	2.5-100	0.9911	2.5-100	0.9910

No.	Compound name	Potato		Cabbage		Mandarin		Brown rice		Soybean	
		Linear range (μg/kg)	r^2	Linear range (μg/kg)	r^2	Linear range (μg/kg)	r^2	Linear range (μg/kg)	r^2	Linear range (μg/kg)	r^2
203	Flupoxam	2.5-200	0.9960	2.5-200	0.9982	2.5-200	0.9982	2.5-100	0.9984	2.5-100	0.9983
204	Flupyradifurone	2.5-100	0.9931	2.5-100	0.9917	2.5-100	0.9972	2.5-100	0.9953	2.5-100	0.9953
205	Fluquinconazole	2.5-100	0.9959	2.5-200	0.9922	2.5-200	0.9918	2.5-200	0.9954	2.5-250	0.9915
206	Fluridone	2.5-100	0.9954	2.5-200	0.9875	2.5-200	0.9866	2.5-100	0.9928	2.5-100	0.9951
207	Flurochloridone	2.5-100	0.9921	2.5-100	0.9919	2.5-200	0.9878	2.5-100	0.9898	2.5-100	0.9940
208	Flurtamone	2.5-100	0.9923	5-100	0.9899	5-100	0.9977	2.5-100	0.9931	5-100	0.9952
209	Flusilazole	2.5-100	0.9905	2.5-100	0.9913	2.5-100	0.9937	2.5-100	0.9912	2.5-100	0.9923
210	Flusulfamide	2.5-100	0.9877	5-100	0.9902	2.5-200	0.9835	2.5-200	0.9855	2.5-250	0.9823
211	Fluthiacet-methyl	20-200	0.9923	2.5-200	0.9953	2.5-200	0.9971	2.5-200	0.9936	2.5-250	0.9946
212	Flutianil	5-100	0.9918	5-100	0.9867	2.5-100	0.9938	5-100	0.9895	5-100	0.9860
213	Flutolanil	5-100	0.9884	5-100	0.9890	2.5-200	0.9931	5-100	0.9873	5-100	0.9922
214	Flutriafol	2.5-200	0.9951	2.5-200	0.9961	2.5-200	0.9973	2.5-200	0.9955	2.5-250	0.9940
215	Fluvalinate	2.5-200	0.9994	2.5-200	0.9995	2.5-200	0.9996	2.5-200	0.9987	5-100	0.9995
216	Fluxametamide	2.5-200	0.9928	2.5-200	0.9918	2.5-200	0.9955	2.5-200	0.9948	2.5-250	0.9913
217	Fluxapyroxad	2.5-100	0.9836	2.5-100	0.9854	2.5-100	0.9911	2.5-100	0.9840	5-100	0.9902
218	Fomesafen	2.5-200	0.9993	2.5-200	0.9991	2.5-200	0.9993	2.5-200	0.9996	2.5-250	0.9996
219	Fonofos	2.5-200	0.9969	2.5-200	0.9979	2.5-200	0.9986	2.5-200	0.9978	2.5-250	0.9979
220	Foramsulfuron	2.5-200	0.9981	2.5-200	0.9963	2.5-200	0.9979	2.5-200	0.9915	2.5-250	0.9915
221	Forchlorfenuron	2.5-200	0.9919	2.5-200	0.9949	2.5-200	0.9982	2.5-200	0.9942	2.5-250	0.9879
222	Fosthiazate	2.5-200	0.9924	2.5-100	0.9962	2.5-200	0.9940	2.5-200	0.9946	2.5-100	0.9976
223	Furathiocarb	2.5-200	0.9954	2.5-200	0.9960	2.5-200	0.9956	2.5-200	0.9960	2.5-250	0.9969
224	GPTC	2.5-100	0.9962	2.5-200	0.9971	10-200	0.9948	2.5-200	0.9824	5-100	0.9678
225	Halfenprox	2.5-200	0.9980	2.5-200	0.9990	2.5-200	0.9993	2.5-200	0.9947	2.5-250	0.9996
226	Halosulfuron-methyl	2.5-200	0.9989	2.5-200	0.9985	2.5-200	0.9986	2.5-200	0.9982	2.5-250	0.9989
227	Heptenophos	20-100	0.9939	2.5-100	0.9943	2.5-200	0.9906	2.5-200	0.9918	2.5-100	0.9956
228	Hexaconazole	2.5-200	0.9923	2.5-200	0.9933	2.5-200	0.9949	2.5-200	0.9930	2.5-250	0.9924
229	Hexaflumuron	2.5-200	0.9949	2.5-100	0.9968	2.5-200	0.9973	2.5-200	0.9956	2.5-250	0.9974
230	Hexazinone	5-100	0.9908	5-100	0.9911	2.5-100	0.9965	2.5-200	0.9843	2.5-100	0.9920
231	Hexythiazox	2.5-200	0.9975	2.5-200	0.9988	2.5-200	0.9974	2.5-200	0.9988	5-100	0.9994
232	Imazalil	2.5-200	0.9953	2.5-200	0.9940	2.5-200	0.9924	2.5-200	0.9958	2.5-250	0.9951
233	Imazamethabenz-methyl	2.5-100	0.9944	5-100	0.9946	2.5-100	0.9969	2.5-200	0.9916	5-100	0.9956
234	Imazamox	2.5-200	0.9934	2.5-200	0.9941	2.5-200	0.9953	2.5-200	0.9928	2.5-250	0.9918
235	Imazamox metabolite (M720H001)	2.5-200	0.9939	2.5-200	0.9896	20-200	0.9947	2.5-200	0.9845	10-250	0.9974
236	Imazapic	2.5-200	0.9904	2.5-200	0.9916	2.5-200	0.9878	2.5-200	0.9908	5-100	0.9516
237	Imazaquin	2.5-100	0.9935	2.5-200	0.9921	2.5-200	0.9915	2.5-200	0.9934	2.5-250	0.9870
238	Imazethapyr	2.5-200	0.9972	2.5-200	0.9904	2.5-200	0.9909	2.5-200	0.9976	2.5-250	0.9875
239	Imazosulfuron	2.5-200	0.9980	2.5-100	0.9985	5-100	0.9993	2.5-200	0.9948	2.5-100	0.9989
240	Imibenconazole	2.5-200	0.9983	2.5-200	0.9991	2.5-200	0.9986	2.5-200	0.9970	2.5-250	0.9989
241	Imicyafos	5-100	0.9924	2.5-100	0.9912	2.5-100	0.9964	2.5-200	0.9917	2.5-250	0.9869
242	Imidacloprid	2.5-200	0.9906	2.5-200	0.9963	2.5-100	0.9974	2.5-200	0.9949	2.5-250	0.9896
243	Inabenfide	2.5-200	0.9997	2.5-200	0.9998	5-200	0.9994	2.5-200	0.9981	5-250	0.9998
244	Indanofan	2.5-100	0.9904	2.5-100	0.9903	2.5-200	0.9978	2.5-200	0.9859	2.5-100	0.9936
245	Indaziflam	5-100	0.9905	5-100	0.9903	2.5-200	0.9904	20-200	0.9850	25-100	0.9907
246	Indoxacarb	2.5-200	0.9979	2.5-200	0.9988	2.5-200	0.9986	2.5-200	0.9976	2.5-250	0.9988
247	Ipconazole	2.5-200	0.9951	2.5-200	0.9979	2.5-200	0.9972	2.5-200	0.9974	2.5-250	0.9976
248	Ipfencarbazone	2.5-100	0.9904	5-100	0.9927	2.5-200	0.9903	2.5-200	0.9820	2.5-100	0.9885
249	Iprobenfos	5-100	0.9912	5-100	0.9906	2.5-100	0.9917	2.5-200	0.9836	5-100	0.9900
250	Iprovalicarb	2.5-100	0.9878	2.5-200	0.9908	2.5-200	0.9936	2.5-100	0.9933	2.5-100	0.9878
251	Isazofos	2.5-100	0.9940	2.5-100	0.9935	2.5-100	0.9931	2.5-100	0.9917	2.5-250	0.9828
252	Isofenphos	2.5-200	0.9956	2.5-200	0.9961	2.5-200	0.9976	2.5-200	0.9975	2.5-250	0.9889
253	Isofenphos-methyl	2.5-200	0.9945	2.5-200	0.9945	5-200	0.9964	2.5-200	0.9945	2.5-100	0.9958

No.	Compound name	Potato		Cabbage		Mandarin		Brown rice		Soybean	
		Linear range (μg/kg)	r^2	Linear range (μg/kg)	r^2	Linear range (μg/kg)	r^2	Linear range (μg/kg)	r^2	Linear range (μg/kg)	r^2
254	Isoprocarb	2.5-200	0.9906	2.5-200	0.9926	5-200	0.9909	2.5-200	0.9942	2.5-250	0.9923
255	Isopropalin	2.5-200	0.9996	2.5-200	0.9998	2.5-200	0.9996	2.5-200	0.9986	2.5-250	0.9998
256	Isoprothiolane	2.5-100	0.9921	2.5-100	0.9925	2.5-200	0.9920	2.5-100	0.9945	5-100	0.9950
257	Isoproturon	2.5-200	0.9897	2.5-100	0.9931	2.5-200	0.9917	2.5-200	0.9912	2.5-100	0.9956
258	Isopyrazam	20-200	0.9906	2.5-100	0.9947	2.5-100	0.9948	2.5-200	0.9919	5-100	0.9937
259	Isotianil	2.5-100	0.9932	2.5-100	0.9901	20-200	0.9964	2.5-100	0.9884	2.5-250	0.9836
260	Isouron	2.5-100	0.9934	2.5-200	0.9856	5-100	0.9961	2.5-200	0.9901	2.5-250	0.9872
261	Isoxaben	2.5-200	0.9886	2.5-100	0.9957	2.5-200	0.9987	2.5-200	0.9912	5-100	0.9952
262	Isoxadifen	2.5-200	0.9993	2.5-200	0.9983	10-200	0.9991	2.5-200	0.9986	5-250	0.9982
263	Isoxadifen-ethyl	5-100	0.9891	2.5-100	0.9823	2.5-100	0.9870	2.5-100	0.9861	2.5-100	0.9855
264	Isoxathion	2.5-200	0.9840	2.5-100	0.9914	2.5-200	0.9862	2.5-100	0.9915	2.5-250	0.9847
265	Ivermectin B1a	2.5-100	0.9866	5-200	0.9950	5-200	0.9972	10-200	0.9960	25-250	0.9992
266	Kresoxim-methyl	2.5-100	0.9918	2.5-100	0.9910	2.5-100	0.9948	2.5-100	0.9952	2.5-100	0.9918
267	Lancotrione	2.5-200	0.9873	2.5-200	0.9910	10-200	0.9908	2.5-200	0.9834	2.5-250	0.9861
268	Lenacil	2.5-200	0.9836	2.5-100	0.9916	5-200	0.9901	2.5-200	0.9888	2.5-250	0.9817
269	Leptophos	2.5-200	0.9974	5-200	0.9989	20-200	0.9917	20-200	0.9838	10-100	0.9967
270	Linuron	2.5-200	0.9908	2.5-100	0.9949	2.5-200	0.9948	2.5-200	0.9953	2.5-250	0.9922
271	Lufenuron	2.5-200	0.9918	2.5-100	0.9962	2.5-200	0.9949	2.5-200	0.9971	2.5-250	0.9928
272	malaoxon	2.5-100	0.9938	2.5-100	0.9926	2.5-200	0.9915	2.5-200	0.9907	2.5-100	0.9952
273	Malathion	2.5-100	0.9905	2.5-100	0.9901	2.5-100	0.9942	2.5-100	0.9903	2.5-100	0.9935
274	Mandestrobin	2.5-200	0.9901	2.5-200	0.9924	2.5-200	0.9946	2.5-200	0.9922	2.5-250	0.9894
275	Mandipropamid	2.5-200	0.9885	2.5-100	0.9932	2.5-200	0.9919	2.5-100	0.9938	5-100	0.9959
276	Mecarbam	2.5-200	0.9885	2.5-200	0.9917	2.5-200	0.9935	2.5-200	0.9933	2.5-250	0.9866
277	Mefenacet	2.5-200	0.9890	2.5-100	0.9941	2.5-200	0.9937	2.5-200	0.9921	2.5-250	0.9867
278	Mefenpyr-diethyl	2.5-200	0.9938	5-100	0.9979	2.5-160	0.9976	2.5-200	0.9951	2.5-250	0.9942
279	Mefentrifluconazole	2.5-200	0.9952	2.5-200	0.9966	2.5-200	0.9977	2.5-200	0.9960	2.5-250	0.9962
280	Mepanipyrim	2.5-100	0.9902	2.5-100	0.9907	2.5-100	0.9927	2.5-100	0.9879	2.5-100	0.9922
281	Mephosfolan	2.5-200	0.9919	2.5-100	0.9949	2.5-200	0.9885	2.5-200	0.9945	2.5-250	0.9905
282	Mepronil	5-100	0.9915	5-100	0.9902	2.5-100	0.9934	2.5-100	0.9875	2.5-100	0.9904
283	Mesosulfuron-methyl	2.5-200	0.9965	2.5-200	0.9962	5-100	0.9972	2.5-200	0.9939	2.5-100	0.9988
284	Mesotrione	2.5-200	0.9986	2.5-200	0.9984	5-200	0.9986	2.5-200	0.9997	5-250	0.9992
285	Metaflumizone	2.5-200	0.9994	2.5-200	0.9995	2.5-200	0.9996	2.5-200	0.9995	2.5-250	0.9994
286	Metamifop	2.5-200	0.9926	2.5-200	0.9949	2.5-200	0.9930	2.5-200	0.9959	2.5-250	0.9940
287	Metamitron	5-100	0.9890	2.5-200	0.9957	2.5-100	0.9977	2.5-100	0.9914	2.5-100	0.9893
288	Metazosulfuron	2.5-200	0.9980	2.5-200	0.9969	2.5-200	0.9973	2.5-200	0.9970	2.5-250	0.9977
289	Metconazole	2.5-200	0.9930	2.5-200	0.9955	2.5-200	0.9951	2.5-200	0.9943	2.5-250	0.9933
290	Methabenzthiazuron	5-100	0.9938	2.5-100	0.9928	2.5-200	0.9865	2.5-100	0.9923	2.5-250	0.9835
291	Methacrifos	2.5-200	0.9971	5-200	0.9956	10-200	0.9976	2.5-200	0.9972	5-100	0.9994
292	Methidathion	5-100	0.9910	2.5-100	0.9886	2.5-200	0.9910	2.5-100	0.9884	5-100	0.9942
293	Methiocarb	2.5-200	0.9903	2.5-200	0.9923	2.5-200	0.9963	2.5-200	0.9922	2.5-250	0.9923
294	Methoprotryn	2.5-100	0.9951	2.5-200	0.9900	5-100	0.9990	2.5-200	0.9916	2.5-100	0.9970
295	Methoxyfenozide	2.5-100	0.9926	2.5-100	0.9923	2.5-100	0.9972	2.5-100	0.9912	2.5-100	0.9941
296	Metolachlor	2.5-200	0.9933	2.5-200	0.9932	2.5-200	0.9944	2.5-200	0.9949	2.5-250	0.9911
297	Metolcarb	2.5-200	0.9931	2.5-200	0.9966	10-200	0.9918	2.5-200	0.9982	2.5-250	0.9953
298	Metominostrobin	2.5-100	0.9911	2.5-100	0.9919	2.5-100	0.9937	2.5-100	0.9904	2.5-100	0.9938
299	Metosulam	2.5-200	0.9961	2.5-200	0.9976	2.5-100	0.9945	2.5-200	0.9942	2.5-250	0.9949
300	Metrafenone	2.5-200	0.9951	2.5-200	0.9970	2.5-200	0.9968	2.5-200	0.9972	2.5-250	0.9961
301	Metribuzin	2.5-100	0.9845	2.5-100	0.9846	5-100	0.9894	2.5-100	0.9874	2.5-100	0.9887
302	Mevinphos	2.5-200	0.9948	2.5-200	0.9960	2.5-200	0.9915	2.5-200	0.9975	2.5-250	0.9953
303	MGK-264	2.5-100	0.9958	2.5-200	0.9935	2.5-200	0.9935	2.5-200	0.9929	2.5-250	0.9928
304	Milbemectin A4	5-200	0.9975	5-200	0.9990	20-200	0.9979	5-200	0.9981	25-250	0.9988

No.	Compound name	Potato		Cabbage		Mandarin		Brown rice		Soybean	
		Linear range (μg/kg)	r^2	Linear range (μg/kg)	r^2	Linear range (μg/kg)	r^2	Linear range (μg/kg)	r^2	Linear range (μg/kg)	r^2
305	Molinate	2.5-200	0.9963	2.5-200	0.9986	2.5-200	0.9985	2.5-200	0.9994	2.5-250	0.9974
306	Monocrotophos	2.5-200	0.9821	2.5-200	0.9919	2.5-200	0.9834	2.5-200	0.9968	2.5-250	0.9825
307	Monolinuron	5-100	0.9925	5-100	0.9904	2.5-100	0.9944	2.5-100	0.9910	5-100	0.9937
308	Myclobutanil	5-100	0.9941	5-100	0.9911	2.5-100	0.9983	2.5-200	0.9921	5-100	0.9963
309	Naftalofos	2.5-200	0.9884	2.5-200	0.9911	2.5-160	0.9902	2.5-200	0.9934	25-250	0.9878
310	Napropamide	5-100	0.9937	2.5-100	0.9909	2.5-200	0.9954	2.5-100	0.9905	2.5-250	0.9828
311	Neburon	5-100	0.9812	5-100	0.9802	5-100	0.9834	5-100	0.9816	5-100	0.9805
312	Nicosulfuron	2.5-200	0.9970	2.5-200	0.9994	5-200	0.9986	2.5-200	0.9993	5-250	0.9991
313	Nitenpyram	2.5-100	0.9926	2.5-200	0.9963	2.5-200	0.9846	2.5-100	0.9912	2.5-100	0.9907
314	Norea (Noruron)	2.5-100	0.9952	2.5-200	0.9907	2.5-200	0.9931	2.5-200	0.9915	2.5-250	0.9902
315	Norflurazon	2.5-100	0.9925	2.5-200	0.9866	2.5-200	0.9905	2.5-100	0.9914	2.5-100	0.9932
316	Novaluron	2.5-200	0.9980	2.5-200	0.9989	2.5-200	0.9989	2.5-200	0.9981	2.5-250	0.9995
317	Nuarimol	2.5-200	0.9991	2.5-200	0.9994	2.5-200	0.9986	2.5-200	0.9990	5-100	0.9996
318	Ofurace	5-100	0.9918	5-100	0.9884	2.5-100	0.9911	5-100	0.9886	2.5-100	0.9906
319	Omethoate	2.5-100	0.9889	5-100	0.9910	2.5-100	0.9918	2.5-200	0.9903	5-100	0.9812
320	Orthosulfamuron	2.5-200	0.9948	2.5-200	0.9944	2.5-200	0.9973	2.5-200	0.9922	2.5-250	0.9931
321	Orysastrobin	2.5-200	0.9946	2.5-200	0.9954	2.5-200	0.9970	2.5-200	0.9971	2.5-100	0.9987
322	Orysastrobin metabolite (F001)	2.5-200	0.9951	2.5-200	0.9954	2.5-200	0.9954	2.5-200	0.9959	2.5-250	0.9937
323	Oryzalin	2.5-200	0.9955	2.5-200	0.9972	2.5-200	0.9991	2.5-200	0.9922	2.5-250	0.9964
324	Oxadiargyl	2.5-100	0.9945	2.5-200	0.9917	2.5-200	0.9906	2.5-200	0.9902	2.5-100	0.9958
325	Oxadiazon	2.5-100	0.9953	2.5-200	0.9928	2.5-200	0.9895	2.5-200	0.9940	2.5-250	0.9888
326	Oxadixyl	2.5-200	0.9908	5-100	0.9965	2.5-200	0.9897	2.5-200	0.9944	2.5-100	0.9975
327	Oxathiapiprolin	2.5-200	0.9966	2.5-200	0.9966	2.5-200	0.9996	2.5-200	0.9951	2.5-100	0.9988
328	Oxaziclomefone	2.5-200	0.9914	2.5-200	0.9941	2.5-200	0.9929	2.5-200	0.9958	2.5-250	0.9931
329	Oxycarboxin	2.5-200	0.9917	2.5-200	0.9919	2.5-100	0.9965	2.5-100	0.9948	5-100	0.9959
330	Oxydemeton-methyl	2.5-100	0.9951	2.5-200	0.9949	2.5-200	0.9902	2.5-200	0.9985	5-100	0.9951
331	Oxyfluorfen	5-100	0.9948	5-100	0.9912	10-100	0.9933	2.5-200	0.9880	5-250	0.9894
332	Paclobutrazol	2.5-100	0.9909	2.5-100	0.9910	2.5-200	0.9934	2.5-100	0.9922	2.5-100	0.9941
333	Parathion	2.5-100	0.9817	2.5-100	0.9829	10-100	0.9935	20-100	0.9801	5-100	0.9883
334	Parathion-Methyl	2.5-100	0.9945	2.5-100	0.9951	5-200	0.9908	2.5-200	0.9917	2.5-100	0.9957
335	Pebulate	2.5-200	0.9979	2.5-200	0.9988	2.5-200	0.9986	2.5-200	0.9987	2.5-250	0.9983
336	Penconazole	2.5-100	0.9902	2.5-100	0.9909	2.5-100	0.9908	2.5-100	0.9909	2.5-100	0.9897
337	Pencycuron	2.5-200	0.9901	2.5-200	0.9925	2.5-200	0.9922	2.5-200	0.9940	2.5-250	0.9908
338	Pendimethalin	2.5-200	0.9980	2.5-200	0.9988	5-100	0.9997	2.5-200	0.9991	2.5-250	0.9993
339	Penflufen	2.5-100	0.9920	5-100	0.9914	2.5-100	0.9928	2.5-100	0.9911	2.5-100	0.9889
340	Penoxsulam	2.5-200	0.9978	2.5-100	0.9977	5-100	0.9974	2.5-200	0.9923	2.5-250	0.9930
341	Penthiopyrad	5-100	0.9892	5-100	0.9890	2.5-100	0.9872	2.5-100	0.9863	2.5-100	0.9874
342	Pentoxazone	2.5-200	0.9900	2.5-200	0.9917	20-200	0.9932	2.5-200	0.9896	5-250	0.9904
343	Permethrin	2.5-200	0.9972	5-200	0.9994	2.5-200	0.9996	5-200	0.9910	2.5-100	0.9997
344	Phenothrin	2.5-200	0.9967	2.5-200	0.9994	2.5-200	0.9985	2.5-200	0.9933	2.5-250	0.9995
345	Phenthoate	5-100	0.9909	5-100	0.9894	2.5-100	0.9904	2.5-100	0.9893	2.5-100	0.9884
346	Phorate oxon	5-100	0.9892	5-100	0.9895	2.5-200	0.9909	2.5-100	0.9884	5-100	0.9922
347	Phorate oxon sulfone	5-100	0.9929	2.5-200	0.9882	2.5-200	0.9853	20-200	0.9921	2.5-100	0.9943
348	Phorate oxon sulfoxide	5-100	0.9962	2.5-200	0.9927	2.5-200	0.9831	5-100	0.9804	2.5-200	0.9449
349	Phorate sulfone	5-100	0.9956	2.5-100	0.9918	2.5-200	0.9854	2.5-100	0.9947	2.5-100	0.9954
350	Phorate sulfoxide	5-100	0.9947	2.5-100	0.9923	2.5-100	0.9913	2.5-100	0.9906	2.5-100	0.9941
351	Phosalone	2.5-100	0.9943	5-100	0.9940	5-100	0.9970	2.5-100	0.9930	2.5-250	0.9876
352	Phosfolan	2.5-100	0.9961	2.5-200	0.9910	2.5-200	0.9853	2.5-200	0.9928	2.5-100	0.9958
353	Phosmet	20-100	0.9931	20-200	0.9875	20-200	0.9870	20-100	0.9907	25-250	0.9831
354	Phosphamidon	2.5-100	0.9971	5-100	0.9979	5-100	0.9991	2.5-200	0.9971	5-100	0.9985
355	Phoxim	2.5-200	0.9882	2.5-200	0.9895	2.5-200	0.9898	2.5-200	0.9911	2.5-250	0.9880

No.	Compound name	Potato		Cabbage		Mandarin		Brown rice		Soybean	
		Linear range (μg/kg)	r^2	Linear range (μg/kg)	r^2	Linear range (μg/kg)	r^2	Linear range (μg/kg)	r^2	Linear range (μg/kg)	r^2
356	Picarbutrazox	2.5-200	0.9957	2.5-200	0.9963	2.5-200	0.9954	2.5-200	0.9988	2.5-250	0.9945
357	Picolinafen	2.5-200	0.9841	2.5-100	0.9920	2.5-100	0.9956	2.5-200	0.9923	2.5-200	0.9839
358	Picoxystrobin	5-100	0.9977	2.5-200	0.9961	2.5-200	0.9967	2.5-200	0.9963	2.5-100	0.9984
359	Pinoxaden	20-200	0.9936	2.5-200	0.9975	2.5-200	0.9958	2.5-200	0.9945	2.5-250	0.9941
360	Pinoxaden metabolite (SYN 505164)	2.5-200	0.9886	2.5-200	0.9879	5-160	0.9988	2.5-200	0.9911	2.5-100	0.9865
361	Piperonyl butoxide	2.5-100	0.9988	2.5-200	0.9982	2.5-200	0.9966	2.5-200	0.9990	2.5-250	0.9967
362	Piperophos	2.5-200	0.9928	2.5-200	0.9964	2.5-200	0.9955	2.5-200	0.9952	2.5-250	0.9948
363	Pirimicarb	2.5-200	0.9907	2.5-200	0.9907	2.5-200	0.9986	2.5-200	0.9913	2.5-100	0.9956
364	Pirimiphos-ethyl	2.5-200	0.9967	2.5-200	0.9971	2.5-200	0.9958	2.5-200	0.9934	2.5-100	0.9992
365	Pirimiphos-methyl	2.5-200	0.9948	2.5-200	0.9966	2.5-200	0.9965	2.5-200	0.9967	2.5-250	0.9963
366	Pretilachlor	2.5-200	0.9952	2.5-200	0.9970	2.5-200	0.9968	2.5-200	0.9976	2.5-250	0.9971
367	Probenazole	2.5-200	0.9969	2.5-200	0.9970	5-200	0.9856	2.5-200	0.9804	2.5-250	0.9888
368	Prochloraz metabolite (BTS 44595)	2.5-100	0.9934	2.5-200	0.9872	2.5-200	0.9904	2.5-100	0.9922	2.5-100	0.9935
369	Procymidone	5-100	0.9894	5-100	0.9885	20-200	0.9987	2.5-100	0.9904	5-100	0.9905
370	Prodiamine	5-100	0.9974	2.5-200	0.9967	2.5-200	0.9943	2.5-200	0.9980	5-100	0.9976
371	Profenofos	2.5-200	0.9867	2.5-100	0.9953	2.5-200	0.9887	2.5-200	0.9927	2.5-250	0.9876
372	Prohydrojasmon	5-100	0.9950	2.5-100	0.9928	2.5-100	0.9957	2.5-100	0.9936	2.5-100	0.9918
373	Promecarb	2.5-200	0.9907	2.5-200	0.9900	2.5-200	0.9990	2.5-200	0.9935	2.5-250	0.9890
374	Prometryn	2.5-200	0.9906	2.5-200	0.9902	2.5-200	0.9905	2.5-200	0.9940	2.5-250	0.9877
375	Propachlor	2.5-100	0.9944	2.5-100	0.9926	2.5-100	0.9963	2.5-200	0.9904	2.5-100	0.9952
376	Propamocarb	2.5-100	0.9847	2.5-200	0.9952	5-100	0.9888	2.5-200	0.9961	2.5-250	0.9866
377	Propanil	2.5-100	0.9949	5-200	0.9886	2.5-100	0.9972	2.5-100	0.9950	2.5-250	0.9882
378	Propargite	2.5-200	0.9960	2.5-200	0.9976	2.5-200	0.9960	2.5-200	0.9979	5-100	0.9991
379	Propazine	2.5-100	0.9932	2.5-200	0.9887	2.5-200	0.9951	2.5-200	0.9953	2.5-250	0.9893
380	Propetamphos	2.5-100	0.9925	2.5-100	0.9905	2.5-200	0.9905	2.5-100	0.9927	2.5-100	0.9947
381	Propiconazole	2.5-100	0.9977	5-100	0.9979	2.5-200	0.9972	2.5-200	0.9960	2.5-250	0.9958
382	Propisochlor	2.5-100	0.9952	2.5-100	0.9920	5-100	0.9943	2.5-200	0.9908	2.5-250	0.9893
383	Propoxur	2.5-100	0.9915	2.5-100	0.9927	10-200	0.9918	2.5-100	0.9933	2.5-100	0.9935
384	Propyrisulfuron	2.5-200	0.9964	2.5-200	0.9977	2.5-100	0.9991	2.5-200	0.9961	2.5-250	0.9969
385	Proquinazid	2.5-200	0.9967	2.5-200	0.9975	2.5-200	0.9969	2.5-200	0.9992	2.5-250	0.9987
386	Prosulfocarb	2.5-200	0.9909	2.5-200	0.9926	2.5-200	0.9914	2.5-200	0.9933	2.5-250	0.9913
387	Prothiofos	2.5-200	0.9974	2.5-200	0.9973	5-200	0.9995	5-200	0.9984	2.5-100	0.9973
388	Pydiflumetofen	2.5-200	0.9852	2.5-200	0.9880	2.5-100	0.9951	2.5-200	0.9898	2.5-100	0.9923
389	Pyflubumide	2.5-200	0.9961	2.5-200	0.9981	2.5-200	0.9961	2.5-200	0.9968	2.5-250	0.9960
390	Pyflubumide-NH	2.5-200	0.9976	2.5-200	0.9984	2.5-200	0.9976	2.5-200	0.9973	2.5-250	0.9988
391	Pyracarbolid	2.5-100	0.9940	2.5-100	0.9864	2.5-100	0.9910	2.5-100	0.9882	2.5-100	0.9897
392	Pyraclofos	2.5-100	0.9963	2.5-100	0.9967	2.5-160	0.9930	2.5-200	0.9901	2.5-100	0.9957
393	Pyraclonil	2.5-100	0.9927	2.5-100	0.9910	2.5-200	0.9904	2.5-100	0.9914	5-100	0.9953
394	Pyraclostrobin	2.5-200	0.9919	2.5-200	0.9938	2.5-200	0.9955	2.5-200	0.9922	2.5-250	0.9915
395	Pyraflufen-ethyl	2.5-100	0.9900	2.5-100	0.9889	2.5-100	0.9951	2.5-100	0.9896	2.5-100	0.9903
396	Pyraziflumid	5-100	0.9879	2.5-100	0.9858	2.5-200	0.9808	2.5-100	0.9853	2.5-100	0.9892
397	Pyrazosulfuron-ethyl	2.5-200	0.9991	2.5-200	0.9995	2.5-200	0.9991	2.5-200	0.9992	2.5-250	0.9993
398	Pyrazoxyfen	2.5-200	0.9874	2.5-200	0.9909	2.5-200	0.9943	2.5-100	0.9938	2.5-100	0.9938
399	Pyribencarb E	2.5-200	0.9925	2.5-200	0.9938	2.5-100	0.9984	2.5-200	0.9947	5-100	0.9939
400	Pyribencarb Z (KIE-9749)	2.5-200	0.9887	2.5-100	0.9893	2.5-200	0.9919	2.5-200	0.9915	2.5-100	0.9884
401	Pyribenzoxim	2.5-200	0.9945	2.5-200	0.9966	2.5-200	0.9947	2.5-200	0.9950	2.5-250	0.9944
402	Pyributicarb	2.5-100	0.9947	2.5-200	0.9912	2.5-200	0.9920	2.5-200	0.9961	5-100	0.9956
403	Pyridaben	2.5-200	0.9943	2.5-200	0.9993	2.5-200	0.9987	2.5-100	0.9913	2.5-100	0.9990
404	Pyridalyl	2.5-200	0.9954	2.5-200	0.9981	2.5-200	0.9990	2.5-200	0.9990	2.5-250	0.9991
405	Pyridaphenthion	2.5-200	0.9837	2.5-200	0.9886	2.5-200	0.9986	2.5-100	0.9902	2.5-100	0.9942
406	Pyrifenox	5-100	0.9915	5-100	0.9915	2.5-100	0.9942	2.5-100	0.9893	2.5-100	0.9920

No.	Compound name	Potato		Cabbage		Mandarin		Brown rice		Soybean	
		Linear range (μg/kg)	r^2	Linear range (μg/kg)	r^2	Linear range (μg/kg)	r^2	Linear range (μg/kg)	r^2	Linear range (μg/kg)	r^2
407	Pyrifluquinazon	2.5-200	0.9920	2.5-200	0.9930	2.5-200	0.9973	2.5-200	0.9934	2.5-100	0.9967
408	Pyriftalid	5-100	0.9913	2.5-200	0.9827	5-100	0.9912	2.5-200	0.9844	2.5-100	0.9908
409	Pyrimethanil	2.5-200	0.9959	2.5-200	0.9961	2.5-200	0.9953	2.5-200	0.9976	2.5-250	0.9962
410	Pyrimethanil-5-hydroxy	5-100	0.9894	2.5-100	0.9864	2.5-200	0.9915	2.5-200	0.9853	2.5-100	0.9906
411	Pyrimidifen	5-100	0.9904	2.5-200	0.9813	2.5-200	0.9814	2.5-200	0.9917	2.5-100	0.9901
412	Pyriminobac-methyl	2.5-100	0.9926	2.5-100	0.9928	2.5-100	0.9929	2.5-200	0.9902	2.5-100	0.9955
413	Pyrimisulfan	2.5-200	0.9944	2.5-200	0.9942	2.5-200	0.9942	2.5-200	0.9937	2.5-250	0.9931
414	Pyriofenone	2.5-200	0.9866	2.5-200	0.9896	2.5-200	0.9898	2.5-100	0.9946	2.5-100	0.9924
415	Pyriproxyfen	2.5-200	0.9917	2.5-200	0.9936	2.5-200	0.9939	2.5-200	0.9965	2.5-250	0.9941
416	Pyroquilon	2.5-200	0.9838	2.5-100	0.9914	5-100	0.9928	2.5-100	0.9924	2.5-250	0.9834
417	Quinalphos	2.5-100	0.9924	2.5-100	0.9897	2.5-100	0.9934	2.5-200	0.9864	2.5-250	0.9810
418	Quinclorac methyl ester	2.5-100	0.9936	2.5-200	0.9908	2.5-200	0.9904	2.5-200	0.9904	2.5-250	0.9889
419	Quinoclamine	2.5-200	0.9870	2.5-100	0.9955	2.5-100	0.9967	2.5-200	0.9926	2.5-250	0.9886
420	Quinoxyfen	2.5-200	0.9965	2.5-200	0.9983	2.5-100	0.9996	2.5-200	0.9987	2.5-250	0.9988
421	Quizalofop-ethyl	2.5-200	0.9889	2.5-100	0.9944	5-200	0.9933	2.5-200	0.9920	2.5-250	0.9924
422	Resmethrin	2.5-200	0.9982	2.5-200	0.9992	2.5-200	0.9985	2.5-200	0.9996	2.5-250	0.9989
423	Rimsulfuron	2.5-200	0.9928	2.5-200	0.9931	2.5-100	0.9988	2.5-200	0.9913	2.5-250	0.9869
424	Saflufenacil	2.5-200	0.9986	2.5-200	0.9972	5-200	0.9985	2.5-200	0.9971	2.5-250	0.9977
425	Sedaxane	5-100	0.9937	2.5-100	0.9912	2.5-200	0.9909	2.5-100	0.9883	2.5-250	0.9801
426	Sethoxydim	2.5-200	0.9907	2.5-200	0.9932	2.5-200	0.9919	2.5-200	0.9978	5-250	0.9928
427	Simazine	5-100	0.9902	2.5-100	0.9894	2.5-100	0.9954	2.5-100	0.9933	2.5-250	0.9819
428	Simeconazole	2.5-100	0.9935	2.5-200	0.9916	2.5-200	0.9918	2.5-200	0.9906	2.5-100	0.9959
429	Simetryn	2.5-200	0.9928	2.5-200	0.9938	2.5-200	0.9933	2.5-200	0.9946	2.5-250	0.9942
430	Spinetoram-J	2.5-200	0.9961	2.5-200	0.9973	2.5-200	0.9966	2.5-200	0.9985	2.5-250	0.9974
431	Spinetoram-L	2.5-200	0.9992	2.5-200	0.9995	2.5-200	0.9994	2.5-200	0.9998	2.5-250	0.9998
432	Spinosyn A	2.5-200	0.9978	2.5-200	0.9987	2.5-200	0.9980	2.5-200	0.9992	2.5-250	0.9974
433	Spinosyn D	2.5-200	0.9976	2.5-200	0.9977	2.5-200	0.9981	2.5-200	0.9984	2.5-250	0.9981
434	Spirodiclofen	2.5-200	0.9971	2.5-200	0.9991	2.5-200	0.9982	2.5-200	0.9986	2.5-250	0.9980
435	Spiromesifen	20-200	0.9947	20-200	0.9976	20-200	0.9978	20-200	0.9982	25-100	0.9986
436	Spirotetramat	2.5-200	0.9945	2.5-200	0.9961	2.5-200	0.9988	2.5-200	0.9930	2.5-250	0.9944
437	Spiroxamine	2.5-200	0.9976	2.5-200	0.9970	2.5-200	0.9982	2.5-200	0.9985	2.5-250	0.9973
438	Sulfentrazone	2.5-200	0.9961	2.5-200	0.9954	2.5-200	0.9950	2.5-200	0.9946	5-100	0.9967
439	Sulfotep	2.5-200	0.9904	2.5-200	0.9914	2.5-200	0.9911	2.5-200	0.9931	2.5-250	0.9907
440	Sulfoxaflor	5-100	0.9901	5-100	0.9899	2.5-100	0.9966	2.5-100	0.9940	2.5-100	0.9928
441	Sulprofos	2.5-200	0.9932	2.5-200	0.9947	2.5-200	0.9948	2.5-200	0.9968	2.5-250	0.9945
442	TCMTB	2.5-200	0.9934	2.5-200	0.9956	2.5-200	0.9979	2.5-200	0.9972	2.5-250	0.9948
443	Tebuconazole	2.5-100	0.9938	5-100	0.9954	5-100	0.9984	2.5-100	0.9943	2.5-100	0.9929
444	Tebufenozide	2.5-200	0.9964	2.5-200	0.9986	2.5-200	0.9963	2.5-200	0.9916	2.5-250	0.9984
445	Tebufenpyrad	2.5-100	0.9946	2.5-200	0.9906	2.5-200	0.9901	2.5-200	0.9945	2.5-250	0.9896
446	Tebufloquin	2.5-200	0.9927	2.5-200	0.9926	2.5-200	0.9928	2.5-200	0.9945	2.5-250	0.9919
447	Tebufloquin metabolite (M1)	5-100	0.9937	2.5-200	0.9878	2.5-200	0.9904	2.5-200	0.9906	2.5-100	0.9942
448	Tebupirimfos	2.5-200	0.9961	2.5-200	0.9980	2.5-200	0.9960	2.5-200	0.9985	2.5-250	0.9966
449	Tebuthiuron	5-100	0.9937	2.5-100	0.9877	2.5-100	0.9935	2.5-100	0.9911	5-100	0.9948
450	Teflubenzuron	2.5-200	0.9838	2.5-100	0.9903	2.5-100	0.9916	2.5-200	0.9920	2.5-100	0.9906
451	Tefuryltrione	2.5-200	0.9943	2.5-200	0.9947	2.5-200	0.9942	2.5-200	0.9959	2.5-250	0.9937
452	TEPP	20-200	0.9951	2.5-200	0.9953	2.5-200	0.9908	20-200	0.9987	25-250	0.9961
453	Tepraloxydim	2.5-200	0.9991	2.5-200	0.9987	5-200	0.9977	2.5-100	0.9948	2.5-250	0.9965
454	Terbacil	2.5-200	0.9991	2.5-200	0.9995	2.5-100	0.9858	2.5-200	0.9996	2.5-250	0.9995
455	Terbufos	2.5-200	0.9924	2.5-200	0.9953	10-200	0.9959	5-200	0.9967	10-250	0.9969
456	Terbufos oxon	2.5-200	0.9816	2.5-200	0.9826	2.5-200	0.9895	2.5-100	0.9908	2.5-100	0.9909
457	Terbufos oxon sulfone	2.5-200	0.9920	2.5-200	0.9904	2.5-200	0.9853	2.5-200	0.9947	2.5-250	0.9928

No.	Compound name	Potato		Cabbage		Mandarin		Brown rice		Soybean	
		Linear range (μg/kg)	r^2	Linear range (μg/kg)	r^2	Linear range (μg/kg)	r^2	Linear range (μg/kg)	r^2	Linear range (μg/kg)	r^2
458	Terbufos oxon sulfoxide	2.5-200	0.9944	2.5-200	0.9972	2.5-200	0.9941	2.5-200	0.9974	2.5-100	0.9992
459	Terbufos sulfone	5-100	0.9930	2.5-100	0.9927	2.5-100	0.9942	2.5-200	0.9889	2.5-100	0.9934
460	Terbufos sulfoxide	2.5-100	0.9940	2.5-200	0.9904	2.5-200	0.9915	2.5-200	0.9925	2.5-100	0.9955
461	Terbuthylazine	5-100	0.9874	5-100	0.9865	2.5-100	0.9910	2.5-200	0.9815	2.5-100	0.9900
462	Terbutryn	2.5-200	0.9911	2.5-200	0.9902	2.5-200	0.9967	2.5-200	0.9935	2.5-250	0.9899
463	Tetrachlorvinphos	5-100	0.9886	5-100	0.9863	2.5-100	0.9903	2.5-100	0.9863	2.5-100	0.9868
464	Tetraconazole	2.5-100	0.9953	2.5-200	0.9918	2.5-200	0.9922	2.5-200	0.9914	2.5-100	0.9964
465	Tetramethrin	2.5-100	0.9948	2.5-100	0.9947	2.5-100	0.9967	2.5-200	0.9932	2.5-250	0.9913
466	Tetraniliprole	2.5-200	0.9990	2.5-200	0.9992	2.5-200	0.9993	2.5-200	0.9985	2.5-100	0.9997
467	Thenylchlor	2.5-200	0.9914	2.5-200	0.9933	2.5-200	0.9945	2.5-200	0.9940	2.5-250	0.9888
468	Thiabendazole	2.5-200	0.9832	2.5-200	0.9910	2.5-200	0.9838	2.5-200	0.9958	2.5-250	0.9862
469	Thiacloprid	5-100	0.9905	5-100	0.9898	2.5-100	0.9947	2.5-100	0.9899	2.5-100	0.9894
470	Thiamethoxam	5-100	0.9888	2.5-100	0.9961	2.5-100	0.9990	2.5-100	0.9928	2.5-100	0.9926
471	Thiazopyr	5-100	0.9906	5-100	0.9902	2.5-100	0.9880	2.5-100	0.9889	2.5-100	0.9899
472	Thidiazuron	2.5-200	0.9933	2.5-200	0.9954	10-200	0.9930	2.5-200	0.9957	5-250	0.9904
473	Thifensulfuron-methyl	2.5-200	0.9988	2.5-200	0.9998	2.5-200	0.9981	2.5-200	0.9994	2.5-250	0.9999
474	Thifluzamide	5-100	0.9926	5-100	0.9913	2.5-100	0.9956	2.5-200	0.9918	2.5-100	0.9958
475	Thiobencarb	5-100	0.9961	2.5-200	0.9901	2.5-100	0.9963	2.5-200	0.9919	2.5-250	0.9891
476	Thiometon	2.5-100	0.9935	5-100	0.9864	20-200	0.9866	2.5-200	0.9845	10-100	0.9902
477	Tiadinil	2.5-200	0.9940	2.5-200	0.9966	2.5-200	0.9968	2.5-200	0.9963	2.5-250	0.9957
478	Tolclofos-methyl	2.5-200	0.9960	2.5-200	0.9970	2.5-200	0.9976	2.5-200	0.9974	2.5-250	0.9973
479	Tolfenpyrad	2.5-200	0.9969	2.5-200	0.9991	2.5-200	0.9984	2.5-200	0.9965	2.5-250	0.9974
480	Tralkoxydim	2.5-200	0.9972	2.5-200	0.9982	2.5-200	0.9952	2.5-200	0.9990	2.5-250	0.9987
481	Triadimefon	5-100	0.9950	5-100	0.9952	5-100	0.9978	2.5-200	0.9912	5-100	0.9961
482	Triadimenol	2.5-200	0.9952	2.5-200	0.9943	5-200	0.9965	2.5-200	0.9943	5-100	0.9977
483	Triafamone	5-100	0.9905	2.5-100	0.9921	2.5-200	0.9930	2.5-100	0.9950	2.5-100	0.9924
484	Tri-allate	2.5-200	0.9990	2.5-200	0.9993	2.5-200	0.9995	2.5-200	0.9993	2.5-100	0.9996
485	Triasulfuron	2.5-200	0.9972	2.5-200	0.9997	5-100	0.9994	2.5-200	0.9996	2.5-250	0.9991
486	Triazamate	5-100	0.9938	2.5-200	0.9869	2.5-200	0.9926	2.5-200	0.9910	2.5-100	0.9947
487	Triazophos	5-100	0.9946	5-100	0.9943	2.5-200	0.9901	2.5-100	0.9893	2.5-100	0.9918
488	Tribufos	5-100	0.9972	2.5-200	0.9971	2.5-200	0.9953	2.5-200	0.9912	2.5-100	0.9975
489	Tricyclazole	2.5-100	0.9891	2.5-200	0.9892	2.5-200	0.9846	2.5-200	0.9925	2.5-250	0.9850
490	Trifloxystrobin	2.5-200	0.9909	2.5-200	0.9941	2.5-200	0.9941	2.5-200	0.9943	2.5-250	0.9938
491	Trifloxysulfuron	2.5-200	0.9964	2.5-200	0.9967	2.5-100	0.9975	2.5-200	0.9951	2.5-250	0.9957
492	Triflumizole	2.5-200	0.9929	5-100	0.9982	2.5-200	0.9948	2.5-200	0.9967	2.5-250	0.9939
493	Triflumuron	2.5-200	0.9865	2.5-100	0.9924	2.5-100	0.9936	2.5-100	0.9935	2.5-100	0.9944
494	Triflusulfuron metabolite (IN-M7222)	2.5-200	0.9822	2.5-200	0.9931	2.5-200	0.9897	2.5-200	0.9951	2.5-250	0.9869
495	Trinexapac-ethyl	2.5-200	0.9906	2.5-200	0.9914	2.5-200	0.9928	2.5-200	0.9934	2.5-250	0.9907
496	Triticonazole	2.5-200	0.9927	2.5-200	0.9939	2.5-200	0.9925	2.5-200	0.9939	2.5-100	0.9969
497	Tritosulfuron metabolite (M635H004)	2.5-100	0.9884	2.5-200	0.9927	2.5-200	0.9945	2.5-200	0.9978	2.5-250	0.9937
498	TZ-1E	2.5-200	0.9923	2.5-200	0.9936	5-200	0.9993	2.5-200	0.9944	2.5-100	0.9970
499	Uniconazole	2.5-200	0.9931	2.5-200	0.9939	2.5-200	0.9920	2.5-200	0.9958	2.5-250	0.9907
500	Valifenalate	2.5-100	0.9929	2.5-100	0.9928	2.5-200	0.9949	2.5-200	0.9907	2.5-100	0.9948
501	Vamidothion	2.5-100	0.9936	2.5-200	0.9898	2.5-100	0.9987	2.5-200	0.9964	2.5-250	0.9888
502	Vernolate	2.5-200	0.9979	2.5-100	0.9991	2.5-200	0.9986	2.5-200	0.9987	2.5-250	0.9983
503	XMC	2.5-100	0.9940	2.5-100	0.9965	5-200	0.9956	2.5-200	0.9936	2.5-100	0.9961
504	Zoxamide	2.5-100	0.9934	2.5-200	0.9882	2.5-100	0.9952	5-100	0.9922	2.5-250	0.9865

Table S3. Recovery ranges (100 μg/kg) of pesticides exhibiting generally low recovery rates in the representative crops and their chemical moieties.

Chemical moiety	Compound name	Recovery range in five crops
Carboxylic acid	Bispyribac	31.8–87.2
	Florpyrauxifen	16.4–24.3
	Isoxadifen	20.5–32.7
	Trinexapac-ethyl	31.0–46.2
Carboxylic acid (imidazolinone)	Imazamox	15.1–23.3
	Imazamox metabolite (M720H001)	7.4–14.4
	Imazapic	10.7–22.6
	Imazaquin	13.7–22.7
	Imazethapyr	11.4–26.1
Thiocarboxylic acid	Acibenzolar acid	21.2–41.5
Sulfonamide (carbamate)	Asulam	42.1–52.6
Sulfonamide (pyrimidine)	Diclosulam	71.5–78.5
	Nicosulfuron	36.5–46.1
Sulfonamide (urea)	Ethametsulfuron-methyl	61.7–69.8
	Flazasulfuron	60.7–63.1
	Foramsulfuron	34.5–42.1
	Imazosulfuron	64.0–75.7
	Mesosulfuron-methyl	63.8–76.1
	Metosulam	53.5–65.1
	Propyrisulfuron	65.9–75.4
	Rimsulfuron	58.8–74.9
	Thifensulfuron-methyl	64.2–71.1
	Triasulfuron	54.3–68.4
	Trifloxysulfuron	58.6–64.7
Guanine	Cyromazine	20.4–60.3
Neutral (sulfoxide)	Clethodim sulfoxide	42.5–63.7
Neutral (sulfone)	Probenazole	16.5–73.5
Neutral (triketone sulfone)	Lancotrione	26.3–44.0
	Mesotrione	26.5–45.2
	Tefuryltrione	19.2–38.5
